# Effects of allergic diseases and age on the composition of serum IgG glycome in children

**DOI:** 10.1038/srep33198

**Published:** 2016-09-12

**Authors:** Marija Pezer, Jerko Stambuk, Marija Perica, Genadij Razdorov, Ivana Banic, Frano Vuckovic, Adrijana Miletic Gospic, Ivo Ugrina, Ana Vecenaj, Maja Pucic Bakovic, Sandra Bulat Lokas, Jelena Zivkovic, Davor Plavec, Graham Devereux, Mirjana Turkalj, Gordan Lauc

**Affiliations:** 1 Genos Glycoscience Research Laboratory, Zagreb, Croatia; 2 Children’s Hospital Srebrnjak, Zagreb, Croatia; 3 University of Zagreb, Faculty of Pharmacy and Biochemistry, Zagreb, Croatia; 4 University of Osijek, Faculty of Medicine, Osijek, Croatia; 5 Child Health, University of Aberdeen, Aberdeen, UK

## Abstract

It is speculated that immunoglobulin G (IgG) plays a regulatory role in allergic reactions. The glycans on the Fc region are known to affect IgG effector functions, thereby possibly having a role in IgG modulation of allergic response. This is the first study investigating patients’ IgG glycosylation profile in allergic diseases. Subclass specific IgG glycosylation profile was analyzed in two cohorts of allergen sensitized and non-sensitized 3- to 11-year-old children (conducted at University of Aberdeen, UK and Children’s Hospital Srebrnjak, Zagreb, Croatia) with 893 subjects in total. IgG was isolated from serum/plasma by affinity chromatography on Protein G. IgG tryptic glycopeptides were analyzed by liquid chromatography electrospray ionization mass spectrometry. In the Zagreb cohort IgG glycome composition changed with age across all IgG subclasses. In both cohorts, IgG glycome composition did not differ in allergen sensitized subjects, nor children sensitized to individual allergens, single allergen mean wheal diameter or positive wheal sum values. In the Zagreb study the results were also replicated for high total serum IgE and in children with self-reported manifest allergic disease. In conclusion, our findings demonstrate no association between serum IgG glycome composition and allergic diseases in children.

Immunoglobulin G (IgG) is involved in a number of immune response pathways, for example physiologically protecting against invading pathogens or pathologically, inducing inflammation and tissue destruction in autoimmune disorders. IgG molecules bind their antigen targets via the fragment antigen binding (Fab) domain, and exert their effector functions via the fragment crystallizable (Fc) domain. This dual binding capacity makes IgG a link between innate and adaptive immunity. By binding to receptors specific for its Fc region, Fcγ receptors (FcγRs), expressed on the surface of innate immune cells (such as monocytes, macrophages, neutrophils and natural killer cells) and B cells, IgG is involved in the regulation of both, the innate and adaptive arms of the immune response. Intriguingly, IgG molecules can initiate both a pro-inflammatory response by binding to activating FcγRs on innate immune cells and the complement system, as well as an anti-inflammatory response by binding to DC–specific ICAM-3–grabbing non-integrin receptor (DC-SIGN)^1^.

Each IgG molecule contains two biantennary N-glycans covalently attached to conserved N-glycosylation sites at Asn-297 on each of its heavy chains. The most complex glycan contains 13 monosaccharide units and represents a biantennary digalactosylated and disialylated complex glycan with a bisecting β(1,4) *N-*acetylglucosamine (GlcNAc) and an α(1,6) fucose attached to core GlcNAc (Fig. 1). The remaining IgG glycans correspond to this tridecasaccharide with the lack of one or more sugar units. Polyclonal IgG glycosylation varies markedly in different physiological (age, sex, hormonal status) and pathological states (infectious, inflammatory and autoimmune diseases; cancers)^2^,^3^,^4^,^5^,^6^,^7^,^8^,^9^,^10^,^11^,^12^,^13^,^14^,^15^,^16^,^17^.

Glycans and glycan binding molecules play a major role in immune system regulation^18^ as do IgG glycans. They are of key importance for structural stabilization of the Fc region as well as for the IgG effector functions, affecting IgG binding affinity for FcγRs and other receptors^19^,^20^,^21^,^22^,^23^. Since activating and inhibiting FcγRs can modulate activation thresholds for immune effector cells^24^, IgG Fc glycans thus play an important role in immune response regulation in many conditions. For instance, variation in IgG Fc N-glycan are known to affect the activity of therapeutic antibodies and intravenous immunoglobulin preparations^25^,^26^.

Allergies are considered a harmful consequence of a misdirected immune response that evolved to protect us from macroparasites and non-infectious but harmful environmental factors^27^. In recent decades the prevalence of allergic diseases (allergic rhinoconjunctivitis, allergic asthma, atopic dermatitis, food allergies) has risen dramatically worldwide, particularly in developed countries, and in children^28^. The key effector molecule for the initiation of allergic cascade is allergen specific IgE, the synthesis of which is induced by exposure to common environmental antigens in atopic individuals. Allergen specific IgE is the key effector molecule for the initiation of allergic cascade. It binds to IgE specific receptors on the surface of mast cells and basophils, inducing their activation upon subsequent allergen challenge^29^. The sensitization phase, i.e. the presence of allergen specific IgE, is usually unnoticed, due to absence of clinical signs of allergy^30^, but is a prerequisite for later allergic response upon contact with the same allergen. In the clinical setting, sensitization is confirmed by *in vivo* and *in vitro* immunological tests, such as allergen skin prick tests (SPT) and enzyme-linked immunosorbent assay based assays for serum total and allergen specific IgE.

IgG is thought to play an inhibitory role, negatively modulating or completely abrogating IgE mediated allergic reactions^31^,^32^. This is most likely to be mediated by IgG binding to FcγRs expressed on the surface of tissue mast cells and peripheral blood basophils, the key effector cells in the immediate hypersensitivity reaction. Since IgG glycosylation is known to modulate IgG affinity for FcγRs on immune effector cells^33^, the question arose as to whether IgG glycans are involved in the modulation of allergic response. We speculated that IgG glycosylation might play a modulatory role in IgG-mediated control of allergic reaction during allergic sensitization and/or during allergic disease manifestation. If correct, one would expect to find a difference in IgG glycome composition associated with allergic sensitization and/or manifest allergic disease.

We conducted this study in order to test the hypothesis that IgG glycosylation plays a modulatory role in the IgG mediated control of allergic reaction during the sensitization phase. It is the first study exploring IgG glycosylation in allergic diseases – examining serum IgG glycoprofiles in two sizeable pediatric populations: 284 subjects at University of Aberdeen, UK and 609 subjects at Children’s Hospital Srebrnjak, Zagreb, Croatia. Since the vast majority of IgG glycosylation studies are performed on adult subjects, we used this study to additionally examine possible age-dependent IgG glycosylation patterns in children.

## Results

**Figure 2:**
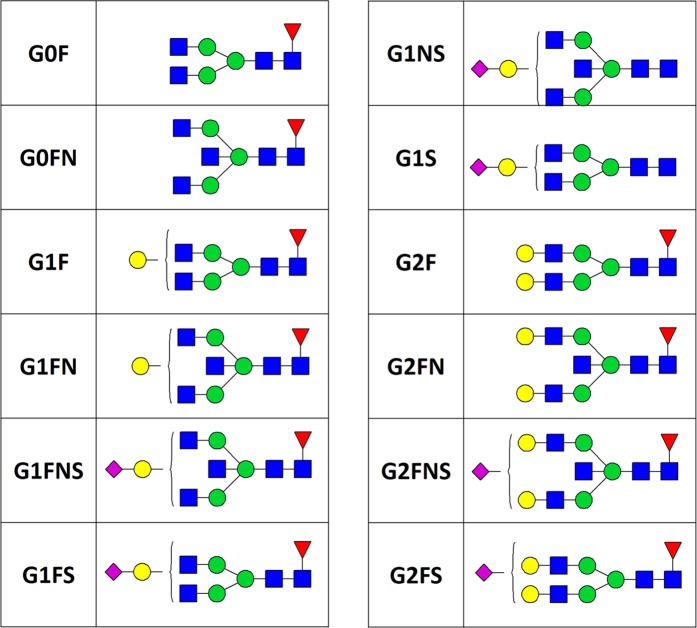
The most prominent glycan structures attached to the conserved N-glycosylation site on each of the two IgG heavy chains. *The legend, linkages and anomeric configurations are consistent with those depicted in* Fig. 1 *throughout all glycoforms.* Derived properties were calculated as follows: G0 = proportion of agalactosylated structures in total subclass glycans (G0 = G0F + G0FN). G1 = proportion of monogalactosylated structures in total subclass glycans (G1 = G1F + G1FN). G2 = proportion of digalactosylated structures in total subclass glycans (G2 = G2F + G2FN). S = proportion of sialylated structures in total subclass glycans (S = G1FS + G1FNS + G1S + G1NS + G2FS + G2FNS). F = proportion of fucosylated structures in total subclass glycans (F = G0F + G0FN + G1F + G1FN + G1FS + G1FNS + G2F + G2FN + G2FS + G2FNS). N = proportion of structures with bisecting *N-*acetylglucosamine in total subclass glycans (N = G0FN + G1FN + G1FNS + G1NS + G2FN + G2FNS).

**Figure 3:**
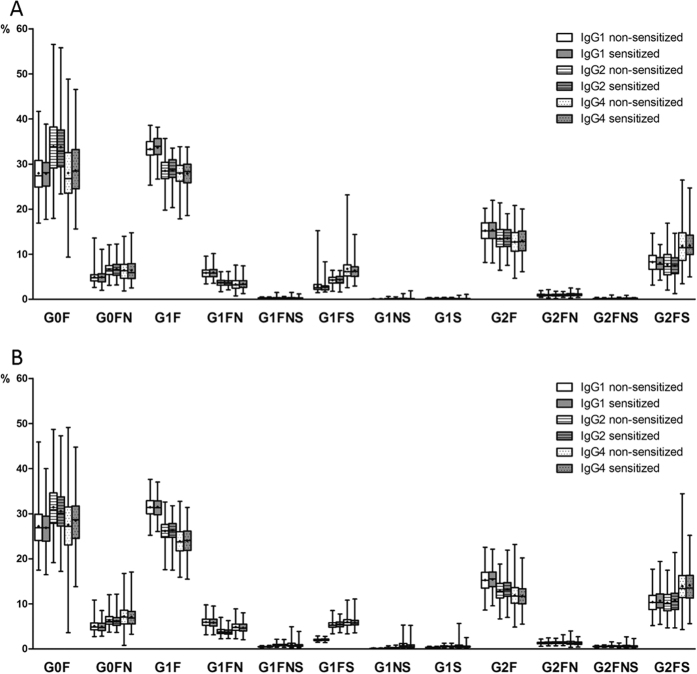
No difference in subclass specific abundance of specific IgG glycoforms in sensitized and non-sensitized children. Percentages of glycoforms in total subclass glycans are shown. Data are shown as box and whiskers plots. Each box represents the 25^th^ to 75^th^ percentiles. Lines inside the boxes represent the median. ‘+’s inside the boxes represent the mean. The whiskers represent the lowest and highest values. Analysis of associations between sensitization status and glycopeptide traits were performed using a regression model with age and sex included as additional covariates. (**A**) Aberdeen population (**B**) Zagreb population.

**Figure 4:**
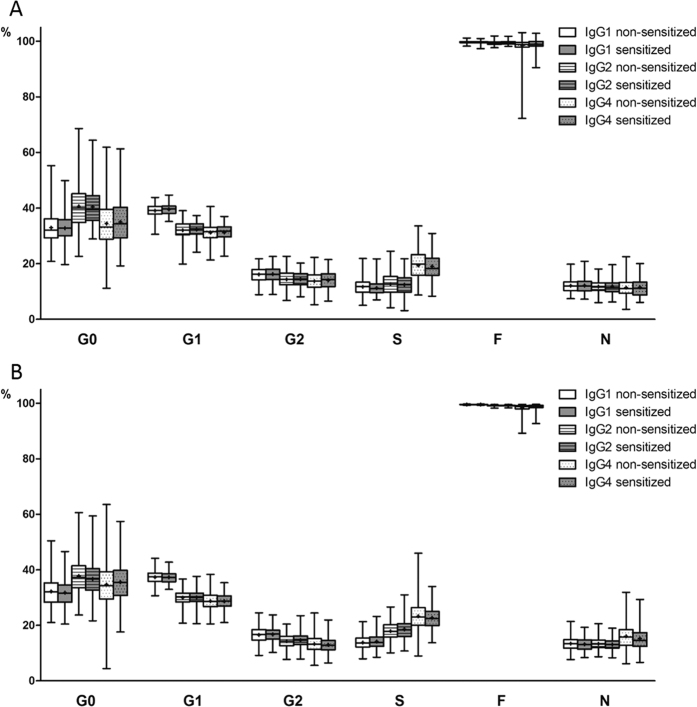
No difference in subclass specific IgG glycosylation pattern in sensitized and non-sensitized children. G0 = proportion of agalactosylated structures in total subclass glycans. G1 = proportion of monogalactosylated structures in total subclass glycans. G2 = proportion of digalactosylated structures in total subclass glycans. S = proportion of sialylated structures in total subclass glycans. F = proportion of fucosylated structures in total subclass glycans. N = proportion of structures with bisecting *N-*acetylglucosamine in total subclass glycans. Data are shown as box and whiskers plots. Each box represents the 25^th^ to 75^th^ percentiles. Lines inside the boxes represent the median. ‘+’s inside the boxes represent the mean. The whiskers represent the lowest and highest values. Analysis of associations between sensitization status and glycopeptide traits were performed using a regression model with age and sex included as additional covariates. (**A**) Aberdeen population (**B**) Zagreb population.

Subclass specific IgG composition was examined in allergen sensitized and non-sensitized children in the Aberdeen and Zagreb cohorts. No difference in IgG glycosylation pattern (12 main glycan species and 6 derived traits, Fig. 2) was found between children sensitized to at least one allergen and non-sensitized children in either of the two cohorts (Figs 3 and 4, Supplemental Tables 1 and 2). Moreover, no association was found between IgG glycosylation pattern and sensitization to any single allergen, single allergen mean wheal diameter or positive wheal sum values (Supplemental Tables 1 and 2).

**Figure 5:**
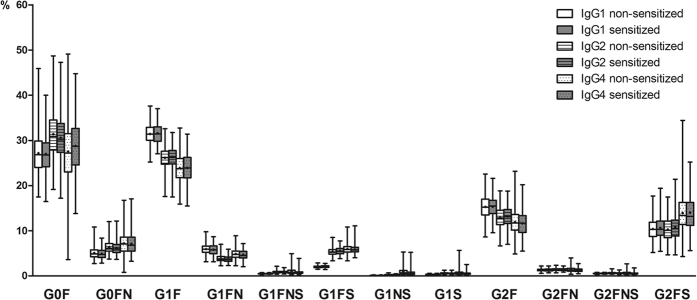
No difference in subclass specific abundance of specific IgG glycoforms in sensitized (SPT positive and high total serum IgE) and non-sensitized children in Zagreb population. Percentages of glycoforms in total subclass glycans are shown. Data are shown as box and whiskers plots. Each box represents the 25^th^ to 75^th^ percentiles. Lines inside the boxes represent the median. ‘+’s inside the boxes represent the mean. The whiskers represent the lowest and highest values. Analysis of associations between sensitization status and glycopeptide traits were performed using a regression model with age and sex included as additional covariates.

**Figure 6:**
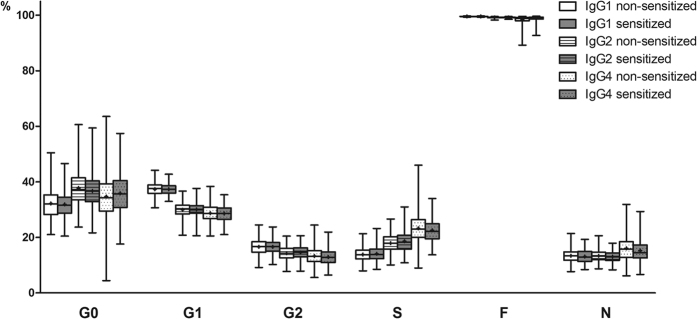
No difference in subclass specific IgG glycosylation pattern in sensitized (SPT positive and high total serum IgE) and non-sensitized children in Zagreb population. G0 = proportion of agalactosylated structures in total subclass glycans. G1 = proportion of monogalactosylated structures in total subclass glycans. G2 = proportion of digalactosylated structures in total subclass glycans. S = proportion of sialylated structures in total subclass glycans. F = proportion of fucosylated structures in total subclass glycans. N = proportion of structures with bisecting *N-*acetylglucosamine in total subclass glycans. Data are shown as box and whiskers plots. Each box represents the 25^th^ to 75^th^ percentiles. Lines inside the boxes represent the median. ‘+’s inside the boxes represent the mean. The whiskers represent the lowest and highest values. Analysis of associations between sensitization status and glycopeptide traits were performed using a regression model with age and sex included as additional covariates.

Two subsets of children were further established in the Zagreb study: sensitized children with high total serum IgE values, and non-sensitized children with normal total serum IgE values. In accordance with previous results, no difference in IgG glycosylation pattern (12 main glycan species and 6 derived traits) was found between the two groups of children (Figs 5 and 6, Supplemental Table 2). Moreover, in the Zagreb study no association was found between IgG glycosylation pattern and high level of total serum IgE (Supplemental Table 2).

In the Zagreb cohort no apparent difference in IgG glycosylation pattern (6 derived traits) was found in children suffering from allergic diseases (allergic asthma, allergic rhinitis, allergic rhinoconjunctivitis, atopic dermatitis) in the last 12 months compared to healthy children. The same result was obtained when high serum total IgE alone or high serum total IgE in the presence of positive SPT were used to confirm present manifested disease in addition to the disease present in the last 12 months. (Supplemental Figs 1–15).

After establishing that allergic sensitization and manifest allergic disease are not associated with the IgG glycome composition, we evaluated the effects of age on the combined dataset from the Zagreb cohort (Table 1, Supplemental Fig. 16). The content of monogalactosylated structures was shown to increase with age across all IgG subclasses. This was accompanied by a decrease in agalactosylated and an increase in digalactosylated structures in IgG4 subclass only. The level of sialylation decreased with age in IgG1 and IgG2. In addition, an increase in bisecting N-GlcNAc content accompanied by a decrease in core fucose content was found in IgG1 and the opposite effect (a decrease in bisecting N-GlcNAc content and an increase in core fucose content) in IgG4.

## Discussion

We report here the first study comparing the immunoglobulin glycosylation profiles of normal subjects with subjects suffering from allergic diseases. Our large scale study of 893 children was performed on two different populations with comparable results. In the two cohorts IgG glycosylation pattern was not altered in allergen sensitized subjects, nor was it altered in respect to sensitization to any single allergen, single allergen mean wheal diameter or positive wheal sum values. After narrowing the definition of sensitization in one of the cohorts (Zagreb) by the inclusion of serum total IgE, no differences were found between sensitized and control children. The same result, although not statistically confirmed, was found in the same cohort when children suffering from allergic diseases in the last 12 months and children likely suffering from allergic disease were compared to control children.

Since the two studies were designed independently and opportunistically used for IgG glycome analysis, there are slight methodological differences between them. The subject age ranges from 3 to 11 in the Zagreb study, compared to 10 to 11 in the Aberdeen study. Following blood withdrawal plasma was separated in the Aberdeen and serum in the Zagreb study. Different allergens and different commercially available allergen extract preparations were used in each study, and in the Zagreb study the allergens used differed between regions. It should be noted that the allergens used in each study were those recognized as the most common allergens for the local population. The lack of perfect standardization between the two studies might diminish the significance of comparative data we present here.

Given the limited number of allergens used to identify sensitized children, children sensitized to less common allergens would have been misclassified as non-sensitized and allocated to the control group, i.e. false negatives representing a null bias. To address this, in the Zagreb study, total serum IgE was also used to discriminate between sensitized and non-sensitized children. The children with positive SPT and high IgE were then compared to children with negative SPT results and a normal total serum IgE level; the results of this analysis did not differ from the main analysis.

The two methods used to distinguish sensitized vs. non-sensitized children (SPT vs. total serum IgE measurement) are somewhat different regarding the information they provide. While the total serum IgE measurement only gives the information on serum IgE content, the SPT represents a simulation of *in vivo* reaction to allergen, including release of various mediators by mast cells.

The lack of association between IgG glycoprofile and sensitization is only pertinent to the early stages of atopic disease before the development of allergic inflammation and manifest clinical allergic disease. Our initial analysis did not exclude the possibility that the total serum IgG glycome might be associated with later stages of atopic disease when clinical evident allergic disease is manifest, indeed it has been reported that total serum IgG glycome composition is associated with some diseases with an inflammatory component^13^,^15^,^16^,^34^. Although not part of our original study design, and therefore not statistically tested, after obtaining the results on IgG glycosylation with respect to allergic sensitization status, we compared the IgG glycosylation patterns in children suffering from allergic diseases in the last 12 months (self-reported by parents via ISAAC questionnaire) and sensitized children suffering from allergic disease (self-reported allergic disease in the last 12 months + allergen sensitization, as confirmed by elevated total serum IgE level alone or in addition to a positive SPT result) with control children in one of the cohorts (Zagreb). We again found no difference in IgG glycome composition in either of these groups when compared to control children suggesting that the IgG glycome is not associated with the early sensitization stage of atopic disease not the or later stages of clinical allergic disease.

Since 1985, when changes in IgG glycome composition were reported in rheumatoid arthritis (RA)^14^ IgG glycome alterations have been observed in many various diseases, particularly in inflammatory and autoimmune diseases^35^,^36^,^37^,^38^,^39^,^40^. We have reported significant differences in IgG glycosylation patterns in numerous large scale studies including patients suffering from acute systemic inflammation^13^, inflammatory bowel disease (ulcerative colitis and Crohn’s disease)^16^, systemic lupus erythematosus^15^, RA (unpublished data) and type II diabetes (unpublished data), all diseases of a severely activated and/or skewed immune response. We have also found significant differences in IgG glycoprofiles in patients with renal dysfunction and colorectal cancer^17^,^41^. Since allergic diseases also result from an imbalance in the immune response, it came as a surprise that no changes in IgG glycome in any of its subclasses were found in the allergen sensitized population and population of children suffering from allergic diseases. An obvious possible contributory factor to the absence of association in the current study and the positive associations in our previous studies could be that the current study was based on two populations studies of children whereas our previous work were case-control investigations of adults. Our results suggest that total serum IgG glycans do not have a significant role in the development and progression of allergic diseases however further epidemiological as well as functional studies are required to confirm or refute our findings.

To our knowledge this is the one of very few studies examining IgG glycosylation profile of a children’s population in any disease. The direction of IgG glycome changes in healthy subjects differs in adults and in children, which makes independent age group analyses a necessity. In general, the IgG glycoform distribution found in this study is consistent with our previous reports of the IgG glycoprofile of healthy children. With increasing age there was a decrease of agalactosylated and core fucosylated structures, accompanied by an increase of digalactosylated structures and structures bearing a bisecting GlcNAc. It should be noted however that our previous work investigated an older pediatric population and used a different methodology that resulted in total IgG glycans (Fab and Fc combined, all subclasses)^42^. In a healthy adult, 10–15% of total serum IgG contain terminal sialic on one or both of its antennae^43^. Fc terminal sialylation is established as a possible switch between IgG pro- and anti-inflammatory activity^44^,^45^. Unfortunately, due to methodological constraints we could not examine the content of disialylated IgG in our two cohorts^46^.

The existence of inherent differences in IgG subclass specific glycosylation has already been confirmed^5^. The different subclass glycosylation profiles found in our study, particularly in IgG4 compared to the other subclasses, probably underline their different biological role. However, for IgG4 it might be relevant that the observed differences could have been a consequence of decreased analytical precision due to the low concentrations of IgG4 compared to other IgG subclasses. More work is required, particularly on antigen specific IgG subclasses, to improve our understanding of the role of IgG glycosylation in allergic diseases.

Allergen specific IgG is present in serum of allergic patients, mostly of IgG1 and IgG4 subclass^47^,^48^. Allergen specific IgG4 is thought to have a protective role in allergic diseases, and is also one of mechanisms of action of allergen specific immunotherapy, the only curative approach to the treatment of allergies. Allergen specific IgG4 is believed to have multiple protective roles in IgE mediated allergic diseases: blocking the allergen and/or modulating the allergic response via inhibitory FcγRIIB on the surface of mast cells and B cells. It would therefore be of particular interest to analyze the glycome composition of allergen specific IgG, and particularly of allergen specific IgG4 in allergic patients.

## Methods

### Subjects

101 sensitized (54 male, median age 10.3 years, range 10.0–10.9 and 47 female, median age 10.3 years, range 9.9–11.1) and 183 non-sensitized (80 male, median age 10.3 years, range 9.9–10.8 and 103 female, median age 10.3, range 9.9–11.0) children who had participated in the detailed assessment phase of the 10 year follow up of the population based SEATON cohort study in Aberdeen, UK (described in detail elsewhere^49^,^50^,^51^) were included in this study. The study was approved by the North of Scotland Research Ethics Committee, and written informed parental consent and written assent of the child was obtained. All the methods were carried out in accordance with the approved guidelines.

In addition, 237 sensitized (149 male, median age 8 years, range 4–11 and 88 female median age 8 years, range 4–11) and 372 non-sensitized children (172 male, median age 8 years, range 4–11; 200 female, median age 8 years, range 3–11) from Children’s Hospital Srebrnjak (CHS), Zagreb, Croatia were included in this study. Children were recruited in Croatian kindergartens (ages 3–6) and primary schools (ages 7–10) with institutional consent and informed parental consent. Clinical data were collected from parents of all children using a standardized International Study of Asthma and Allergies in Childhood (ISAAC) questionnaire^52^. The questionnaire is self-reported by parents to evaluate allergic asthma, allergic rhinitis, allergic rhinoconjunctivitis and atopic dermatitis in childhood. The study was approved by the CHS Ethics Committee. All the methods were carried out in accordance with the approved guidelines.

### Skin prick test

Within the scope of both studies, skin prick testing was performed to determine allergic sensitization to most common local allergens. SPT was performed on the volar surface of the non-dominant forearm with the most common local allergen extracts.

In the Aberdeen study skin prick reactivity to the dog, cat, timothy grass, egg, peanut, and house dust mite (*Dermatophagoides pteronysinnus)* allergens was determined using commercially available Soluprick QC preparations (ALK Abello, UK) containing standardized allergen extracts. In the Zagreb study skin prick reactivity to the grasses mix (cocksfoot, sweet vernal-grass, rye-grass, meadow grass, and timothy), ragweed (*Ambrosia*), dog hair, cat fur, house dust mite (*Dermatophagoides pteronysinnus*), Cladosporium mix (*Cladosporium cladosporioides* and *C. herbarum*), pine, olive, *Parietaria*, trees mix (maple, horse-chestnut, plane tree and lime tree), birch and hazel allergens was determined by widely used Alyostal preparations (Stallergenes Greer, France) containing standardized allergen extracts. Depending on the region’s vegetation, pine, olive, birch, hazel and trees mix were omitted from the panel to avoid unnecessary testing. 0.9% saline was used as the negative and 10 mg/mL histamine as the positive control in both studies.

For both studies a positive SPT response for any given allergen was defined as a mean wheal diameter (the mean of the longest diameter and the diameter perpendicular to it at its mid-point) of at least 3 mm 15 minutes after inoculation. Positive wheal sum was calculated as the sum of all mean wheal diameters equal to or greater than 3 mm in a given patient.

### Peripheral blood collection and serum/plasma separation

Peripheral blood was collected by venepuncture. In the Aberdeen study blood was collected into EDTA coated vacutainers. Plasma was separated by centrifugation (5000 g, 10 min) and stored at −80 C until further analysis. In the Zagreb study blood was collected into vacutainers with clot activator and gel for serum separation. Serum was separated by centrifugation (3000 g, 10 min) and stored at −20 C until further analysis.

### Total IgE measurement

In addition to SPT, in Zagreb study the concentration of total IgE in serum was determined in all subjects by a sandwich fluorescent enzyme immunoassay - ImmunoCAP^53^. In house established age dependent cut-off values were used to determinate elevated serum total IgE level^54^. 169 children (110 male, median age 9, range 4–11 and 59 female, median age 8, range 4–11) were characterized as sensitized based on elevated total IgE value and a positive SPT, and 371 (172 male, median age 8, range 4–11 and 199 female, median age 8, range 3–11) as non-sensitized based on normal total IgE value and a negative SPT.

### IgG isolation

After defrosting and centrifugation (12100 g, 3 min or 5000 g, 10 min) lipid-free serum/plasma fraction was pipetted into 96-well plates. In the Aberdeen study all samples were randomized across the plates using block randomization and in the Zagreb study cases and controls were evenly distributed among the plates. All plates included standard and blank samples for quality control and batch correction. IgG was isolated from plasma by affinity chromatography on protein G monolithic plates (BIA Separations, Slovenia) as described previously^55^. Briefly, 100 μL serum/plasma was diluted 1:7 with 1 × PBS, pH 7.4, applied to the protein G plate and instantly washed with 1xPBS, pH 7.4, to remove unbound proteins. IgG was eluted with 1 mL 0.1 M formic acid (Merck, Germany) and neutralized with 170 μL 1 M ammonium bicarbonate (Merck, Germany).

### IgG tryptic digestion and purification

25 μg IgG was digested with 200 ng of trypsin at 37 °C (Worthington, USA) overnight. Resulting tryptic glycopeptides were purified by reverse phase solid phase extraction using Chromabond C18ec beads (Marcherey-Nagel, German). C18 beads were activated with 80% ACN containing 0.1% trifluoroacetic acid (TFA) (Sigma-Aldrich, USA) and conditioned with 0.1% TFA. Tryptic digests were diluted 10X with 0.1% TFA, loaded onto C18 beads, washed with 0.1% TFA and finally eluted with 20% ACN containing 0.1% TFA. Eluates containing tryptic glycopeptides were dried by vacuum centrifugation and dissolved with 20 μL of ultrapure water.

### LC-ESI-MS/MS analysis of IgG tryptic glycopeptides

Tryptic digests were analyzed on nanoACQUITY UPLC system (Waters, USA) coupled to Compact mass spectrometer (Bruker Daltonics, Bremen, Germany). 9 μL eluates containing IgG tryptic glycopeptides was loaded into Acclaim PepMap100 C8 (5 mm × 300 μm i.d.) trap column and washed 1 min with 0,1% TFA (solvent A) at a flow rate of 40 μL/min. Separation was achieved on a Halo C18 nano-LC column (150 mm × 75 μm i.d., 2.7 μm HALO fused core particles) (Advanced Materials technology, USA) using a 3,5 min gradient at a flow rate of 1 μL/min from 18% to 25% solvent B (80% ACN). Column temperature was 30 °C. Mass spectra were recorded from m/z 200 to 1900 with 2 averages at a frequency of 0,5 Hz. Quadrupole ion energy and collision energy of the MS were set at 4 eV. NanoACQUITY UPLC system and the Bruker micrOTOF-Q were operated under HyStar software version 3.2 software. In Caucasian populations, IgG2 and IgG3 tryptic Fc glycopeptides have identical peptide moieties and are therefore not distinguishable by this profiling method^56^,^57^. Data were extracted using an in-house python script. Briefly, data were m/z recalibrated using a subset of hand-picked analytes having a high signal-to-noise ratio and the expected isotopic distribution. After recalibration, intensities for top four isotopologues were extracted using 10 ppm m/z window. Based on top signals, retention times were aligned towards the cohort median. After defining retention time bins for analytes of interest, all of the signals belonging to a single analyte for every sample were summed up. The most prominent 12 glycopeptides that were present in each subclass were used for statistical analysis.

### Statistical analysis

In order to remove experimental variation from measurements, normalization and batch correction were performed on the LC-MS glycopeptide data. To make measurements across samples comparable, IgG-isoform specific normalization by total area was performed. Prior to batch correction, normalized glycopeptide measurements were log-transformed due to right skewness of their distributions and the multiplicative nature of batch effects. Batch correction was performed on log-transformed measurements using the ComBat method, in which the technical source of variation (which sample was analyzed on which plate) was modeled as a batch covariate. To get measurements corrected for experimental noise, estimated batch effects were subtracted from log-transformed measurements. For each isoform, in addition to 12 directly measured glycopeptide structures, 6 derived traits were calculated from the directly measured glycopeptides. These derived traits average particular glycosylation features (galactosylation, fucosylation, sialylation) across different individual glycan structures, and consequently they are more closely related to individual enzymatic activities and underlying genetic polymorphisms.

Analyses of associations between sensitization status and glycopeptide traits were performed using a regression model with age and sex included as additional covariates. In addition, a regression model adjusted for age and sex was used to examine associations between glycopeptide levels and other clinical traits (sensitization to a particular allergen, single allergen mean wheal diameter, positive wheal sum values and total serum IgE level in the Zagreb study). Additionally, associations between age and glycopeptide measurements were examined in the Zagreb cohort using a regression model.

Glycopeptide traits were described as dependent variable. Prior to analyses, glycopeptide variables were all transformed to a standard normal distribution (mean = 0, SD = 1) by inverse transformation of ranks to normality. Using rank-transformed variables in analyses makes estimated effects of different glycopeptides comparable, as transformed glycopeptide variables have the same standardized variance. The false discovery rate (FDR) for both analyses was controlled using the Benjamini-Hochberg procedure, and p values corrected for multiple testing (with FDR set at 0.05) are shown throughout.

## Additional Information

**How to cite this article**: Pezer, M. *et al*. Effects of allergic diseases and age on the composition of serum IgG glycome in children. *Sci. Rep.* **6**, 33198; doi: 10.1038/srep33198 (2016).

## Supplementary Material

Supplementary Information

## Figures and Tables

**Figure 1:**
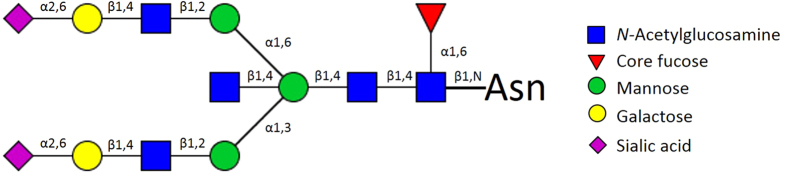
The most complex IgG Fc glycan. In heterogenous mixture of serum IgG N-glycans over 30 different glycan species are found. The most complex glycan contains 13 monosaccharide units and represents a biantennary digalactosylated and disialylated complex glycan with bisecting β(1,4) GlcNAc and an α(1,6) fucose attached to core GlcNAc. The remaining IgG glycans correspond to this tridecasaccharide with the lack of one or more sugar units.

**Table 1:** Effect of age on IgG glycome composition in the Zagreb population (see also Supplemental Fig. 16).

Glycopeptide trait	Effect	SE	p	p adjusted
IgG1 G1	↑ 0.2386	0.020488	1.55E-28	2.79E-27
IgG2 G1	↑ 0.2131	0.020948	1.23E-22	1.11E-21
IgG4 G1	↑ 0.1957	0.021228	4.17E-19	2.50E-18
IgG1 S	↓ −0.1379	0.021968	6.12E-10	2.75E-09
IgG1 F	↓ −0.1294	0.022053	6.79E-09	2.44E-08
IgG4 G0	↓ −0.1097	0.022228	9.77E-07	2.93E-06
IgG4 N	↓ −0.1060	0.022259	2.31E-06	5.94E-06
IgG4 G2	↑ 0.0942	0.022346	2.75E-05	6.19E-05
IgG2 S	↓ −0.0730	0.022477	1.19E-03	2.38E-03
IgG4 F	↑ 0.0590	0.022545	8.90E-03	1.60E-02
IgG1 N	↑ 0.0541	0.022565	1.66E-02	2.72E-02
IgG2 G0	−0.0473	0.022591	3.61E-02	5.42E-02
IgG1 G2	−0.0403	0.022613	7.46E-02	1.03E-01
IgG2 F	0.0293	0.022641	1.95E-01	2.51E-01
IgG1 G0	−0.0134	0.022666	5.54E-01	6.55E-01
IgG2 G2	0.0124	0.022667	5.83E-01	6.55E-01
IgG2 N	−0.0094	0.022669	6.78E-01	7.18E-01
IgG4 S	0.0059	0.022671	7.93E-01	7.93E-01

SE = standard error, G0 = proportion of agalactosylated structures in total subclass glycans. G1 = proportion of monogalactosylated structures in total subclass glycans. G2 = proportion of digalactosylated structures in total subclass glycans. S = proportion of sialylated structures in total subclass glycans. F = proportion of fucosylated structures in total subclass glycans. N = proportion of structures with bisecting *N-*acetylglucosamine in total subclass glycans. Glycopeptide traits that are affected by age are shown in bold. Associations between age and glycan measurements were examined using a regression model.
