# Supplementary material for: Effects of allergic diseases and age on the composition of serum IgG glycome in children
Source: Sci Rep. 2016 Sep 12;6:33198. doi: 10.1038/srep33198 (PMC5018987; doi:10.1038/srep33198)
Supplement: Supplementary Information [file srep33198-s1.pdf]

Supplemental Information

Effects of allergic diseases and age on the composition of serum IgG glycome in children

Marija Pezer[1*], Jerko Stambuk[1], Marija Perica[2], Genadij Razdorov[3], Ivana Banic[2], Frano Vuckovic[1], Adrijana Miletic Gospic[2], Ivo Ugrina[1], Ana Vecenaj[2], Maja Pucic Bakovic[1], Sandra Bulat Lokas[2], Jelena Zivkovic[2], Davor Plavec[2,4], Graham Devereux[5], Mirjana Turkalj[2,4**], Gordan Lauc[1,3**]

[1] Genos Glycoscience Research Laboratory, Zagreb, Croatia

[2] Children's Hospital Srebrnjak, Zagreb, Croatia

[3] University of Zagreb, Faculty of Pharmacy and Biochemistry, Zagreb, Croatia

[4] University of Osijek, Faculty of Medicine, Osijek, Croatia

[5] Child Health, University of Aberdeen, Aberdeen, UK

[*] mpezer@genos.hr

[**] shared last authorship

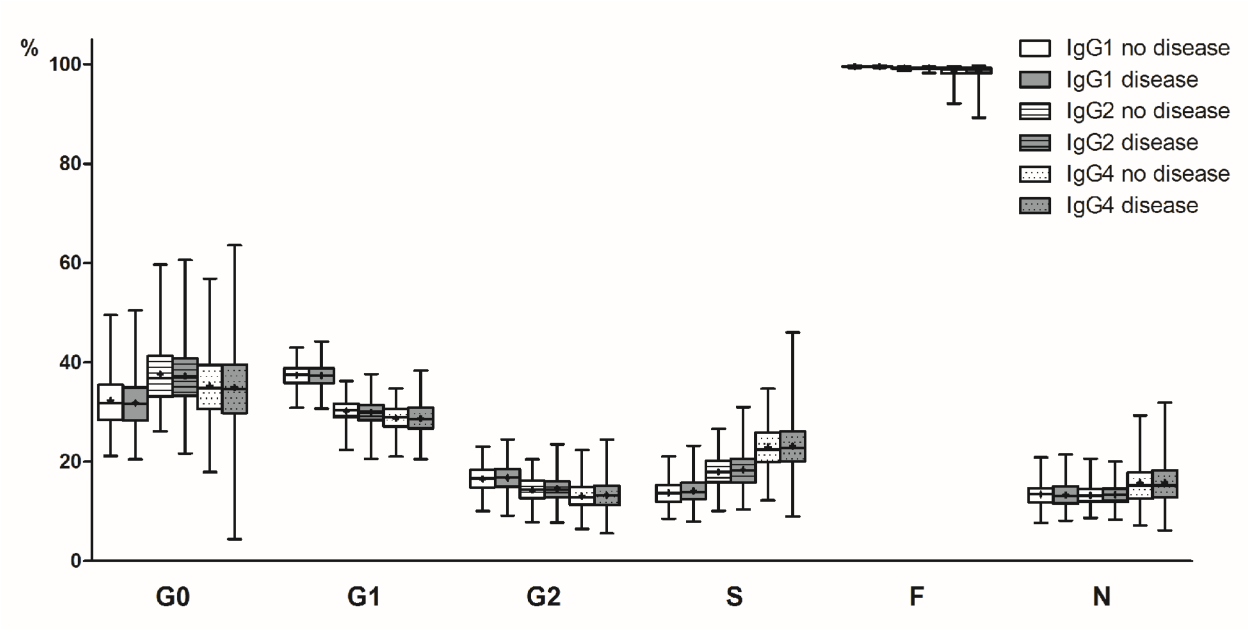

Supplemental Figure 1. No difference in subclass specific IgG glycosylation pattern in children suffering from allergic asthma and/or allergic rhinitis and/or allergic rhinoconjunctivitis and/or atopic dermatitis in the last 12 months (n=361) and control children (n=248) (based on ISAAC questionnaire). G0 = proportion of agalactosylated structures in total subclass glycans. G1 = proportion of monogalactosylated structures in total subclass glycans. G2 = proportion of digalactosylated structures in total subclass glycans. S = proportion of sialylated structures in total subclass glycans. F = proportion of fucosylated structures in total subclass glycans. N = proportion of structures with bisecting *N*-acetylglucosamine in total subclass glycans. Data are shown as box and whiskers plots. Each box represents the 25th to 75th percentiles. Lines inside the boxes represent the median. '+'s inside the boxes represent the mean. The whiskers represent the lowest and highest values.

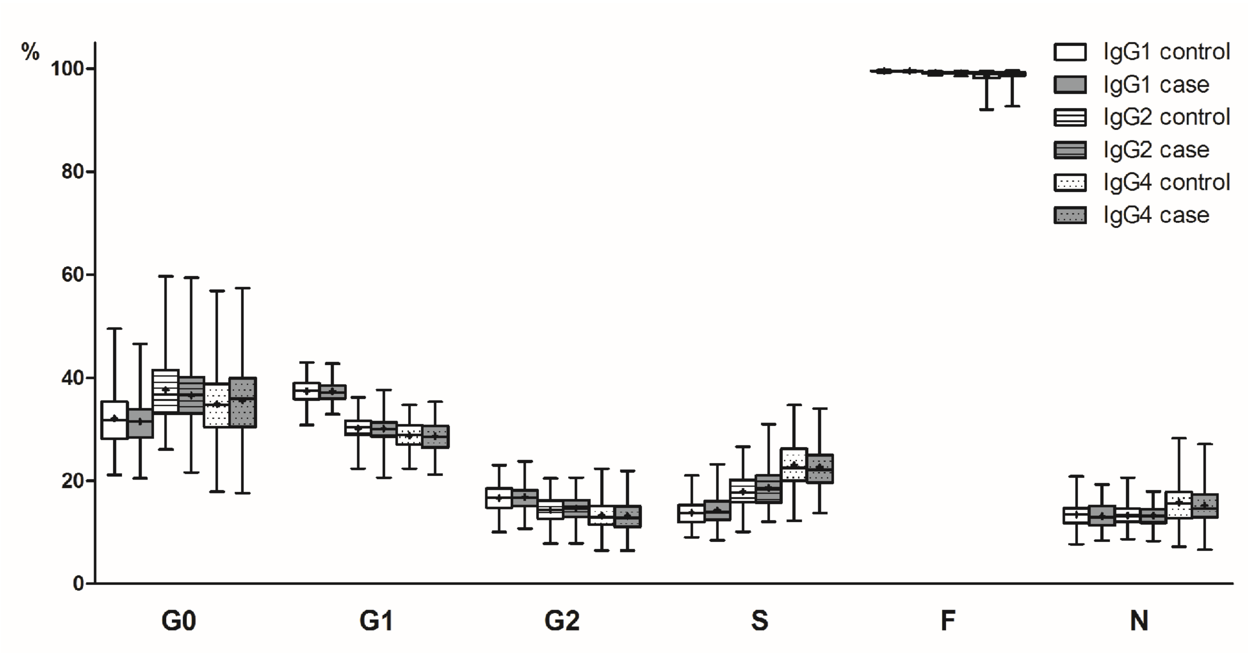

Supplemental Figure 2. No difference in subclass specific IgG glycosylation pattern in children with a high serum total IgE level and suffering from allergic asthma and/or allergic rhinitis and/or allergic rhinoconjunctivitis and/or atopic dermatitis in the last 12 months (n=129) and control children (n=207) (based on ISAAC questionnaire). G0 = proportion of agalactosylated structures in total subclass glycans. G1 = proportion of monogalactosylated structures in total subclass glycans. G2 = proportion of digalactosylated structures in total subclass glycans. S = proportion of sialylated structures in total subclass glycans. F = proportion of fucosylated structures in total subclass glycans. N = proportion of structures with bisecting *N*-acetylglucosamine in total subclass glycans. Data are shown as box and whiskers plots. Each box represents the 25th to 75th percentiles. Lines inside the boxes represent the median. '+'s inside the boxes represent the mean. The whiskers represent the lowest and highest values.

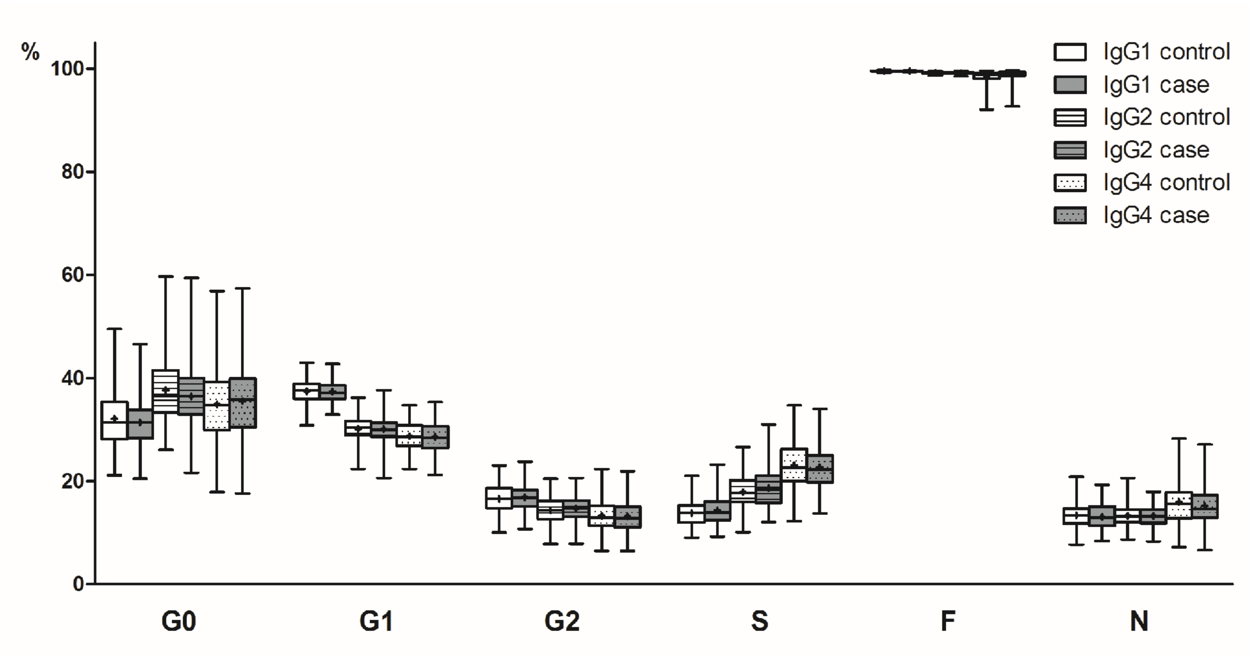

Supplemental Figure 3. No difference in subclass specific IgG glycosylation pattern in children with a high serum total IgE level, a positive skin prick test and suffering from allergic asthma and/or allergic rhinitis and/or allergic rhinoconjunctivitis and/or atopic dermatitis in the last 12 months (n=128) and control children (n=179) (based on ISAAC questionnaire). G0 = proportion of agalactosylated structures in total subclass glycans. G1 = proportion of monogalactosylated structures in total subclass glycans. G2 = proportion of digalactosylated structures in total subclass glycans. S = proportion of sialylated structures in total subclass glycans. F = proportion of fucosylated structures in total subclass glycans. N = proportion of structures with bisecting *N*-acetylglucosamine in total subclass glycans. Data are shown as box and whiskers plots. Each box represents the 25th to 75th percentiles. Lines inside the boxes represent the median. '+'s inside the boxes represent the mean. The whiskers represent the lowest and highest values.

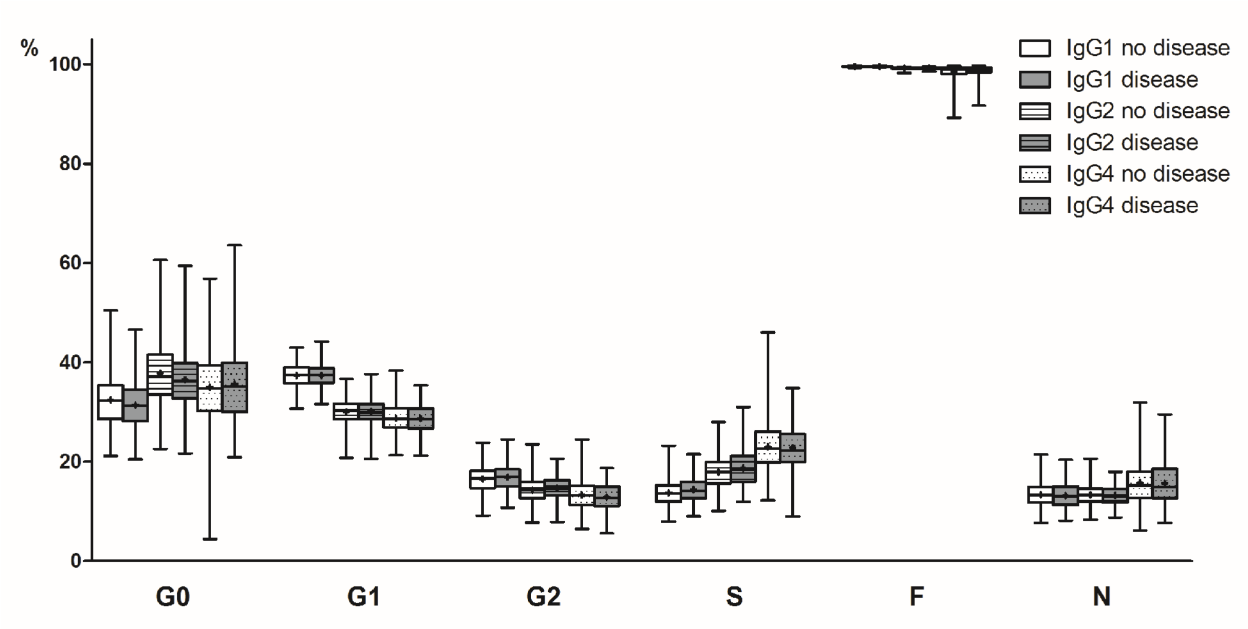

Supplemental Figure 4. No difference in subclass specific IgG glycosylation pattern in children suffering from allergic asthma in the last 12 months (n=150) and control children (n=409) (based on ISAAC questionnaire). G0 = proportion of agalactosylated structures in total subclass glycans. G1 = proportion of monogalactosylated structures in total subclass glycans. G2 = proportion of digalactosylated structures in total subclass glycans. S = proportion of sialylated structures in total subclass glycans. F = proportion of fucosylated structures in total subclass glycans. N = proportion of structures with bisecting *N*-acetylglucosamine in total subclass glycans. Data are shown as box and whiskers plots. Each box represents the 25th to 75th percentiles. Lines inside the boxes represent the median. '+'s inside the boxes represent the mean. The whiskers represent the lowest and highest values.

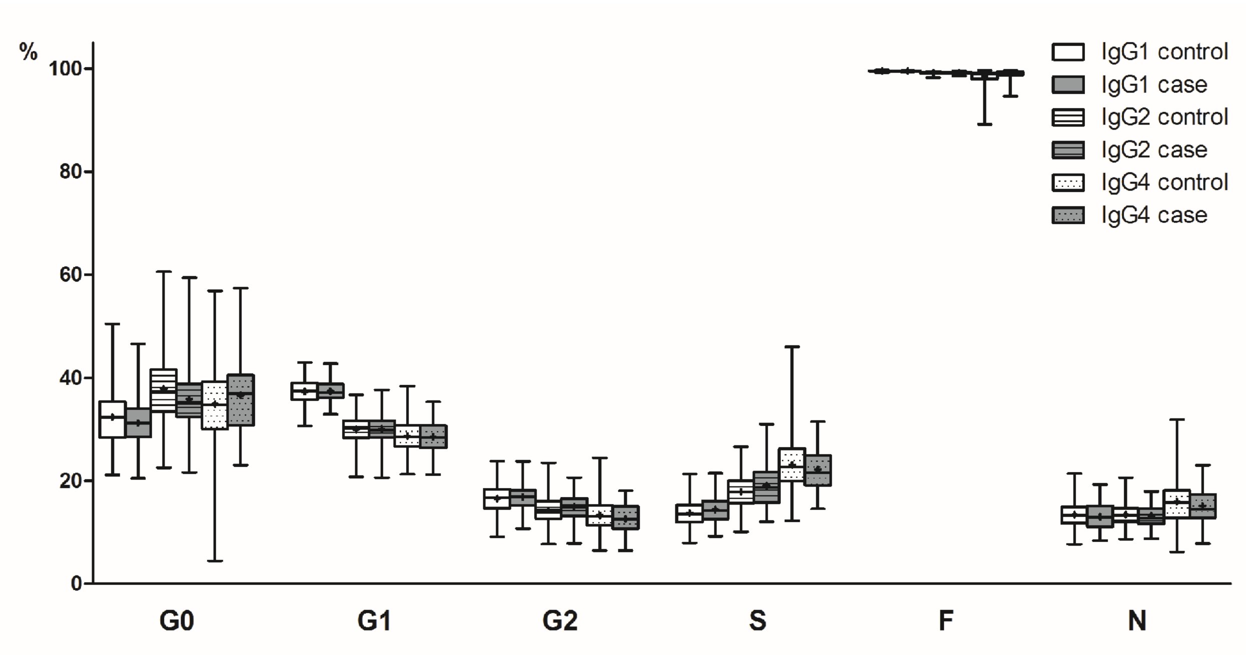

Supplemental Figure 5. No difference in subclass specific IgG glycosylation pattern in children with a high serum total IgE level and suffering from allergic asthma in the last 12 months (n=71) and control children (n=320) (based on ISAAC questionnaire). G0 = proportion of agalactosylated structures in total subclass glycans. G1 = proportion of monogalactosylated structures in total subclass glycans. G2 = proportion of digalactosylated structures in total subclass glycans. S = proportion of sialylated structures in total subclass glycans. F = proportion of fucosylated structures in total subclass glycans. N = proportion of structures with bisecting *N*-acetylglucosamine in total subclass glycans. Data are shown as box and whiskers plots. Each box represents the 25th to 75th percentiles. Lines inside the boxes represent the median. '+'s inside the boxes represent the mean. The whiskers represent the lowest and highest values.

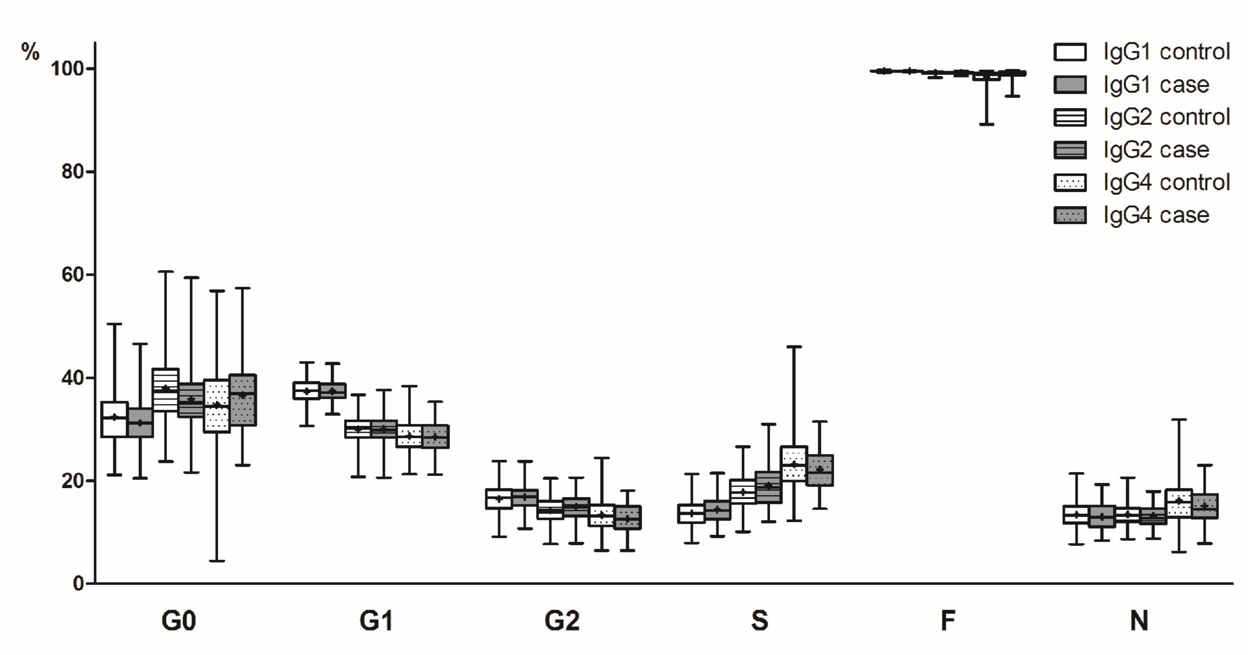

Supplemental Figure 6. No difference in subclass specific IgG glycosylation pattern in children with a high serum total IgE level, a positive skin prick test and suffering from allergic asthma in the last 12 months (n=71) and control children (n=271) (based on ISAAC questionnaire). G0 = proportion of agalactosylated structures in total subclass glycans. G1 = proportion of monogalactosylated structures in total subclass glycans. G2 = proportion of digalactosylated structures in total subclass glycans. S = proportion of sialylated structures in total subclass glycans. F = proportion of fucosylated structures in total subclass glycans. N = proportion of structures with bisecting *N*-acetylglucosamine in total subclass glycans. Data are shown as box and whiskers plots. Each box represents the 25th to 75th percentiles. Lines inside the boxes represent the median. '+'s inside the boxes represent the mean. The whiskers represent the lowest and highest values.

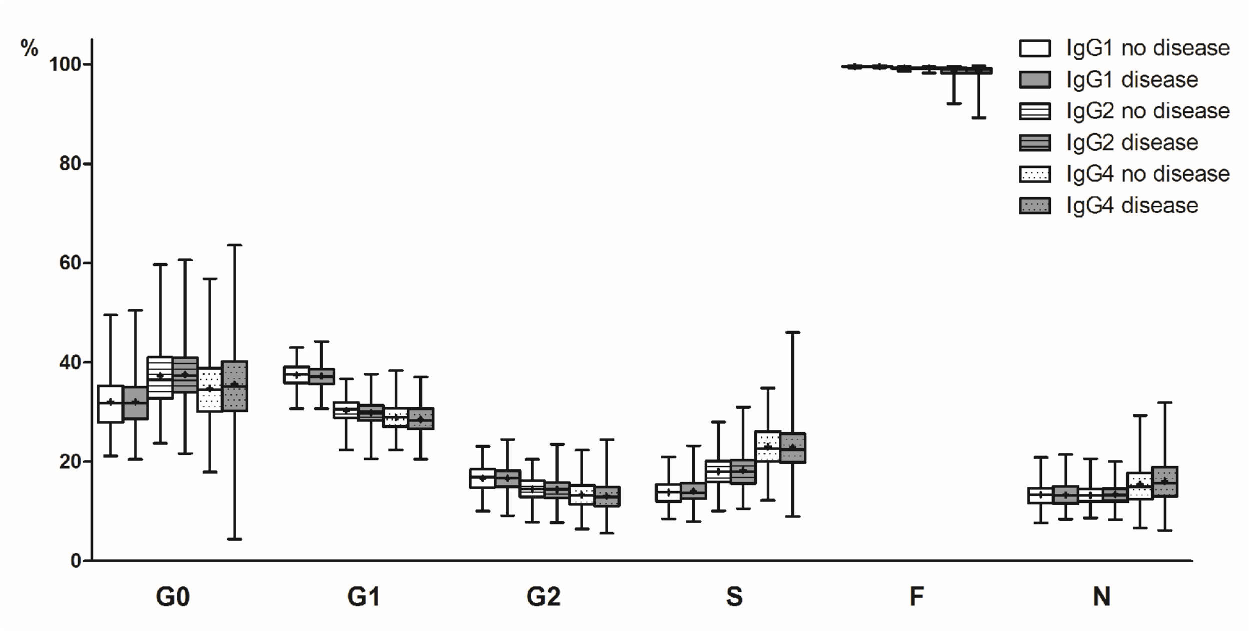

Supplemental Figure 7. No difference in subclass specific IgG glycosylation pattern in children suffering from allergic rhinitis (n=263) in the last 12 months and control children (n=307) (based on ISAAC questionnaire). G0 = proportion of agalactosylated structures in total subclass glycans. G1 = proportion of monogalactosylated structures in total subclass glycans. G2 = proportion of digalactosylated structures in total subclass glycans. S = proportion of sialylated structures in total subclass glycans. F = proportion of fucosylated structures in total subclass glycans. N = proportion of structures with bisecting *N*-acetylglucosamine in total subclass glycans. Data are shown as box and whiskers plots. Each box represents the 25th to 75th percentiles. Lines inside the boxes represent the median. '+'s inside the boxes represent the mean. The whiskers represent the lowest and highest values.

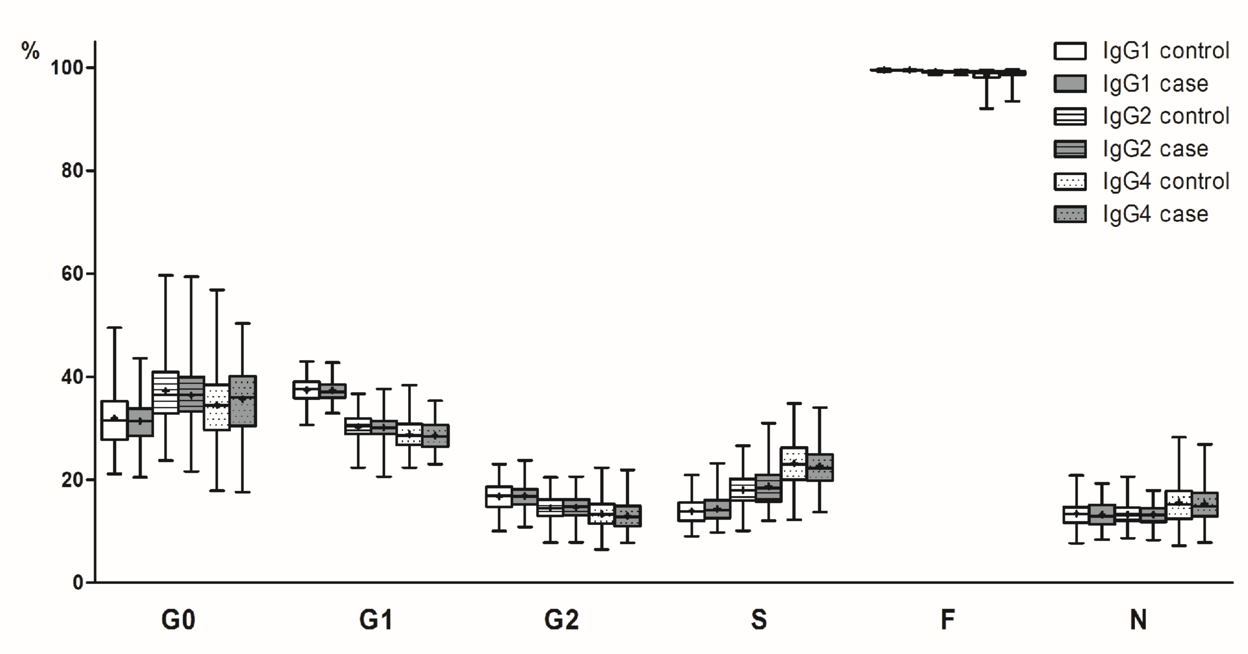

Supplemental Figure 8. No difference in subclass specific IgG glycosylation pattern in children with a high serum total IgE level and suffering from allergic rhinitis in the last 12 months (n=108) and control children (n=255) (based on ISAAC questionnaire). G0 = proportion of agalactosylated structures in total subclass glycans. G1 = proportion of monogalactosylated structures in total subclass glycans. G2 = proportion of digalactosylated structures in total subclass glycans. S = proportion of sialylated structures in total subclass glycans. F = proportion of fucosylated structures in total subclass glycans. N = proportion of structures with bisecting *N*-acetylglucosamine in total subclass glycans. Data are shown as box and whiskers plots. Each box represents the 25th to 75th percentiles. Lines inside the boxes represent the median. '+'s inside the boxes represent the mean. The whiskers represent the lowest and highest values.

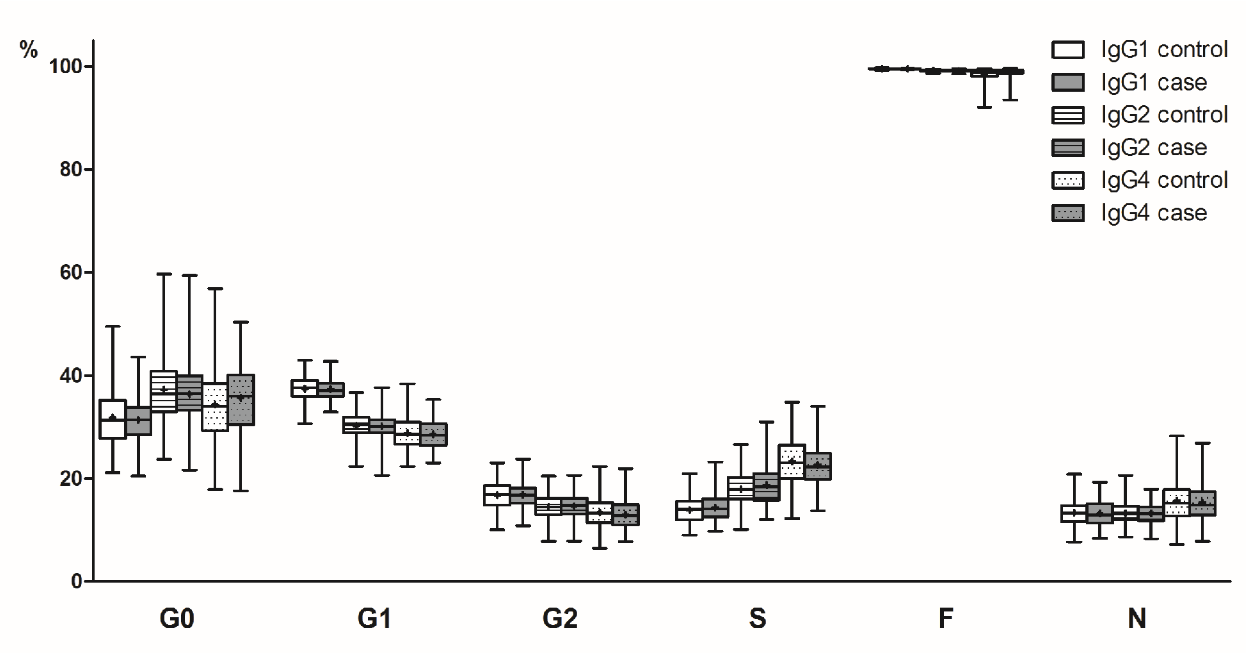

Supplemental Figure 9. No difference in subclass specific IgG glycosylation pattern in children with a high serum total IgE level, a positive skin prick test and suffering from allergic rhinitis in the last 12 months (n=108) and control children (n=218) (based on ISAAC questionnaire). G0 = proportion of agalactosylated structures in total subclass glycans. G1 = proportion of monogalactosylated structures in total subclass glycans. G2 = proportion of digalactosylated structures in total subclass glycans. S = proportion of sialylated structures in total subclass glycans. F = proportion of fucosylated structures in total subclass glycans. N = proportion of structures with bisecting *N*-acetylglucosamine in total subclass glycans. Data are shown as box and whiskers plots. Each box represents the 25th to 75th percentiles. Lines inside the boxes represent the median. '+'s inside the boxes represent the mean. The whiskers represent the lowest and highest values.

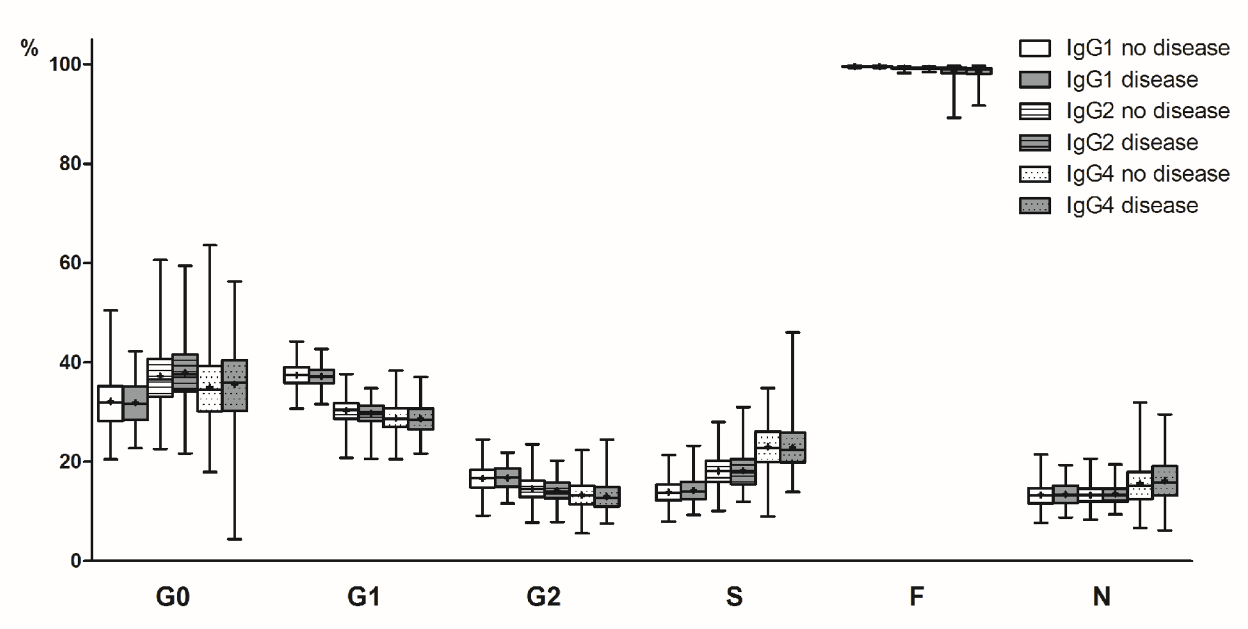

Supplemental Figure 10. No difference in subclass specific IgG glycosylation pattern in children suffering from allergic rhinoconjunctivitis in the last 12 months (n=145) and control children (n=425) (based on ISAAC questionnaire). G0 = proportion of agalactosylated structures in total subclass glycans. G1 = proportion of monogalactosylated structures in total subclass glycans. G2 = proportion of digalactosylated structures in total subclass glycans. S = proportion of sialylated structures in total subclass glycans. F = proportion of fucosylated structures in total subclass glycans. N = proportion of structures with bisecting *N*-acetylglucosamine in total subclass glycans. Data are shown as box and whiskers plots. Each box represents the 25th to 75th percentiles. Lines inside the boxes represent the median. '+'s inside the boxes represent the mean. The whiskers represent the lowest and highest values.

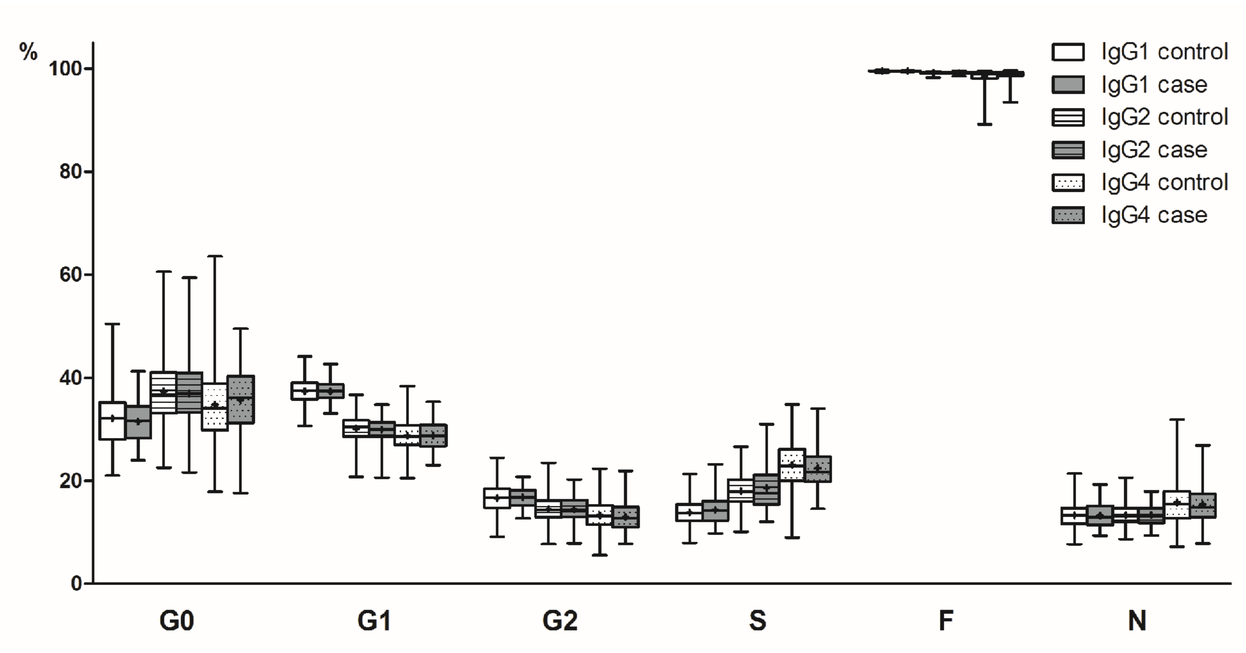

Supplemental Figure 11. No difference in subclass specific IgG glycosylation pattern in children with a high serum total IgE level and suffering from allergic rhinoconjunctivitis in the last 12 months (n=79) and control children (n=344) (based on ISAAC questionnaire). G0 = proportion of agalactosylated structures in total subclass glycans. G1 = proportion of monogalactosylated structures in total subclass glycans. G2 = proportion of digalactosylated structures in total subclass glycans. S = proportion of sialylated structures in total subclass glycans. F = proportion of fucosylated structures in total subclass glycans. N = proportion of structures with bisecting *N*-acetylglucosamine in total subclass glycans. Data are shown as box and whiskers plots. Each box represents the 25th to 75th percentiles. Lines inside the boxes represent the median. '+'s inside the boxes represent the mean. The whiskers represent the lowest and highest values.

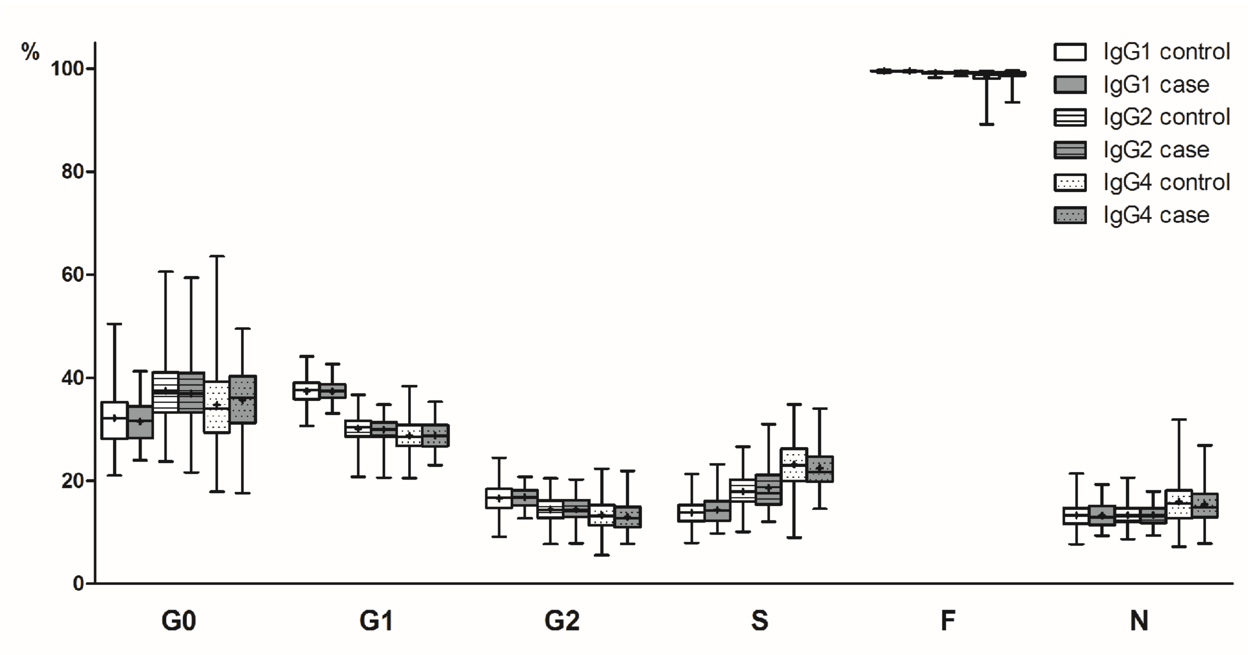

Supplemental Figure 12. No difference in subclass specific IgG glycosylation pattern in children with a high serum total IgE level, a positive skin prick test and suffering from allergic rhinoconjunctivitis in the last 12 months (n=79) and control children (n=295) (based on ISAAC questionnaire). G0 = proportion of agalactosylated structures in total subclass glycans. G1 = proportion of monogalactosylated structures in total subclass glycans. G2 = proportion of digalactosylated structures in total subclass glycans. S = proportion of sialylated structures in total subclass glycans. F = proportion of fucosylated structures in total subclass glycans. N = proportion of structures with bisecting *N*-acetylglucosamine in total subclass glycans. Data are shown as box and whiskers plots. Each box represents the 25th to 75th percentiles. Lines inside the boxes represent the median. '+'s inside the boxes represent the mean. The whiskers represent the lowest and highest values.

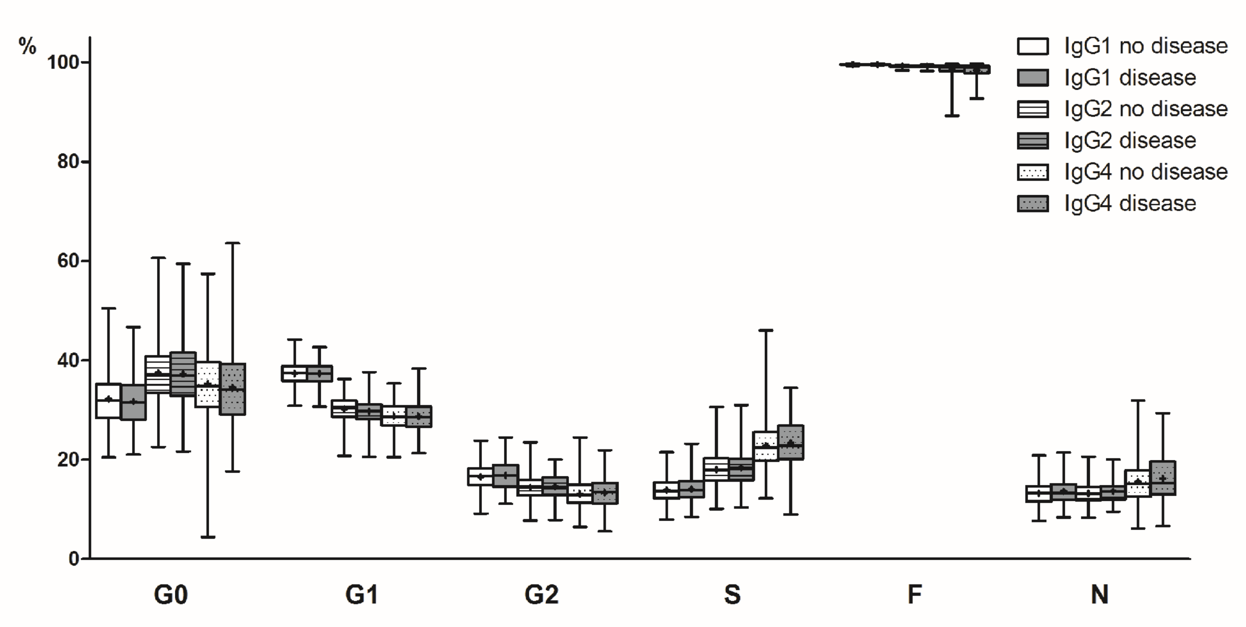

Supplemental Figure 13. No difference in subclass specific IgG glycosylation pattern in children suffering from atopic dermatitis in the last 12 months (n=151) and control children (n=419) (based on ISAAC questionnaire). G0 = proportion of agalactosylated structures in total subclass glycans. G1 = proportion of monogalactosylated structures in total subclass glycans. G2 = proportion of digalactosylated structures in total subclass glycans. S = proportion of sialylated structures in total subclass glycans. F = proportion of fucosylated structures in total subclass glycans. N = proportion of structures with bisecting *N*-acetylglucosamine in total subclass glycans. Data are shown as box and whiskers plots. Each box represents the 25th to 75th percentiles. Lines inside the boxes represent the median. '+'s inside the boxes represent the mean. The whiskers represent the lowest and highest values.

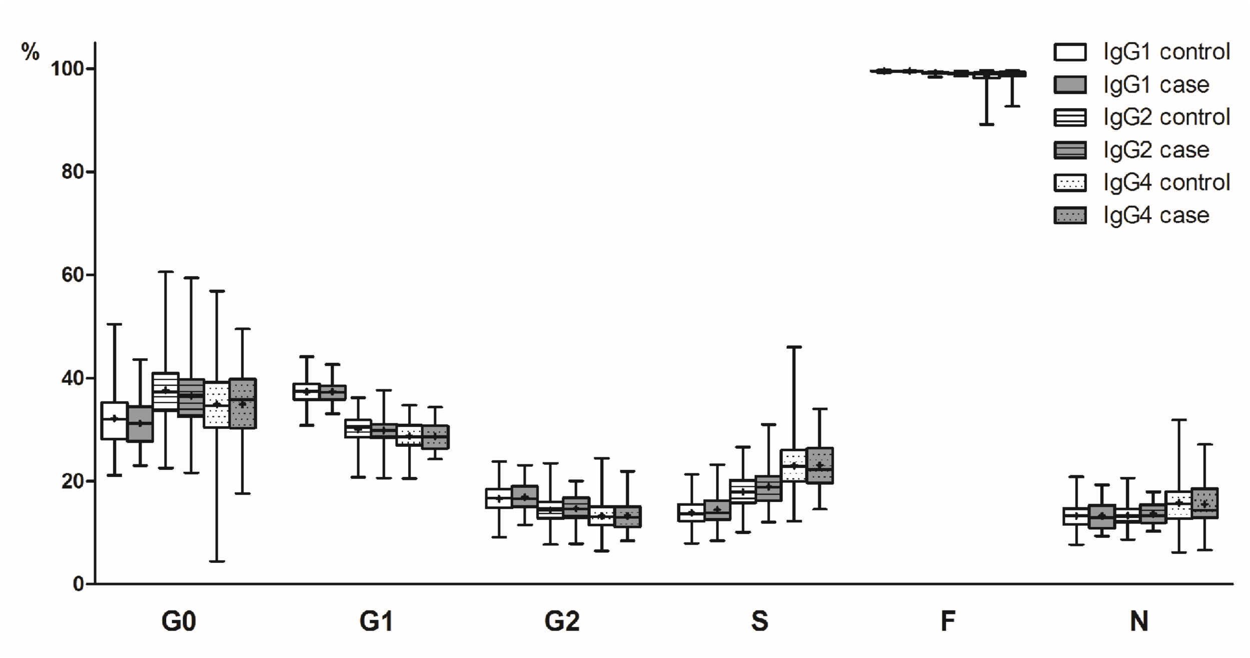

Supplemental Figure 14. No difference in subclass specific IgG glycosylation pattern in children with a high serum total IgE level and suffering from atopic dermatitis in the last 12 months (n=54) and control children (n=312) (based on ISAAC questionnaire). G0 = proportion of agalactosylated structures in total subclass glycans. G1 = proportion of monogalactosylated structures in total subclass glycans. G2 = proportion of digalactosylated structures in total subclass glycans. S = proportion of sialylated structures in total subclass glycans. F = proportion of fucosylated structures in total subclass glycans. N = proportion of structures with bisecting *N*-acetylglucosamine in total subclass glycans. Data are shown as box and whiskers plots. Each box represents the 25th to 75th percentiles. Lines inside the boxes represent the median. '+'s inside the boxes represent the mean. The whiskers represent the lowest and highest values.

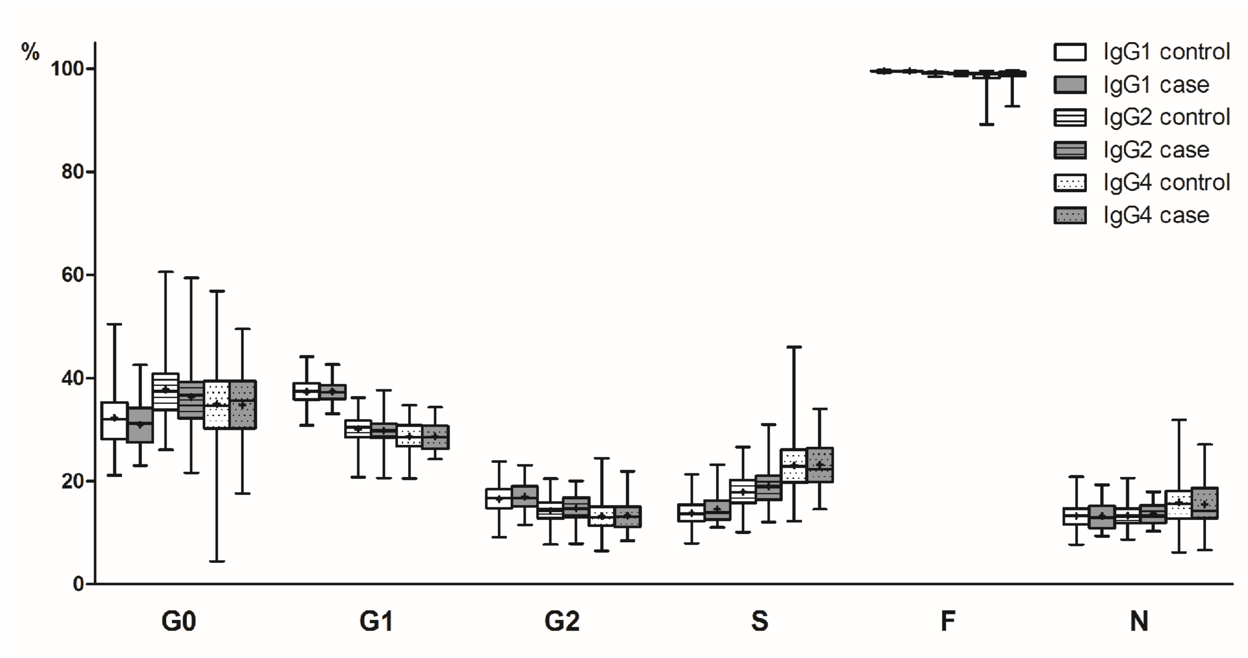

Supplemental Figure 15. No difference in subclass specific IgG glycosylation pattern in children with a high serum total IgE level, a positive skin prick test and suffering from atopic dermatitis in the last 12 months (n=53) and control children (n=270) (based on ISAAC questionnaire). G0 = proportion of agalactosylated structures in total subclass glycans. G1 = proportion of monogalactosylated structures in total subclass glycans. G2 = proportion of digalactosylated structures in total subclass glycans. S = proportion of sialylated structures in total subclass glycans. F = proportion of fucosylated structures in total subclass glycans. N = proportion of structures with bisecting *N*-acetylglucosamine in total subclass glycans. Data are shown as box and whiskers plots. Each box represents the 25th to 75th percentiles. Lines inside the boxes represent the median. '+'s inside the boxes represent the mean. The whiskers represent the lowest and highest values.

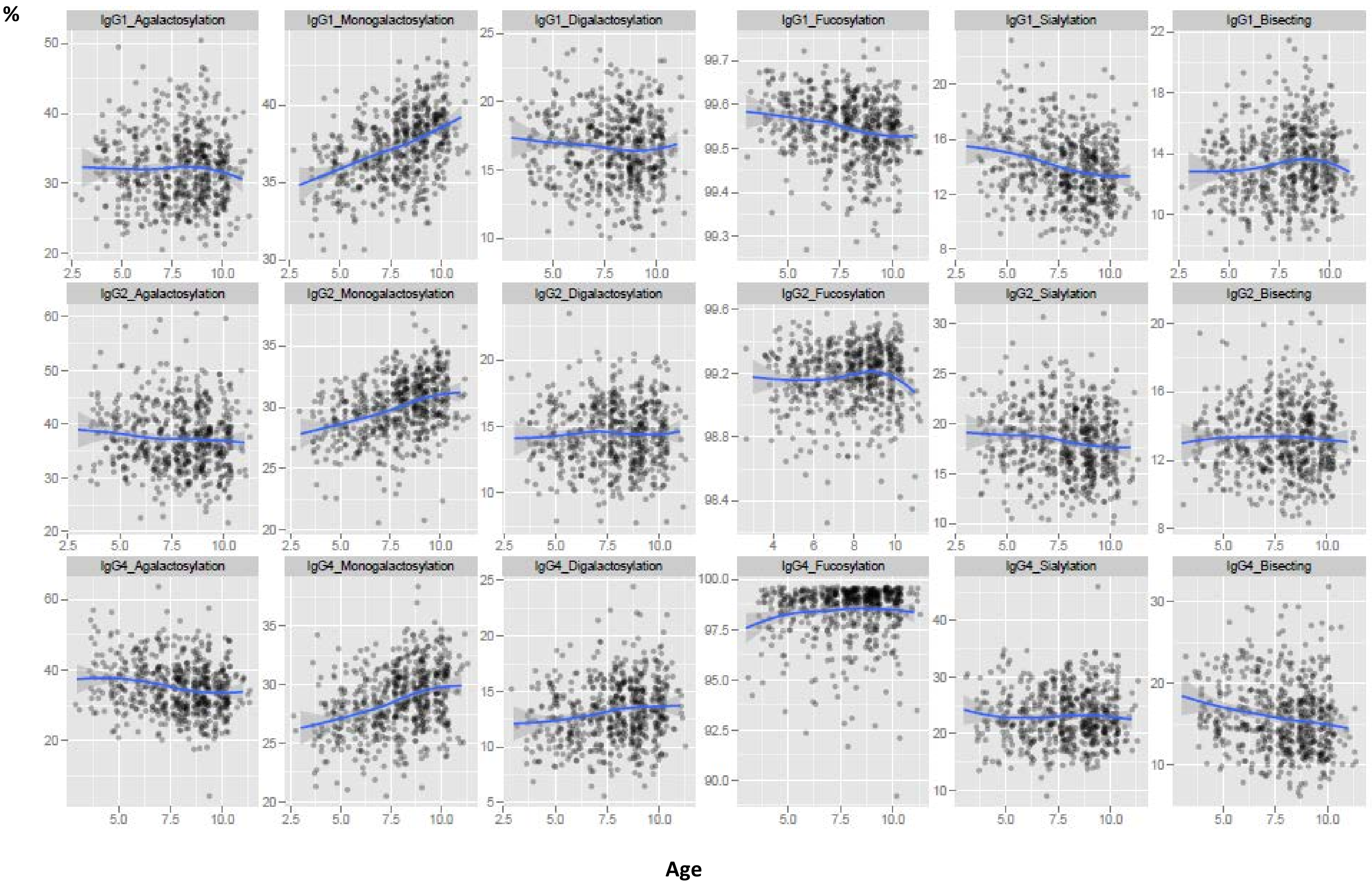

Supplemental Figure 16. Effects of age on IgG glycosylation in children. Percentages of particular sets of glycoforms in total subclass glycans are shown. Associations between age and glycopeptide measurements were examined in the Zagreb cohort using a regression model.

Supplemental Table 1. Aberdeen cohort - No difference in IgG glycosylation pattern (12 main glycan species and 6 derived traits) was found neither between children sensitized to at least one allergen and non-sensitized children, nor in respect to sensitization to any particular allergen, single allergen mean wheal diameter or positive wheal sum values.

Glycan	Trait	effect	SE	p	p adjusted
IgG4_G1FN	pos_cat	0.473	0.172	6.02E-03	9.63E-01
IgG2_G1NS	Wheal sum	0.021	0.008	1.27E-02	9.63E-01
IgG4_G1FS	pos_cat	-0.427	0.173	1.32E-02	9.63E-01
IgG1_G1NS	mm_cat	0.070	0.029	1.43E-02	9.63E-01
IgG1_G1NS	pos_cat	0.422	0.173	1.43E-02	9.63E-01
IgG2_G1NS	mm_pnut	0.074	0.031	1.57E-02	9.63E-01
IgG1_G1FNS	mm_dog	0.106	0.044	1.61E-02	9.63E-01
IgG1_G1FNS	pos_dog	0.504	0.213	1.78E-02	9.63E-01
IgG4_G2FNS	mm_cat	0.066	0.028	2.03E-02	9.63E-01
IgG2_G2FS	mm_hdm	-0.068	0.030	2.27E-02	9.63E-01
IgG4_G1NS	mm_egg	-0.192	0.086	2.55E-02	9.63E-01
IgG1_G1	mm_tg	0.051	0.023	2.57E-02	9.63E-01
IgG4_G1NS	pos_egg	-1.096	0.503	2.87E-02	9.63E-01
IgG1_F	mm_tg	0.049	0.023	2.89E-02	9.63E-01
IgG1_G1FS	mm_hdm	-0.065	0.030	2.99E-02	9.63E-01
IgG4_G0F	pos_egg	1.076	0.502	3.13E-02	9.63E-01
IgG2_G1S	pos_pnut	-0.762	0.358	3.23E-02	9.63E-01
IgG4_G1NS	mm_pnut	-0.066	0.031	3.31E-02	9.63E-01
IgG1_G1	pos_tg	0.286	0.135	3.34E-02	9.63E-01
IgG2_G2FN	mm_cat	0.060	0.028	3.36E-02	9.63E-01
IgG1_G1F	mm_tg	0.049	0.023	3.42E-02	9.63E-01
IgG4_G1FN	mm_cat	0.060	0.029	3.53E-02	9.63E-01
IgG1_G1FN	pos_hdm	0.310	0.149	3.66E-02	9.63E-01
IgG2_G1FN	mm_cat	0.058	0.028	3.82E-02	9.63E-01
IgG1_G1S	pos_hdm	-0.311	0.151	3.88E-02	9.63E-01
IgG2_G2FS	pos_hdm	-0.311	0.151	3.88E-02	9.63E-01
IgG2_G1FN	pos_cat	0.345	0.169	4.02E-02	9.63E-01
IgG1_F	Wheal sum	0.017	0.008	4.10E-02	9.63E-01
IgG4_G1FN	pos_hdm	0.308	0.152	4.12E-02	9.63E-01
IgG2_S	mm_hdm	-0.060	0.030	4.48E-02	9.63E-01
IgG2_G1S	mm_pnut	-0.062	0.031	4.50E-02	9.63E-01
IgG2_G1NS	mm_tg	0.045	0.023	4.89E-02	9.63E-01
IgG1_S	mm_egg	-0.167	0.086	5.09E-02	9.63E-01
IgG4_G0F	mm_egg	0.166	0.086	5.34E-02	9.63E-01
IgG4_G1NS	pos_pnut	-0.681	0.358	5.59E-02	9.63E-01
IgG1_G1FN	pos_cat	0.323	0.171	5.78E-02	9.63E-01
IgG1_S	pos_hdm	-0.283	0.151	5.88E-02	9.63E-01
IgG2_G1	pos_cat	0.322	0.172	5.95E-02	9.63E-01
IgG4_G1FS	pos_hdm	-0.282	0.152	6.18E-02	9.63E-01
IgG4_G1FNS	mm_tg	-0.042	0.023	6.28E-02	9.63E-01
IgG1_G1FS	pos_hdm	-0.278	0.150	6.30E-02	9.63E-01
IgG1_G1F	pos_tg	0.250	0.136	6.42E-02	9.63E-01
IgG1_G1	Sensitization	0.228	0.124	6.52E-02	9.63E-01
IgG1_G1S	mm_hdm	-0.055	0.030	6.53E-02	9.63E-01
IgG4_G0	pos_egg	0.916	0.501	6.58E-02	9.63E-01
IgG2_G2F	pos_hdm	-0.275	0.151	6.73E-02	9.63E-01
IgG4_G1NS	Wheal sum	-0.015	0.009	6.97E-02	9.63E-01
IgG2_G1S	mm_dog	-0.080	0.044	7.03E-02	9.63E-01
IgG4_S	pos_cat	-0.310	0.173	7.11E-02	9.63E-01

IgG2_G1FNS	pos_cat	0.309	0.173	7.23E-02	9.63E-01
IgG1_G2FS	mm_egg	-0.154	0.087	7.33E-02	9.63E-01
IgG2_G2FS	pos_cat	-0.308	0.174	7.44E-02	9.63E-01
IgG2_G1NS	pos_pnut	0.629	0.357	7.65E-02	9.63E-01
IgG2_G2	pos_hdm	-0.267	0.152	7.67E-02	9.63E-01
IgG1_S	mm_hdm	-0.053	0.030	7.68E-02	9.63E-01
IgG2_F	mm_cat	0.050	0.029	7.78E-02	9.63E-01
IgG1_G1S	mm_dog	-0.078	0.044	7.80E-02	9.63E-01
IgG4_S	pos_hdm	-0.263	0.151	7.90E-02	9.63E-01
IgG2_S	pos_hdm	-0.263	0.151	8.05E-02	9.63E-01
IgG1_F	pos_tg	0.232	0.134	8.14E-02	9.63E-01
IgG2_G1S	pos_dog	-0.369	0.214	8.29E-02	9.63E-01
IgG1_G0FN	pos_egg	0.868	0.505	8.40E-02	9.63E-01
IgG1_G0	mm_egg	0.148	0.086	8.40E-02	9.63E-01
IgG4_G1NS	pos_hdm	-0.260	0.151	8.40E-02	9.63E-01
IgG4_G1S	mm_cat	0.048	0.029	8.96E-02	9.63E-01
IgG2_N	pos_hdm	0.250	0.149	9.19E-02	9.63E-01
IgG1_F	pos_dog	0.353	0.212	9.30E-02	9.63E-01
IgG1_G1FN	mm_cat	0.047	0.028	9.42E-02	9.63E-01
IgG1_G2	mm_egg	-0.144	0.087	9.48E-02	9.63E-01
IgG1_G0F	mm_egg	0.143	0.086	9.56E-02	9.63E-01
IgG1_G1FN	mm_dog	0.071	0.044	1.02E-01	9.63E-01
IgG2_N	pos_cat	0.278	0.171	1.02E-01	9.63E-01
IgG2_G2F	mm_hdm	-0.049	0.030	1.06E-01	9.63E-01
IgG1_G2FNS	mm_cat	0.046	0.029	1.07E-01	9.63E-01
IgG2_G1NS	pos_cat	0.277	0.173	1.07E-01	9.63E-01
IgG4_G2FN	mm_cat	0.046	0.029	1.08E-01	9.63E-01
IgG1_G2F	mm_egg	-0.137	0.087	1.13E-01	9.63E-01
IgG4_G0	mm_egg	0.135	0.086	1.13E-01	9.63E-01
IgG2_G2	mm_hdm	-0.047	0.030	1.16E-01	9.63E-01
IgG2_G0	mm_hdm	0.047	0.030	1.17E-01	9.63E-01
IgG1_G1S	pos_cat	-0.271	0.174	1.17E-01	9.63E-01
IgG2_N	mm_cat	0.044	0.028	1.18E-01	9.63E-01
IgG1_G2	mm_pnut	-0.048	0.031	1.19E-01	9.63E-01
IgG2_G1FNS	mm_cat	0.044	0.029	1.21E-01	9.63E-01
IgG1_G1S	pos_tg	-0.209	0.136	1.22E-01	9.63E-01
IgG1_G2FNS	pos_cat	0.263	0.172	1.24E-01	9.63E-01
IgG2_G0	pos_hdm	0.231	0.151	1.24E-01	9.63E-01
IgG2_G1FS	mm_tg	0.035	0.023	1.25E-01	9.63E-01
IgG1_S	Sensitization	-0.188	0.124	1.26E-01	9.63E-01
IgG1_G0	pos_egg	0.765	0.505	1.27E-01	9.63E-01
IgG1_N	pos_cat	0.261	0.172	1.28E-01	9.63E-01
IgG1_S	pos_egg	-0.759	0.504	1.30E-01	9.63E-01
IgG1_G1FS	pos_cat	-0.259	0.173	1.30E-01	9.63E-01
IgG2_G2FN	pos_cat	0.257	0.171	1.30E-01	9.63E-01
IgG4_N	pos_cat	0.258	0.172	1.30E-01	9.63E-01
IgG1_S	pos_cat	-0.257	0.173	1.35E-01	9.63E-01
IgG1_S	pos_tg	-0.200	0.135	1.37E-01	9.63E-01
IgG1_G1S	pos_dog	-0.317	0.215	1.37E-01	9.63E-01
IgG2_G1NS	mm_cat	0.042	0.029	1.38E-01	9.63E-01

IgG1_G2F	mm_pnut	-0.046	0.031	1.40E-01	9.63E-01
IgG1_G1FN	mm_hdm	0.044	0.030	1.42E-01	9.63E-01
IgG2_G0F	mm_hdm	0.044	0.030	1.43E-01	9.63E-01
IgG2_G1NS	mm_dog	0.065	0.044	1.43E-01	9.63E-01
IgG4_G1FNS	pos_tg	-0.196	0.135	1.44E-01	9.63E-01
IgG1_F	Sensitization	0.178	0.123	1.44E-01	9.63E-01
IgG4_G0F	pos_cat	0.250	0.173	1.45E-01	9.63E-01
IgG4_G1FNS	Sensitization	-0.179	0.124	1.46E-01	9.63E-01
IgG4_G1FS	mm_hdm	-0.044	0.030	1.47E-01	9.63E-01
IgG2_G1FN	pos_hdm	0.213	0.148	1.48E-01	9.63E-01
IgG2_G1F	pos_cat	0.247	0.172	1.49E-01	9.63E-01
IgG4_G1FS	mm_pnut	0.045	0.031	1.51E-01	9.63E-01
IgG4_G1FN	Wheal sum	0.012	0.009	1.51E-01	9.63E-01
IgG4_F	Wheal sum	0.012	0.009	1.51E-01	9.63E-01
IgG4_G1S	mm_pnut	0.044	0.031	1.56E-01	9.63E-01
IgG1_G2FS	pos_egg	-0.713	0.507	1.57E-01	9.63E-01
IgG1_G1F	Sensitization	0.174	0.125	1.59E-01	9.63E-01
IgG4_G1FN	mm_hdm	0.042	0.030	1.60E-01	9.63E-01
IgG4_G1FNS	pos_cat	-0.241	0.173	1.61E-01	9.63E-01
IgG4_G2F	pos_egg	-0.691	0.497	1.61E-01	9.63E-01
IgG2_G1NS	Sensitization	0.172	0.124	1.62E-01	9.63E-01
IgG1_N	pos_hdm	0.209	0.151	1.63E-01	9.63E-01
IgG2_S	pos_cat	-0.241	0.174	1.63E-01	9.63E-01
IgG2_G1	mm_cat	0.040	0.029	1.63E-01	9.63E-01
IgG4_F	mm_pnut	0.043	0.031	1.66E-01	9.63E-01
IgG2_G1	pos_dog	0.293	0.213	1.66E-01	9.63E-01
IgG4_G0F	pos_hdm	0.207	0.151	1.67E-01	9.63E-01
IgG2_G0F	pos_hdm	0.208	0.152	1.67E-01	9.63E-01
IgG1_G1S	mm_tg	-0.032	0.023	1.68E-01	9.63E-01
IgG1_G2FNS	mm_dog	0.060	0.044	1.71E-01	9.63E-01
IgG1_G1S	Sensitization	-0.169	0.125	1.73E-01	9.63E-01
IgG2_G0FN	pos_hdm	0.204	0.151	1.74E-01	9.63E-01
IgG1_G0F	pos_egg	0.681	0.505	1.75E-01	9.63E-01
IgG1_G0FN	mm_egg	0.117	0.087	1.75E-01	9.63E-01
IgG1_G2FN	mm_egg	-0.115	0.086	1.77E-01	9.63E-01
IgG1_F	mm_cat	0.038	0.028	1.80E-01	9.63E-01
IgG4_S	mm_hdm	-0.040	0.030	1.81E-01	9.63E-01
IgG1_G2	pos_pnut	-0.476	0.359	1.82E-01	9.63E-01
IgG2_F	mm_dog	0.059	0.044	1.82E-01	9.63E-01
IgG1_G2	pos_egg	-0.668	0.506	1.83E-01	9.63E-01
IgG2_F	Wheal sum	0.011	0.009	1.84E-01	9.63E-01
IgG1_F	pos_pnut	0.467	0.355	1.86E-01	9.63E-01
IgG4_N	pos_hdm	0.197	0.150	1.86E-01	9.63E-01
IgG4_G2	pos_egg	-0.654	0.498	1.86E-01	9.63E-01
IgG1_G2FN	pos_pnut	-0.465	0.355	1.87E-01	9.63E-01
IgG2_G1FNS	pos_tg	0.178	0.136	1.87E-01	9.63E-01
IgG4_G1FNS	Wheal sum	-0.011	0.009	1.91E-01	9.63E-01
IgG4_G2FNS	pos_cat	0.223	0.173	1.94E-01	9.63E-01
IgG1_G2F	pos_egg	-0.649	0.506	1.96E-01	9.63E-01
IgG4_F	mm_egg	0.111	0.087	1.97E-01	9.63E-01

IgG2_G1NS	pos_tg	0.173	0.135	1.98E-01	9.63E-01
IgG4_G2FN	pos_pnut	-0.457	0.360	2.00E-01	9.63E-01
IgG1_G1	Wheal sum	0.011	0.009	2.01E-01	9.63E-01
IgG1_N	mm_dog	0.056	0.044	2.02E-01	9.63E-01
IgG4_F	mm_dog	0.056	0.044	2.03E-01	9.63E-01
IgG2_F	mm_egg	0.109	0.087	2.05E-01	9.63E-01
IgG1_G2F	pos_pnut	-0.449	0.359	2.08E-01	9.63E-01
IgG4_G1NS	mm_tg	-0.029	0.023	2.09E-01	9.63E-01
IgG2_G1FNS	Wheal sum	0.011	0.009	2.10E-01	9.63E-01
IgG4_G1F	mm_dog	-0.055	0.044	2.11E-01	9.63E-01
IgG2_F	pos_egg	0.627	0.506	2.11E-01	9.63E-01
IgG4_G0	pos_hdm	0.186	0.151	2.12E-01	9.63E-01
IgG1_G2FN	mm_cat	0.035	0.028	2.13E-01	9.63E-01
IgG1_N	mm_cat	0.035	0.029	2.15E-01	9.63E-01
IgG4_G2F	pos_pnut	-0.434	0.353	2.16E-01	9.63E-01
IgG4_G2F	mm_egg	-0.105	0.085	2.16E-01	9.63E-01
IgG4_G2	pos_pnut	-0.435	0.354	2.16E-01	9.63E-01
IgG4_G1S	pos_cat	0.212	0.173	2.16E-01	9.63E-01
IgG4_G1NS	pos_cat	-0.212	0.174	2.18E-01	9.63E-01
IgG1_G2FN	mm_pnut	-0.037	0.031	2.21E-01	9.63E-01
IgG4_G1S	Wheal sum	0.010	0.009	2.21E-01	9.63E-01
IgG1_G2FNS	pos_dog	0.259	0.213	2.21E-01	9.63E-01
IgG1_F	mm_dog	0.053	0.044	2.23E-01	9.63E-01
IgG1_G1S	Wheal sum	-0.010	0.009	2.23E-01	9.63E-01
IgG4_F	Sensitization	0.149	0.124	2.28E-01	9.63E-01
IgG1_G1	pos_hdm	0.182	0.152	2.28E-01	9.63E-01
IgG1_G1FS	Sensitization	-0.148	0.124	2.28E-01	9.63E-01
IgG4_S	pos_egg	-0.603	0.505	2.28E-01	9.63E-01
IgG4_G0	pos_cat	0.206	0.172	2.29E-01	9.63E-01
IgG2_N	pos_egg	0.596	0.500	2.30E-01	9.63E-01
IgG4_G2FNS	mm_dog	0.053	0.044	2.31E-01	9.63E-01
IgG2_F	mm_tg	0.027	0.023	2.32E-01	9.63E-01
IgG1_N	pos_egg	0.594	0.503	2.34E-01	9.63E-01
IgG4_G2FN	mm_tg	-0.027	0.023	2.35E-01	9.63E-01
IgG1_S	mm_tg	-0.027	0.023	2.35E-01	9.63E-01
IgG4_G2	mm_egg	-0.101	0.085	2.36E-01	9.63E-01
IgG2_G1NS	pos_dog	0.248	0.214	2.42E-01	9.79E-01
IgG4_G1FS	pos_pnut	0.417	0.361	2.44E-01	9.79E-01
IgG2_F	pos_cat	0.200	0.173	2.45E-01	9.79E-01
IgG2_G1S	Wheal sum	-0.010	0.009	2.47E-01	9.79E-01
IgG4_G1NS	Sensitization	-0.143	0.124	2.47E-01	9.79E-01
IgG4_F	pos_hdm	0.174	0.151	2.47E-01	9.79E-01
IgG2_G0FN	pos_egg	0.574	0.505	2.52E-01	9.79E-01
IgG4_S	mm_egg	-0.098	0.087	2.53E-01	9.79E-01
IgG1_G1FS	mm_egg	-0.098	0.086	2.53E-01	9.79E-01
IgG4_G1NS	mm_hdm	-0.034	0.030	2.53E-01	9.79E-01
IgG1_G1FNS	pos_cat	0.197	0.174	2.54E-01	9.79E-01
IgG4_G1FS	mm_cat	-0.032	0.029	2.59E-01	9.79E-01
IgG1_G1FNS	mm_egg	-0.098	0.087	2.59E-01	9.79E-01
IgG4_G1NS	pos_tg	-0.151	0.136	2.61E-01	9.79E-01

IgG2_G1	mm_dog	0.049	0.044	2.62E-01	9.79E-01
IgG2_G1NS	pos_hdm	0.168	0.151	2.62E-01	9.79E-01
IgG2_F	pos_tg	0.151	0.136	2.63E-01	9.79E-01
IgG4_G1S	pos_hdm	-0.167	0.151	2.66E-01	9.79E-01
IgG1_G1FNS	mm_cat	0.032	0.029	2.66E-01	9.79E-01
IgG4_G2FN	pos_cat	0.191	0.174	2.68E-01	9.79E-01
IgG2_G1F	pos_dog	0.234	0.213	2.69E-01	9.79E-01
IgG2_G1NS	mm_egg	0.095	0.087	2.71E-01	9.79E-01
IgG4_F	pos_pnut	0.392	0.359	2.71E-01	9.79E-01
IgG2_N	mm_hdm	0.033	0.030	2.71E-01	9.79E-01
IgG4_G0F	pos_pnut	0.391	0.359	2.72E-01	9.79E-01
IgG2_G1FN	mm_dog	0.047	0.044	2.79E-01	9.82E-01
IgG2_G0FN	mm_pnut	0.033	0.031	2.80E-01	9.82E-01
IgG4_F	pos_cat	0.185	0.173	2.83E-01	9.82E-01
IgG4_G1FNS	mm_dog	-0.047	0.044	2.87E-01	9.82E-01
IgG4_F	pos_egg	0.526	0.505	2.94E-01	9.82E-01
IgG2_G2FS	Wheal sum	-0.009	0.009	2.94E-01	9.82E-01
IgG1_G0FN	pos_cat	0.181	0.174	2.95E-01	9.82E-01
IgG4_G2FS	pos_egg	-0.516	0.500	2.98E-01	9.82E-01
IgG4_G1FS	Sensitization	-0.129	0.125	3.00E-01	9.82E-01
IgG1_G2	mm_cat	0.029	0.029	3.04E-01	9.82E-01
IgG2_G2FNS	mm_cat	0.029	0.029	3.05E-01	9.82E-01
IgG2_G1NS	mm_hdm	0.031	0.030	3.07E-01	9.82E-01
IgG4_G2	mm_cat	0.029	0.028	3.08E-01	9.82E-01
IgG4_G1FN	Sensitization	0.126	0.125	3.08E-01	9.82E-01
IgG4_G1FN	pos_tg	0.138	0.136	3.09E-01	9.82E-01
IgG4_N	mm_cat	0.029	0.029	3.10E-01	9.82E-01
IgG4_S	mm_pnut	0.031	0.031	3.11E-01	9.82E-01
IgG2_G1F	pos_egg	-0.501	0.503	3.16E-01	9.82E-01
IgG4_G2FS	mm_egg	-0.085	0.086	3.18E-01	9.82E-01
IgG4_G1S	pos_pnut	0.352	0.358	3.21E-01	9.82E-01
IgG2_G0FN	mm_hdm	0.029	0.030	3.26E-01	9.82E-01
IgG1_G1	mm_hdm	0.029	0.030	3.28E-01	9.82E-01
IgG1_G2FS	pos_hdm	-0.147	0.152	3.29E-01	9.82E-01
IgG4_G1S	pos_egg	0.486	0.505	3.31E-01	9.82E-01
IgG4_N	mm_tg	-0.022	0.023	3.40E-01	9.82E-01
IgG1_G1NS	Wheal sum	0.008	0.009	3.41E-01	9.82E-01
IgG2_G2F	mm_pnut	-0.030	0.031	3.41E-01	9.82E-01
IgG2_G1FS	pos_tg	0.128	0.135	3.41E-01	9.82E-01
IgG4_G0F	Sensitization	0.117	0.124	3.41E-01	9.82E-01
IgG1_F	mm_pnut	0.029	0.031	3.41E-01	9.82E-01
IgG4_F	pos_tg	0.128	0.136	3.41E-01	9.82E-01
IgG2_G1FS	mm_hdm	-0.028	0.030	3.44E-01	9.82E-01
IgG2_F	pos_dog	0.201	0.214	3.44E-01	9.82E-01
IgG1_G1FN	pos_dog	0.198	0.212	3.46E-01	9.82E-01
IgG4_G1FNS	pos_pnut	-0.335	0.359	3.47E-01	9.82E-01
IgG1_G2FS	pos_cat	-0.162	0.174	3.48E-01	9.82E-01
IgG1_F	pos_cat	0.160	0.172	3.49E-01	9.82E-01
IgG4_G2FS	pos_cat	-0.159	0.171	3.51E-01	9.82E-01
IgG4_G2FNS	Wheal sum	0.008	0.009	3.52E-01	9.82E-01

IgG1_G1FN	pos_egg	0.462	0.501	3.52E-01	9.82E-01
IgG1_N	mm_hdm	0.028	0.030	3.54E-01	9.82E-01
IgG2_G0	pos_egg	0.464	0.506	3.56E-01	9.82E-01
IgG2_G1FNS	mm_tg	0.021	0.023	3.57E-01	9.82E-01
IgG1_G0	pos_pnut	0.329	0.359	3.57E-01	9.82E-01
IgG1_G1NS	mm_hdm	0.028	0.030	3.57E-01	9.82E-01
IgG4_G0F	mm_hdm	0.027	0.030	3.60E-01	9.82E-01
IgG1_G1	pos_dog	0.195	0.215	3.60E-01	9.82E-01
IgG2_G1F	mm_cat	0.026	0.029	3.61E-01	9.82E-01
IgG2_F	mm_hdm	0.027	0.030	3.62E-01	9.82E-01
IgG2_G0	mm_egg	0.079	0.087	3.62E-01	9.82E-01
IgG2_G1FN	pos_dog	0.188	0.210	3.66E-01	9.82E-01
IgG1_G1FN	Wheal sum	0.008	0.009	3.70E-01	9.82E-01
IgG4_G1FS	pos_tg	-0.121	0.136	3.72E-01	9.82E-01
IgG2_N	mm_pnut	0.027	0.031	3.76E-01	9.82E-01
IgG2_G2F	mm_egg	-0.076	0.087	3.77E-01	9.82E-01
IgG4_G1S	mm_egg	0.076	0.087	3.78E-01	9.82E-01
IgG1_G2F	mm_cat	0.025	0.029	3.79E-01	9.82E-01
IgG2_N	Wheal sum	0.007	0.008	3.80E-01	9.82E-01
IgG2_G2	pos_pnut	-0.313	0.361	3.82E-01	9.82E-01
IgG2_N	mm_egg	0.074	0.086	3.82E-01	9.82E-01
IgG1_G2FS	pos_tg	-0.118	0.136	3.84E-01	9.82E-01
IgG4_G2FN	mm_pnut	-0.027	0.031	3.85E-01	9.82E-01
IgG1_G0F	mm_pnut	0.027	0.031	3.87E-01	9.82E-01
IgG1_G0	mm_pnut	0.027	0.031	3.87E-01	9.82E-01
IgG2_G1S	pos_cat	0.149	0.174	3.88E-01	9.82E-01
IgG4_G1FN	mm_dog	0.038	0.045	3.88E-01	9.82E-01
IgG1_G0F	mm_dog	-0.038	0.044	3.90E-01	9.82E-01
IgG2_G1FN	Sensitization	0.104	0.122	3.91E-01	9.82E-01
IgG4_G1F	pos_egg	-0.429	0.507	3.93E-01	9.82E-01
IgG1_G1FN	Sensitization	0.104	0.123	3.94E-01	9.82E-01
IgG4_G1S	mm_dog	0.038	0.044	3.94E-01	9.82E-01
IgG2_G1FNS	mm_egg	0.073	0.087	3.94E-01	9.82E-01
IgG4_G1S	pos_tg	0.114	0.135	3.95E-01	9.82E-01
IgG2_F	Sensitization	0.105	0.124	3.97E-01	9.82E-01
IgG1_G0F	pos_pnut	0.302	0.359	3.97E-01	9.82E-01
IgG2_G1F	mm_dog	0.037	0.044	3.97E-01	9.82E-01
IgG4_N	mm_hdm	0.025	0.030	3.98E-01	9.82E-01
IgG4_G2FN	pos_hdm	0.127	0.152	3.98E-01	9.82E-01
IgG4_G1FNS	pos_dog	-0.179	0.214	3.99E-01	9.82E-01
IgG2_G2	mm_pnut	-0.026	0.031	3.99E-01	9.82E-01
IgG2_G1	pos_egg	-0.421	0.504	4.00E-01	9.82E-01
IgG4_F	mm_cat	0.024	0.029	4.00E-01	9.82E-01
IgG2_G2FNS	mm_pnut	0.026	0.031	4.01E-01	9.82E-01
IgG4_G2F	mm_cat	0.024	0.028	4.02E-01	9.82E-01
IgG4_G2FS	mm_hdm	-0.025	0.030	4.03E-01	9.82E-01
IgG4_G1FNS	mm_cat	-0.024	0.029	4.05E-01	9.82E-01
IgG4_N	mm_dog	0.036	0.044	4.10E-01	9.82E-01
IgG1_S	Wheal sum	-0.007	0.009	4.11E-01	9.82E-01
IgG1_G1FNS	Wheal sum	0.007	0.009	4.12E-01	9.82E-01

IgG2_G2FN	mm_tg	-0.019	0.023	4.12E-01	9.82E-01
IgG2_G2FNS	pos_cat	0.141	0.174	4.12E-01	9.82E-01
IgG2_G2F	pos_pnut	-0.291	0.360	4.16E-01	9.82E-01
IgG2_G1FN	mm_hdm	0.024	0.030	4.17E-01	9.82E-01
IgG2_G1FN	Wheal sum	0.007	0.008	4.18E-01	9.82E-01
IgG4_F	pos_dog	0.172	0.214	4.18E-01	9.82E-01
IgG2_G2	mm_egg	-0.070	0.087	4.19E-01	9.82E-01
IgG2_G0FN	mm_egg	0.069	0.087	4.20E-01	9.82E-01
IgG4_G0F	Wheal sum	0.007	0.009	4.21E-01	9.82E-01
IgG2_G0F	mm_egg	0.069	0.087	4.24E-01	9.82E-01
IgG1_F	pos_hdm	0.119	0.150	4.24E-01	9.82E-01
IgG1_G0FN	mm_tg	-0.018	0.023	4.28E-01	9.82E-01
IgG4_G0FN	pos_hdm	0.117	0.149	4.29E-01	9.82E-01
IgG2_G1S	mm_egg	-0.068	0.087	4.30E-01	9.82E-01
IgG1_G1FS	pos_egg	-0.394	0.505	4.31E-01	9.82E-01
IgG4_G0F	mm_tg	0.018	0.023	4.32E-01	9.82E-01
IgG1_G1FS	Wheal sum	-0.007	0.009	4.32E-01	9.82E-01
IgG4_G0	mm_hdm	0.023	0.030	4.33E-01	9.82E-01
IgG2_G2F	pos_tg	0.106	0.136	4.33E-01	9.82E-01
IgG1_G2FNS	Wheal sum	0.007	0.009	4.34E-01	9.82E-01
IgG1_G0FN	pos_pnut	0.279	0.360	4.36E-01	9.82E-01
IgG2_G2FS	mm_cat	-0.022	0.029	4.36E-01	9.82E-01
IgG1_G2FN	pos_egg	-0.386	0.500	4.37E-01	9.82E-01
IgG4_G1FNS	mm_pnut	-0.024	0.031	4.38E-01	9.82E-01
IgG1_G0F	mm_cat	-0.022	0.029	4.39E-01	9.82E-01
IgG1_G2FS	mm_hdm	-0.023	0.030	4.40E-01	9.82E-01
IgG4_G1FS	Wheal sum	-0.007	0.009	4.41E-01	9.82E-01
IgG4_F	mm_tg	0.018	0.023	4.41E-01	9.82E-01
IgG1_G1FNS	Sensitization	0.095	0.125	4.42E-01	9.82E-01
IgG4_G2FS	mm_pnut	0.023	0.031	4.42E-01	9.82E-01
IgG4_G1	mm_dog	-0.034	0.045	4.43E-01	9.82E-01
IgG2_G0FN	pos_cat	0.132	0.173	4.44E-01	9.82E-01
IgG1_G1	pos_cat	0.131	0.174	4.48E-01	9.82E-01
IgG2_G1FNS	pos_egg	0.380	0.507	4.50E-01	9.82E-01
IgG1_G1S	pos_pnut	-0.270	0.361	4.50E-01	9.82E-01
IgG1_G1FS	mm_pnut	0.023	0.031	4.52E-01	9.82E-01
IgG1_G2FN	mm_dog	0.033	0.044	4.52E-01	9.82E-01
IgG1_G2FNS	mm_egg	-0.064	0.086	4.53E-01	9.82E-01
IgG4_G0	Sensitization	0.092	0.124	4.55E-01	9.82E-01
IgG2_G0FN	pos_pnut	0.265	0.359	4.56E-01	9.82E-01
IgG1_G1S	pos_egg	-0.375	0.508	4.57E-01	9.82E-01
IgG4_G0	pos_pnut	0.263	0.357	4.58E-01	9.82E-01
IgG4_G0FN	mm_pnut	-0.023	0.031	4.59E-01	9.82E-01
IgG1_G1FNS	pos_tg	0.100	0.136	4.60E-01	9.82E-01
IgG2_G1FS	pos_pnut	0.262	0.358	4.61E-01	9.82E-01
IgG1_N	mm_egg	0.063	0.086	4.62E-01	9.82E-01
IgG2_G2FS	mm_egg	-0.063	0.087	4.63E-01	9.82E-01
IgG4_G1FS	pos_egg	-0.370	0.509	4.63E-01	9.82E-01
IgG1_G1FS	pos_tg	-0.098	0.135	4.64E-01	9.82E-01
IgG1_G1F	Wheal sum	0.006	0.009	4.65E-01	9.82E-01

IgG2_F	mm_pnut	-0.023	0.031	4.66E-01	9.82E-01
IgG4_S	Sensitization	-0.090	0.124	4.67E-01	9.82E-01
IgG4_G1FN	mm_egg	0.063	0.087	4.68E-01	9.82E-01
IgG2_S	mm_tg	0.017	0.023	4.70E-01	9.82E-01
IgG4_G2FN	mm_hdm	0.022	0.030	4.70E-01	9.82E-01
IgG2_G2F	pos_egg	-0.363	0.508	4.71E-01	9.82E-01
IgG1_G2FS	mm_tg	-0.017	0.023	4.72E-01	9.82E-01
IgG2_G0F	pos_egg	0.361	0.507	4.73E-01	9.82E-01
IgG4_G1FN	pos_egg	0.362	0.509	4.73E-01	9.82E-01
IgG2_G2FNS	Wheal sum	0.006	0.009	4.74E-01	9.82E-01
IgG1_G1S	mm_egg	-0.062	0.087	4.75E-01	9.82E-01
IgG4_G2FS	pos_hdm	-0.106	0.150	4.77E-01	9.82E-01
IgG2_G0	mm_pnut	0.022	0.031	4.79E-01	9.82E-01
IgG2_G2FN	pos_pnut	-0.248	0.355	4.82E-01	9.82E-01
IgG4_G0F	pos_tg	0.093	0.136	4.89E-01	9.82E-01
IgG2_G2F	mm_tg	0.016	0.023	4.91E-01	9.82E-01
IgG4_N	mm_pnut	-0.021	0.031	4.91E-01	9.82E-01
IgG4_G2FN	mm_dog	0.030	0.045	4.92E-01	9.82E-01
IgG2_G2	pos_tg	0.092	0.136	4.95E-01	9.82E-01
IgG2_G1FN	pos_egg	0.334	0.496	4.97E-01	9.82E-01
IgG4_G1	pos_egg	-0.341	0.507	4.97E-01	9.82E-01
IgG1_G1S	mm_pnut	0.021	0.031	4.98E-01	9.82E-01
IgG2_G1FNS	Sensitization	0.083	0.125	5.00E-01	9.82E-01
IgG4_G0FN	mm_tg	-0.015	0.023	5.00E-01	9.82E-01
IgG1_G2FN	mm_hdm	0.020	0.030	5.01E-01	9.82E-01
IgG2_G2FNS	mm_egg	0.058	0.087	5.01E-01	9.82E-01
IgG2_F	pos_hdm	0.101	0.152	5.01E-01	9.82E-01
IgG1_F	mm_hdm	0.020	0.030	5.02E-01	9.82E-01
IgG1_G1FS	pos_dog	0.142	0.214	5.04E-01	9.82E-01
IgG2_G2FNS	pos_egg	0.335	0.507	5.05E-01	9.82E-01
IgG2_G2FNS	pos_dog	-0.142	0.215	5.06E-01	9.82E-01
IgG2_G2FNS	pos_tg	0.089	0.136	5.10E-01	9.82E-01
IgG2_G1	Wheal sum	0.006	0.009	5.16E-01	9.82E-01
IgG1_G2FS	Sensitization	-0.080	0.125	5.18E-01	9.82E-01
IgG4_G2FNS	mm_pnut	0.020	0.031	5.18E-01	9.82E-01
IgG1_G2FNS	Sensitization	-0.079	0.124	5.18E-01	9.82E-01
IgG1_F	mm_egg	0.055	0.086	5.21E-01	9.82E-01
IgG2_G2FNS	pos_hdm	0.096	0.152	5.24E-01	9.82E-01
IgG2_G1NS	pos_egg	0.319	0.506	5.24E-01	9.82E-01
IgG1_G2F	mm_tg	0.015	0.023	5.25E-01	9.82E-01
IgG1_G0FN	mm_dog	0.028	0.045	5.29E-01	9.82E-01
IgG1_G1FNS	pos_egg	-0.317	0.508	5.29E-01	9.82E-01
IgG1_S	mm_pnut	0.019	0.031	5.31E-01	9.82E-01
IgG4_N	pos_pnut	-0.218	0.356	5.37E-01	9.82E-01
IgG2_G1FNS	pos_hdm	0.093	0.152	5.38E-01	9.82E-01
IgG2_G1FS	pos_hdm	-0.092	0.151	5.39E-01	9.82E-01
IgG4_G2	mm_pnut	-0.019	0.031	5.39E-01	9.82E-01
IgG2_G0FN	mm_tg	-0.014	0.023	5.41E-01	9.82E-01
IgG1_N	mm_tg	-0.014	0.023	5.42E-01	9.82E-01
IgG2_G1	Sensitization	0.075	0.124	5.42E-01	9.82E-01

IgG4_G1S	mm_tg	0.014	0.023	5.44E-01	9.82E-01
IgG2_G2	pos_egg	-0.305	0.508	5.46E-01	9.82E-01
IgG4_G2F	mm_pnut	-0.018	0.031	5.50E-01	9.82E-01
IgG2_G2FS	Sensitization	-0.074	0.125	5.51E-01	9.82E-01
IgG2_S	Wheal sum	-0.005	0.009	5.54E-01	9.82E-01
IgG1_G2F	pos_tg	0.080	0.136	5.54E-01	9.82E-01
IgG1_G1FNS	mm_tg	0.014	0.023	5.58E-01	9.82E-01
IgG2_G2FNS	Sensitization	0.072	0.125	5.60E-01	9.82E-01
IgG2_G1S	Sensitization	0.072	0.125	5.60E-01	9.82E-01
IgG1_G2	mm_tg	0.013	0.023	5.60E-01	9.82E-01
IgG1_G0FN	pos_hdm	0.088	0.152	5.61E-01	9.82E-01
IgG2_N	pos_pnut	0.204	0.356	5.63E-01	9.82E-01
IgG1_G0F	pos_dog	-0.122	0.215	5.65E-01	9.82E-01
IgG2_G1S	pos_egg	-0.289	0.508	5.67E-01	9.82E-01
IgG1_G1NS	mm_tg	-0.013	0.023	5.69E-01	9.82E-01
IgG1_G2	Sensitization	0.070	0.125	5.69E-01	9.82E-01
IgG4_G1FS	mm_tg	-0.013	0.023	5.70E-01	9.82E-01
IgG1_G1FS	pos_pnut	0.202	0.358	5.70E-01	9.82E-01
IgG1_G2	pos_tg	0.077	0.136	5.71E-01	9.82E-01
IgG4_G0FN	pos_cat	0.096	0.171	5.71E-01	9.82E-01
IgG2_N	mm_dog	0.025	0.044	5.72E-01	9.82E-01
IgG4_G0FN	pos_dog	0.118	0.211	5.72E-01	9.82E-01
IgG1_G2	mm_dog	0.025	0.045	5.74E-01	9.82E-01
IgG4_G1	pos_cat	0.097	0.174	5.75E-01	9.82E-01
IgG2_G1FN	mm_egg	0.047	0.085	5.76E-01	9.82E-01
IgG2_G0F	mm_pnut	0.017	0.031	5.79E-01	9.82E-01
IgG4_G1	pos_hdm	0.084	0.152	5.79E-01	9.82E-01
IgG2_N	mm_tg	-0.013	0.023	5.81E-01	9.82E-01
IgG4_G1F	pos_cat	-0.095	0.174	5.82E-01	9.82E-01
IgG1_G1FNS	pos_hdm	0.083	0.152	5.83E-01	9.82E-01
IgG2_G1FS	Wheal sum	0.005	0.009	5.85E-01	9.82E-01
IgG2_G1FN	mm_pnut	-0.017	0.031	5.86E-01	9.82E-01
IgG4_G1FN	pos_dog	0.116	0.216	5.88E-01	9.82E-01
IgG1_G2F	Sensitization	0.066	0.125	5.92E-01	9.82E-01
IgG4_G1S	Sensitization	0.066	0.124	5.92E-01	9.82E-01
IgG4_F	mm_hdm	0.016	0.030	5.92E-01	9.82E-01
IgG1_G0FN	pos_tg	-0.072	0.136	5.93E-01	9.82E-01
IgG2_G0	Wheal sum	0.005	0.009	5.94E-01	9.82E-01
IgG2_G1	pos_pnut	-0.189	0.358	5.94E-01	9.82E-01
IgG2_G2	mm_tg	0.012	0.023	5.94E-01	9.82E-01
IgG4_G0FN	mm_dog	0.023	0.044	5.97E-01	9.82E-01
IgG4_G1FS	mm_egg	-0.046	0.087	5.97E-01	9.82E-01
IgG2_G2F	Wheal sum	-0.005	0.009	5.97E-01	9.82E-01
IgG1_G2FN	pos_cat	0.090	0.172	5.98E-01	9.82E-01
IgG1_G1NS	pos_tg	0.071	0.137	5.99E-01	9.82E-01
IgG1_G2FNS	pos_egg	-0.262	0.505	6.00E-01	9.82E-01
IgG1_G1FN	mm_pnut	-0.016	0.031	6.00E-01	9.82E-01
IgG2_G1S	pos_tg	0.071	0.136	6.01E-01	9.82E-01
IgG2_G1FNS	mm_pnut	0.016	0.031	6.01E-01	9.82E-01
IgG2_N	Sensitization	0.064	0.123	6.03E-01	9.82E-01

IgG1_G0	mm_cat	-0.015	0.029	6.04E-01	9.82E-01
IgG4_G2F	pos_tg	0.069	0.134	6.05E-01	9.82E-01
IgG2_G0	pos_pnut	0.184	0.360	6.05E-01	9.82E-01
IgG2_S	mm_egg	-0.045	0.087	6.06E-01	9.82E-01
IgG1_G1F	pos_egg	-0.260	0.508	6.07E-01	9.82E-01
IgG1_G0	mm_dog	-0.023	0.045	6.08E-01	9.82E-01
IgG1_G0FN	mm_cat	0.015	0.029	6.10E-01	9.82E-01
IgG2_F	pos_pnut	-0.181	0.360	6.12E-01	9.82E-01
IgG1_G1FN	mm_egg	0.043	0.086	6.12E-01	9.82E-01
IgG4_G1	pos_tg	0.068	0.136	6.13E-01	9.82E-01
IgG2_G0FN	mm_cat	0.014	0.029	6.13E-01	9.82E-01
IgG2_G1	pos_tg	0.067	0.135	6.16E-01	9.82E-01
IgG2_G1FS	pos_cat	-0.086	0.173	6.18E-01	9.82E-01
IgG2_G2	Wheal sum	-0.004	0.009	6.19E-01	9.82E-01
IgG1_G1NS	mm_dog	0.022	0.045	6.20E-01	9.82E-01
IgG2_G2FS	pos_egg	-0.249	0.508	6.21E-01	9.82E-01
IgG1_G0	pos_dog	-0.105	0.215	6.23E-01	9.82E-01
IgG2_G1FN	pos_tg	0.065	0.133	6.24E-01	9.82E-01
IgG2_G0FN	Wheal sum	0.004	0.009	6.25E-01	9.82E-01
IgG2_G2FS	pos_dog	-0.104	0.215	6.26E-01	9.82E-01
IgG1_G1	mm_dog	0.022	0.045	6.27E-01	9.82E-01
IgG4_G1F	pos_dog	-0.103	0.215	6.28E-01	9.82E-01
IgG1_G2FN	pos_hdm	0.072	0.150	6.29E-01	9.82E-01
IgG4_G0	Wheal sum	0.004	0.009	6.29E-01	9.82E-01
IgG2_G1F	Wheal sum	0.004	0.009	6.30E-01	9.82E-01
IgG4_N	pos_dog	0.101	0.213	6.31E-01	9.82E-01
IgG4_G1	Wheal sum	0.004	0.009	6.35E-01	9.82E-01
IgG1_G1NS	pos_pnut	-0.170	0.362	6.35E-01	9.82E-01
IgG4_G1	mm_cat	0.014	0.029	6.37E-01	9.82E-01
IgG1_G1FS	mm_dog	0.021	0.044	6.39E-01	9.82E-01
IgG2_G2FS	mm_pnut	-0.015	0.031	6.39E-01	9.82E-01
IgG2_G1FN	pos_pnut	-0.163	0.352	6.41E-01	9.82E-01
IgG1_G1FS	mm_cat	-0.013	0.029	6.41E-01	9.82E-01
IgG4_G2FN	mm_egg	-0.040	0.087	6.42E-01	9.82E-01
IgG2_G1FS	mm_pnut	0.014	0.031	6.43E-01	9.82E-01
IgG1_G1FS	mm_tg	-0.011	0.023	6.44E-01	9.82E-01
IgG2_G1	mm_pnut	-0.014	0.031	6.46E-01	9.82E-01
IgG4_G0	pos_tg	0.062	0.135	6.46E-01	9.82E-01
IgG1_N	Wheal sum	0.004	0.009	6.46E-01	9.82E-01
IgG4_G2F	mm_tg	0.010	0.023	6.47E-01	9.82E-01
IgG4_G2F	Sensitization	0.056	0.123	6.48E-01	9.82E-01
IgG2_S	mm_cat	-0.013	0.029	6.50E-01	9.82E-01
IgG2_G0FN	pos_tg	-0.060	0.136	6.54E-01	9.82E-01
IgG4_G0FN	mm_hdm	0.013	0.030	6.54E-01	9.82E-01
IgG1_G1	mm_cat	0.013	0.029	6.56E-01	9.82E-01
IgG2_G1FNS	pos_dog	-0.093	0.215	6.61E-01	9.82E-01
IgG2_G1F	pos_hdm	-0.066	0.151	6.62E-01	9.82E-01
IgG2_G1F	mm_egg	-0.037	0.086	6.62E-01	9.82E-01
IgG4_G2	pos_tg	0.058	0.134	6.63E-01	9.82E-01
IgG2_G1F	Sensitization	0.054	0.124	6.63E-01	9.82E-01

IgG2_G0F	Wheal sum	0.004	0.009	6.64E-01	9.82E-01
IgG1_G0	pos_hdm	0.065	0.152	6.67E-01	9.82E-01
IgG4_G1	mm_hdm	0.013	0.030	6.67E-01	9.82E-01
IgG2_G1F	pos_pnut	-0.152	0.358	6.69E-01	9.82E-01
IgG2_S	pos_tg	0.057	0.136	6.71E-01	9.82E-01
IgG2_S	pos_dog	-0.091	0.215	6.71E-01	9.82E-01
IgG4_S	pos_tg	-0.057	0.136	6.71E-01	9.82E-01
IgG2_G2FNS	mm_tg	0.010	0.023	6.72E-01	9.82E-01
IgG4_G1NS	mm_cat	-0.012	0.029	6.75E-01	9.82E-01
IgG2_G0F	pos_pnut	0.149	0.360	6.76E-01	9.82E-01
IgG1_G0FN	pos_dog	-0.089	0.215	6.76E-01	9.82E-01
IgG1_G2F	mm_dog	0.019	0.045	6.76E-01	9.82E-01
IgG2_G1FNS	mm_hdm	0.013	0.030	6.77E-01	9.82E-01
IgG4_G0	mm_tg	0.009	0.023	6.81E-01	9.82E-01
IgG4_N	pos_egg	0.205	0.502	6.81E-01	9.82E-01
IgG1_G2FS	mm_pnut	0.013	0.031	6.81E-01	9.82E-01
IgG4_G2FNS	pos_pnut	-0.144	0.359	6.86E-01	9.82E-01
IgG2_G0F	mm_cat	-0.012	0.029	6.87E-01	9.82E-01
IgG1_G2FNS	pos_hdm	0.060	0.151	6.89E-01	9.82E-01
IgG2_G1F	pos_tg	0.053	0.135	6.90E-01	9.82E-01
IgG4_S	Wheal sum	-0.003	0.009	6.92E-01	9.82E-01
IgG2_G2FNS	mm_dog	-0.018	0.045	6.92E-01	9.82E-01
IgG1_G2FNS	mm_tg	-0.009	0.023	6.93E-01	9.82E-01
IgG1_G2FS	mm_dog	0.017	0.045	6.94E-01	9.82E-01
IgG4_G2FNS	mm_egg	-0.034	0.087	6.96E-01	9.82E-01
IgG4_G1FN	mm_pnut	0.012	0.031	6.98E-01	9.82E-01
IgG1_G2	pos_dog	0.083	0.215	6.99E-01	9.82E-01
IgG4_G2FNS	mm_tg	-0.009	0.023	6.99E-01	9.82E-01
IgG1_N	pos_dog	0.082	0.214	7.00E-01	9.82E-01
IgG1_G1F	pos_dog	0.082	0.215	7.02E-01	9.82E-01
IgG4_G2FN	pos_dog	0.080	0.215	7.08E-01	9.82E-01
IgG1_G1FNS	mm_hdm	0.011	0.030	7.10E-01	9.82E-01
IgG4_G2	Sensitization	0.045	0.123	7.12E-01	9.82E-01
IgG2_G1S	mm_cat	0.011	0.029	7.13E-01	9.82E-01
IgG1_G2F	pos_hdm	-0.055	0.152	7.15E-01	9.82E-01
IgG2_G1FS	Sensitization	0.045	0.124	7.15E-01	9.82E-01
IgG1_G1NS	pos_hdm	0.055	0.153	7.17E-01	9.82E-01
IgG2_G2FN	mm_egg	-0.031	0.086	7.18E-01	9.82E-01
IgG4_G2FS	mm_tg	0.008	0.023	7.18E-01	9.82E-01
IgG2_G2FS	mm_tg	0.008	0.023	7.18E-01	9.82E-01
IgG4_G1F	mm_cat	-0.010	0.029	7.18E-01	9.82E-01
IgG2_G2FN	Sensitization	0.044	0.123	7.20E-01	9.82E-01
IgG2_G2FN	mm_pnut	0.011	0.031	7.20E-01	9.82E-01
IgG1_S	mm_dog	0.016	0.044	7.24E-01	9.82E-01
IgG4_G2FN	pos_egg	-0.177	0.508	7.25E-01	9.82E-01
IgG1_G1F	mm_cat	-0.010	0.029	7.27E-01	9.82E-01
IgG1_G1	mm_egg	0.030	0.087	7.30E-01	9.82E-01
IgG1_G0	pos_cat	0.059	0.174	7.31E-01	9.82E-01
IgG4_G0F	pos_dog	0.073	0.214	7.31E-01	9.82E-01
IgG4_N	Sensitization	0.042	0.123	7.32E-01	9.82E-01

IgG1_G0	mm_hdm	0.010	0.030	7.33E-01	9.82E-01
IgG4_G0	pos_dog	0.072	0.213	7.33E-01	9.82E-01
IgG1_G1F	mm_dog	-0.015	0.045	7.34E-01	9.82E-01
IgG2_G1S	mm_hdm	-0.010	0.030	7.35E-01	9.82E-01
IgG2_G2F	pos_cat	-0.058	0.174	7.39E-01	9.82E-01
IgG2_S	Sensitization	-0.041	0.125	7.40E-01	9.82E-01
IgG2_S	pos_egg	-0.165	0.508	7.43E-01	9.82E-01
IgG2_G0	pos_tg	-0.044	0.136	7.45E-01	9.82E-01
IgG4_G1F	mm_tg	0.007	0.023	7.46E-01	9.82E-01
IgG2_G0F	pos_tg	-0.043	0.136	7.48E-01	9.82E-01
IgG2_G2	mm_cat	0.009	0.029	7.50E-01	9.82E-01
IgG2_G1F	mm_pnut	-0.010	0.031	7.52E-01	9.82E-01
IgG1_G2F	pos_dog	0.067	0.215	7.52E-01	9.82E-01
IgG4_G0FN	pos_pnut	-0.110	0.354	7.54E-01	9.82E-01
IgG2_G0	pos_cat	0.054	0.174	7.55E-01	9.82E-01
IgG2_G2FN	Wheal sum	0.003	0.008	7.56E-01	9.82E-01
IgG4_G2F	pos_hdm	-0.046	0.149	7.56E-01	9.82E-01
IgG1_G2	pos_cat	0.053	0.174	7.57E-01	9.82E-01
IgG4_G2FNS	pos_hdm	0.046	0.151	7.59E-01	9.82E-01
IgG1_G0FN	mm_hdm	0.009	0.030	7.60E-01	9.82E-01
IgG4_G2FNS	pos_egg	-0.153	0.505	7.60E-01	9.82E-01
IgG4_G2	pos_hdm	-0.045	0.150	7.61E-01	9.82E-01
IgG4_G1F	pos_hdm	-0.046	0.152	7.62E-01	9.82E-01
IgG4_G0FN	pos_egg	0.149	0.498	7.62E-01	9.82E-01
IgG4_G1FN	pos_pnut	0.107	0.362	7.66E-01	9.82E-01
IgG2_G1FS	mm_cat	0.008	0.029	7.67E-01	9.82E-01
IgG2_G1	mm_egg	-0.025	0.086	7.68E-01	9.82E-01
IgG1_G1F	mm_hdm	0.009	0.030	7.73E-01	9.82E-01
IgG1_G1F	pos_hdm	0.044	0.152	7.73E-01	9.82E-01
IgG1_G1FN	pos_tg	0.038	0.135	7.76E-01	9.82E-01
IgG4_G2	pos_cat	0.048	0.171	7.77E-01	9.82E-01
IgG2_G0	mm_tg	-0.007	0.023	7.78E-01	9.82E-01
IgG1_N	mm_pnut	-0.009	0.031	7.79E-01	9.82E-01
IgG1_G2FN	pos_dog	0.059	0.212	7.79E-01	9.82E-01
IgG1_N	Sensitization	0.034	0.124	7.80E-01	9.82E-01
IgG4_G2	mm_dog	0.012	0.044	7.83E-01	9.82E-01
IgG4_G2	mm_tg	0.006	0.023	7.85E-01	9.82E-01
IgG4_G2	pos_dog	0.057	0.212	7.87E-01	9.82E-01
IgG1_G2FS	Wheal sum	-0.002	0.009	7.87E-01	9.82E-01
IgG2_G1F	mm_tg	0.006	0.023	7.87E-01	9.82E-01
IgG1_G1NS	pos_dog	-0.057	0.216	7.88E-01	9.82E-01
IgG1_F	pos_egg	0.133	0.502	7.89E-01	9.82E-01
IgG1_G1S	mm_cat	-0.008	0.029	7.89E-01	9.82E-01
IgG4_S	mm_dog	0.012	0.044	7.90E-01	9.82E-01
IgG2_G0FN	pos_dog	-0.056	0.214	7.91E-01	9.82E-01
IgG4_G1FNS	pos_hdm	-0.040	0.152	7.91E-01	9.82E-01
IgG2_G1FS	mm_dog	0.012	0.044	7.92E-01	9.82E-01
IgG1_G2FN	mm_tg	-0.006	0.023	7.92E-01	9.82E-01
IgG4_G0	mm_cat	-0.007	0.029	7.94E-01	9.82E-01
IgG4_G2	Wheal sum	0.002	0.008	7.98E-01	9.82E-01

IgG4_G1FNS	mm_hdm	-0.008	0.030	7.98E-01	9.82E-01
IgG4_G2FS	mm_cat	0.007	0.028	8.01E-01	9.82E-01
IgG1_G0FN	mm_pnut	0.008	0.031	8.02E-01	9.82E-01
IgG2_G2F	Sensitization	0.031	0.125	8.03E-01	9.82E-01
IgG4_G2F	Wheal sum	0.002	0.008	8.06E-01	9.82E-01
IgG4_G0F	mm_pnut	0.008	0.031	8.07E-01	9.82E-01
IgG4_G1F	Sensitization	-0.030	0.125	8.09E-01	9.82E-01
IgG4_S	mm_tg	-0.006	0.023	8.10E-01	9.82E-01
IgG2_G0	mm_cat	-0.007	0.029	8.10E-01	9.82E-01
IgG1_G0	pos_tg	-0.032	0.136	8.11E-01	9.82E-01
IgG4_S	pos_dog	-0.051	0.214	8.12E-01	9.82E-01
IgG4_G2FS	Wheal sum	0.002	0.008	8.14E-01	9.82E-01
IgG4_G2FN	Sensitization	0.029	0.125	8.14E-01	9.82E-01
IgG1_G2FNS	mm_hdm	0.007	0.030	8.16E-01	9.82E-01
IgG2_G0F	mm_tg	-0.005	0.023	8.17E-01	9.82E-01
IgG2_G2FNS	pos_pnut	0.081	0.360	8.21E-01	9.82E-01
IgG2_G0FN	mm_dog	0.010	0.044	8.22E-01	9.82E-01
IgG4_G1F	pos_pnut	-0.080	0.360	8.22E-01	9.82E-01
IgG1_G2	pos_hdm	-0.034	0.152	8.22E-01	9.82E-01
IgG4_N	Wheal sum	0.002	0.009	8.23E-01	9.82E-01
IgG2_G2	Sensitization	0.028	0.125	8.24E-01	9.82E-01
IgG1_G2FS	pos_dog	0.047	0.215	8.25E-01	9.82E-01
IgG1_G1FN	mm_tg	-0.005	0.023	8.25E-01	9.82E-01
IgG1_G1	pos_pnut	0.078	0.361	8.27E-01	9.82E-01
IgG2_S	pos_pnut	0.077	0.361	8.30E-01	9.82E-01
IgG4_G1	pos_dog	-0.046	0.215	8.30E-01	9.82E-01
IgG4_G1F	mm_hdm	-0.006	0.030	8.31E-01	9.82E-01
IgG1_G0	mm_tg	-0.005	0.023	8.34E-01	9.82E-01
IgG1_G0F	pos_tg	-0.028	0.136	8.37E-01	9.82E-01
IgG2_G1FNS	pos_pnut	0.073	0.360	8.38E-01	9.82E-01
IgG4_G1	pos_pnut	-0.073	0.360	8.38E-01	9.82E-01
IgG2_G2FS	mm_dog	-0.009	0.045	8.38E-01	9.82E-01
IgG4_G1S	mm_hdm	-0.006	0.030	8.41E-01	9.82E-01
IgG4_G0FN	Wheal sum	-0.002	0.008	8.41E-01	9.82E-01
IgG4_G2FS	pos_tg	0.027	0.134	8.41E-01	9.82E-01
IgG1_S	pos_dog	0.043	0.214	8.41E-01	9.82E-01
IgG2_G2FN	mm_dog	0.009	0.044	8.43E-01	9.82E-01
IgG4_G2FNS	pos_tg	0.027	0.135	8.43E-01	9.82E-01
IgG2_G0F	mm_dog	-0.009	0.045	8.45E-01	9.82E-01
IgG1_N	pos_pnut	0.069	0.358	8.46E-01	9.82E-01
IgG1_G0F	Sensitization	-0.024	0.125	8.47E-01	9.82E-01
IgG1_G2F	pos_cat	0.033	0.174	8.47E-01	9.82E-01
IgG1_G1	mm_pnut	-0.006	0.031	8.47E-01	9.82E-01
IgG4_G2F	pos_dog	0.040	0.211	8.47E-01	9.82E-01
IgG2_G2F	mm_dog	0.009	0.045	8.48E-01	9.82E-01
IgG2_G1	mm_tg	0.004	0.023	8.49E-01	9.82E-01
IgG4_G2FS	mm_dog	0.008	0.044	8.50E-01	9.82E-01
IgG1_N	pos_tg	-0.025	0.135	8.51E-01	9.82E-01
IgG2_G1S	mm_tg	-0.004	0.023	8.52E-01	9.82E-01
IgG4_G0FN	pos_tg	-0.025	0.134	8.52E-01	9.82E-01

IgG2_G2FNS	mm_hdm	0.006	0.030	8.55E-01	9.82E-01
IgG4_G1FNS	mm_egg	-0.016	0.087	8.57E-01	9.82E-01
IgG1_G2FNS	pos_pnut	0.064	0.358	8.57E-01	9.82E-01
IgG4_G1	mm_tg	0.004	0.023	8.57E-01	9.82E-01
IgG2_G2FS	pos_tg	0.024	0.136	8.58E-01	9.82E-01
IgG1_G2FS	mm_cat	0.005	0.029	8.59E-01	9.82E-01
IgG1_G1	pos_egg	-0.088	0.508	8.61E-01	9.82E-01
IgG4_G2F	mm_hdm	-0.005	0.030	8.64E-01	9.82E-01
IgG4_G1S	pos_dog	0.036	0.214	8.64E-01	9.82E-01
IgG1_G1FNS	mm_pnut	-0.005	0.031	8.66E-01	9.82E-01
IgG1_G2FN	Sensitization	0.020	0.123	8.69E-01	9.82E-01
IgG4_G1FN	mm_tg	-0.004	0.023	8.71E-01	9.82E-01
IgG4_G2F	pos_cat	0.028	0.171	8.71E-01	9.82E-01
IgG1_G0F	pos_hdm	0.024	0.152	8.72E-01	9.82E-01
IgG2_G2	pos_cat	-0.028	0.175	8.72E-01	9.82E-01
IgG2_G0F	pos_cat	0.027	0.174	8.74E-01	9.82E-01
IgG4_G2FN	Wheal sum	0.001	0.009	8.74E-01	9.82E-01
IgG4_G1F	mm_egg	-0.013	0.087	8.76E-01	9.82E-01
IgG4_G2	mm_hdm	-0.005	0.030	8.76E-01	9.82E-01
IgG4_G0FN	Sensitization	0.019	0.123	8.78E-01	9.82E-01
IgG1_G2FN	pos_tg	-0.020	0.134	8.79E-01	9.82E-01
IgG2_S	mm_dog	-0.007	0.045	8.79E-01	9.82E-01
IgG4_S	pos_pnut	0.054	0.359	8.80E-01	9.82E-01
IgG1_G1NS	Sensitization	0.019	0.125	8.81E-01	9.82E-01
IgG2_G2FN	mm_hdm	-0.004	0.030	8.81E-01	9.82E-01
IgG4_G1NS	pos_dog	0.032	0.215	8.82E-01	9.82E-01
IgG4_G0	mm_dog	0.006	0.044	8.83E-01	9.82E-01
IgG4_G2FNS	pos_dog	0.030	0.214	8.87E-01	9.82E-01
IgG4_N	mm_egg	0.012	0.086	8.88E-01	9.82E-01
IgG1_G1F	pos_cat	-0.024	0.174	8.88E-01	9.82E-01
IgG2_G0FN	Sensitization	0.017	0.124	8.88E-01	9.82E-01
IgG4_G2F	mm_dog	0.006	0.044	8.90E-01	9.82E-01
IgG2_G2F	pos_dog	0.029	0.215	8.91E-01	9.82E-01
IgG1_G2FS	pos_pnut	-0.049	0.361	8.91E-01	9.82E-01
IgG4_G1NS	mm_dog	-0.006	0.045	8.92E-01	9.82E-01
IgG4_S	mm_cat	-0.004	0.029	8.93E-01	9.82E-01
IgG2_G2	mm_dog	0.006	0.045	8.94E-01	9.82E-01
IgG2_G1	mm_hdm	0.004	0.030	8.94E-01	9.82E-01
IgG4_G0FN	mm_cat	-0.004	0.028	8.96E-01	9.82E-01
IgG1_G0F	mm_hdm	0.004	0.030	8.98E-01	9.82E-01
IgG2_G1	pos_hdm	-0.019	0.151	8.99E-01	9.82E-01
IgG1_G0	Wheal sum	0.001	0.009	9.00E-01	9.82E-01
IgG2_G2F	mm_cat	0.004	0.029	9.02E-01	9.82E-01
IgG1_G0F	pos_cat	0.021	0.174	9.03E-01	9.82E-01
IgG4_G0F	mm_cat	-0.003	0.029	9.05E-01	9.82E-01
IgG4_G2FS	Sensitization	0.015	0.123	9.05E-01	9.82E-01
IgG2_S	mm_pnut	-0.004	0.031	9.06E-01	9.82E-01
IgG1_G1F	mm_egg	0.010	0.087	9.07E-01	9.82E-01
IgG4_G2FS	pos_pnut	-0.041	0.355	9.08E-01	9.82E-01
IgG4_G2FS	pos_dog	-0.024	0.212	9.08E-01	9.82E-01

IgG1_G2	mm_hdm	0.003	0.030	9.10E-01	9.82E-01
IgG4_G0F	mm_dog	0.005	0.044	9.10E-01	9.82E-01
IgG1_G0F	mm_tg	-0.002	0.023	9.22E-01	9.90E-01
IgG4_G1	Sensitization	0.012	0.125	9.23E-01	9.90E-01
IgG4_G2FN	pos_tg	-0.013	0.136	9.23E-01	9.90E-01
IgG1_S	mm_cat	-0.003	0.029	9.23E-01	9.90E-01
IgG2_G1FNS	mm_dog	-0.004	0.045	9.27E-01	9.92E-01
IgG1_G1NS	mm_egg	-0.007	0.087	9.32E-01	9.92E-01
IgG1_S	pos_pnut	0.029	0.359	9.35E-01	9.92E-01
IgG4_G1F	pos_tg	0.011	0.136	9.37E-01	9.92E-01
IgG2_N	pos_tg	-0.010	0.134	9.38E-01	9.92E-01
IgG4_G1FNS	pos_egg	0.039	0.506	9.39E-01	9.92E-01
IgG2_G1FN	mm_tg	-0.002	0.023	9.40E-01	9.92E-01
IgG1_G2FNS	mm_pnut	-0.002	0.031	9.41E-01	9.92E-01
IgG1_G2	Wheal sum	0.001	0.009	9.44E-01	9.92E-01
IgG2_G2FN	pos_tg	-0.009	0.134	9.44E-01	9.92E-01
IgG4_N	pos_tg	0.008	0.135	9.51E-01	9.92E-01
IgG2_G2FN	pos_hdm	0.009	0.150	9.51E-01	9.92E-01
IgG4_G0	mm_pnut	-0.002	0.031	9.52E-01	9.92E-01
IgG4_G1	mm_egg	0.005	0.087	9.54E-01	9.92E-01
IgG2_G0F	pos_dog	-0.012	0.215	9.55E-01	9.92E-01
IgG1_G1F	pos_pnut	0.020	0.361	9.55E-01	9.92E-01
IgG4_G1F	Wheal sum	-0.001	0.009	9.55E-01	9.92E-01
IgG2_G0	mm_dog	-0.002	0.045	9.57E-01	9.92E-01
IgG1_G0	Sensitization	-0.006	0.125	9.59E-01	9.92E-01
IgG2_G0	Sensitization	0.006	0.125	9.59E-01	9.92E-01
IgG1_G0FN	Sensitization	-0.006	0.125	9.60E-01	9.92E-01
IgG2_G1F	mm_hdm	-0.001	0.030	9.61E-01	9.92E-01
IgG1_G1FN	pos_pnut	-0.017	0.356	9.62E-01	9.92E-01
IgG2_G0	pos_dog	-0.010	0.215	9.62E-01	9.92E-01
IgG1_G2FNS	pos_tg	-0.007	0.135	9.62E-01	9.92E-01
IgG1_G1F	mm_pnut	-0.002	0.031	9.63E-01	9.92E-01
IgG1_G2F	Wheal sum	0.000	0.009	9.63E-01	9.92E-01
IgG1_G1NS	mm_pnut	0.001	0.031	9.63E-01	9.92E-01
IgG4_G2FNS	mm_hdm	0.001	0.030	9.66E-01	9.92E-01
IgG1_G0FN	Wheal sum	0.000	0.009	9.68E-01	9.92E-01
IgG4_G1	mm_pnut	0.001	0.031	9.68E-01	9.92E-01
IgG2_G2	pos_dog	0.008	0.215	9.69E-01	9.92E-01
IgG2_G2FS	pos_pnut	-0.014	0.361	9.69E-01	9.92E-01
IgG2_G1S	pos_hdm	-0.006	0.152	9.70E-01	9.92E-01
IgG2_G2FN	pos_egg	-0.018	0.501	9.71E-01	9.92E-01
IgG2_G0F	Sensitization	-0.004	0.125	9.74E-01	9.92E-01
IgG4_G1FS	mm_dog	0.001	0.045	9.75E-01	9.92E-01
IgG2_G1FS	pos_dog	-0.007	0.214	9.75E-01	9.92E-01
IgG2_G1FS	mm_egg	0.002	0.087	9.79E-01	9.94E-01
IgG4_G0FN	mm_egg	-0.002	0.085	9.83E-01	9.94E-01
IgG1_G2FN	Wheal sum	0.000	0.009	9.83E-01	9.94E-01
IgG2_G2FN	pos_dog	-0.004	0.212	9.84E-01	9.94E-01
IgG4_G2FNS	Sensitization	0.002	0.124	9.84E-01	9.94E-01
IgG1_G0F	Wheal sum	0.000	0.009	9.84E-01	9.94E-01

IgG2_N	pos_dog	0.003	0.212	9.88E-01	9.95E-01
IgG4_G1FS	pos_dog	0.003	0.216	9.90E-01	9.95E-01
IgG2_G1FS	pos_egg	-0.006	0.505	9.91E-01	9.95E-01
IgG1_G1NS	pos_egg	0.006	0.510	9.91E-01	9.95E-01
IgG1_G2F	mm_hdm	0.000	0.030	9.94E-01	9.97E-01
IgG1_G1FNS	pos_pnut	0.002	0.361	9.97E-01	9.98E-01
IgG4_G1F	mm_pnut	0.000	0.031	9.99E-01	9.99E-01

**pos_dog**	positive SPT (mean wheal diameter >= 3 mm) to dog allergen
**pos_cat**	positive SPT (mean wheal diameter >= 3 mm) to cat allergen
**pos_tg**	positive SPT (mean wheal diameter >= 3 mm) to timothy grass allergen
**pos_egg**	positive SPT (mean wheal diameter >= 3 mm) to whole hen's egg allergen
**pos_pnut**	positive SPT (mean wheal diameter >= 3 mm) to peanut allergen
**pos_hdm**	positive SPT (mean wheal diameter >= 3 mm) to house dust mite allergen
**mm_dog**	mean wheal diameter to dog allergen
**mm_cat**	mean wheal diameter to cat allergen
**mm_tg**	mean wheal diameter to timothy grass allergen
**mm_egg**	mean wheal diameter to whole hen's egg allergen
**mm_pnut**	mean wheal diameter to peanut allergen
**mm_hdm**	mean wheal diameter to house dust mite allergen
**Sensitization**	positive SPT to any allergen
**Wheal sum**	positive wheal diameter sum
**G0**	agalactosylated glycopeptides
**G1**	monogalactosylated glycopeptides
**G2**	digalactosylated glycopeptides
**F**	core fucosylated glycopeptides
**S**	sialylated glycopeptides
**N**	glycopeptides bearing a bisecting *N*- acetylglucosamine
**G0F**	*for glycan structures see Figure 2.*
**G0FN**	*for glycan structures see Figure 2.*
**G1F**	*for glycan structures see Figure 2.*
**G1FN**	*for glycan structures see Figure 2.*
**G1FS**	*for glycan structures see Figure 2.*
**G1FNS**	*for glycan structures see Figure 2.*
**G1S**	*for glycan structures see Figure 2.*
**G1NS**	*for glycan structures see Figure 2.*
**G2F**	*for glycan structures see Figure 2.*
**G2FN**	*for glycan structures see Figure 2.*
**G2FS**	*for glycan structures see Figure 2.*
**G2FNS**	*for glycan structures see Figure 2.*
**SE**	standard error

Supplemental Table 2. Zagreb cohort - No difference in IgG glycosylation pattern (12 main glycan species and 6 derived traits) was found nor between children sensitized to at least one allergen and non-sensitized children, neither in respect to sensitization to any particular allergen, single allergen mean wheal diameter, positive wheal sum values or high level of total serum IgE .

Glycan	Trait	effect	SE	p	p adjusted
IgG2_G2FS	Wheal sum	0.021	0.005	1.03E-04	0.1227
IgG2_S	Wheal sum	0.019	0.005	2.70E-04	0.1387
IgG4_G1S	pos_hazel	-0.653	0.183	3.50E-04	0.1387
IgG4_F	pos_hazel	0.618	0.183	7.14E-04	0.1418
IgG2_G0	Wheal sum	-0.018	0.005	7.27E-04	0.1418
IgG2_G2FS	mm_grass_mix	0.077	0.023	7.66E-04	0.1418
IgG2_G0F	Wheal sum	-0.018	0.005	1.10E-03	0.1418
IgG4_G1NS	pos_hazel	-0.592	0.183	1.13E-03	0.1418
IgG2_G2FS	mm_hazel	0.108	0.033	1.15E-03	0.1418
IgG2_G2FS	pos_hazel	0.581	0.181	1.24E-03	0.1418
IgG2_G2FS	pos_grass_mix	0.377	0.118	1.31E-03	0.1418
IgG2_S	pos_grass_mix	0.370	0.118	1.63E-03	0.1537
IgG2_S	mm_grass_mix	0.072	0.023	1.68E-03	0.1537
IgG4_G1S	pos_grass_mix	-0.359	0.119	2.46E-03	0.2069
IgG2_S	pos_hazel	0.538	0.181	2.79E-03	0.2069
IgG2_S	mm_hazel	0.099	0.033	2.81E-03	0.2069
IgG4_G1S	mm_hazel	-0.100	0.034	2.99E-03	0.2069
IgG2_G2FS	pos_birch	0.464	0.159	3.25E-03	0.2069
IgG2_G0	mm_hazel	-0.099	0.034	3.36E-03	0.2069
IgG2_G1FN	mm_tree_mix	0.248	0.086	3.50E-03	0.2069
IgG4_G1S	Sensitization I	-0.243	0.085	4.08E-03	0.2069
IgG2_G2F	Wheal sum	0.016	0.005	4.10E-03	0.2069
IgG2_G2FS	mm_birch	0.079	0.028	4.26E-03	0.2069
IgG4_G1FNS	pos_hazel	-0.515	0.182	4.37E-03	0.2069
IgG4_G1S	Wheal sum	-0.015	0.005	4.42E-03	0.2069
IgG2_G0F	mm_hazel	-0.096	0.034	4.53E-03	0.2069
IgG4_G1S	pos_ambrosia	-0.301	0.108	4.95E-03	0.2098
IgG2_G2	Wheal sum	0.015	0.005	5.08E-03	0.2098
IgG2_G2FS	mm_ambrosia	0.049	0.018	5.30E-03	0.2098
IgG4_G2FN	pos_cladosporium	-0.842	0.304	5.42E-03	0.2098
IgG2_G0F	mm_cat	-0.073	0.026	5.58E-03	0.2098
IgG2_G0	mm_birch	-0.077	0.028	5.77E-03	0.2098
IgG4_F	mm_hazel	0.093	0.034	5.94E-03	0.2098
IgG4_F	pos_ambrosia	0.294	0.108	6.04E-03	0.2098
IgG4_G1NS	pos_ambrosia	-0.292	0.107	6.31E-03	0.2098
IgG2_S	pos_birch	0.430	0.159	6.36E-03	0.2098
IgG4_G0F	high_IgE	0.247	0.091	6.65E-03	0.2110
IgG4_G0	high_IgE	0.243	0.090	6.91E-03	0.2110
IgG4_G0F	Sensitization II	0.254	0.095	7.12E-03	0.2110
IgG4_G1S	Sensitization II	-0.254	0.095	7.20E-03	0.2110
IgG2_G0F	mm_birch	-0.075	0.028	7.38E-03	0.2110
IgG4_F	pos_grass_mix	0.316	0.119	7.71E-03	0.2110
IgG2_G2	mm_cat	0.070	0.027	7.81E-03	0.2110
IgG2_S	mm_birch	0.073	0.028	7.83E-03	0.2110
IgG4_G1NS	mm_hazel	-0.088	0.034	8.29E-03	0.2110
IgG2_G2FS	pos_ambrosia	0.278	0.106	8.59E-03	0.2110
IgG4_G0	Sensitization II	0.245	0.094	8.73E-03	0.2110
IgG4_G1NS	pos_birch	-0.418	0.161	8.82E-03	0.2110
IgG4_F	Sensitization II	0.246	0.095	9.00E-03	0.2110

IgG2_G0	pos_hazel	-0.477	0.185	9.30E-03	0.2110
IgG4_G0F	Sensitization I	0.219	0.085	9.49E-03	0.2110
IgG2_G0	pos_birch	-0.415	0.161	9.69E-03	0.2110
IgG2_G0	mm_cat	-0.068	0.026	9.83E-03	0.2110
IgG4_G0F	pos_cladosporium	0.773	0.302	1.01E-02	0.2110
IgG4_G1S	pos_birch	-0.414	0.162	1.01E-02	0.2110
IgG4_G1S	mm_birch	-0.072	0.028	1.05E-02	0.2110
IgG2_S	mm_ambrosia	0.045	0.018	1.05E-02	0.2110
IgG1_G1	pos_hdm	-0.232	0.091	1.05E-02	0.2110
IgG4_F	pos_birch	0.408	0.162	1.10E-02	0.2110
IgG4_F	Sensitization I	0.215	0.085	1.11E-02	0.2110
IgG2_G1FN	pos_tree_mix	0.968	0.387	1.13E-02	0.2110
IgG2_G2F	mm_cat	0.067	0.027	1.16E-02	0.2110
IgG4_F	Wheal sum	0.014	0.005	1.16E-02	0.2110
IgG2_G1FS	Wheal sum	0.014	0.005	1.18E-02	0.2110
IgG2_G2F	mm_hazel	0.085	0.034	1.19E-02	0.2110
IgG2_G0F	pos_grass_mix	-0.299	0.120	1.19E-02	0.2110
IgG2_G0	mm_grass_mix	-0.059	0.023	1.20E-02	0.2110
IgG2_G0F	pos_hazel	-0.461	0.185	1.21E-02	0.2110
IgG2_G0	pos_grass_mix	-0.296	0.119	1.26E-02	0.2164
IgG2_G2	mm_dog	0.098	0.040	1.30E-02	0.2164
IgG2_G0	mm_ambrosia	-0.044	0.018	1.30E-02	0.2164
IgG4_G1NS	pos_grass_mix	-0.294	0.119	1.32E-02	0.2164
IgG4_G1NS	mm_ambrosia	-0.044	0.018	1.36E-02	0.2164
IgG4_G0	Sensitization I	0.206	0.084	1.37E-02	0.2164
IgG2_G1FS	pos_grass_mix	0.293	0.119	1.38E-02	0.2164
IgG2_G2FS	Sensitization I	0.205	0.084	1.43E-02	0.2164
IgG2_G1FS	mm_grass_mix	0.057	0.023	1.45E-02	0.2164
IgG4_G1NS	Sensitization II	-0.230	0.095	1.46E-02	0.2164
IgG2_G0F	pos_birch	-0.392	0.162	1.48E-02	0.2164
IgG2_G0F	mm_dog	-0.096	0.039	1.49E-02	0.2164
IgG4_G1FNS	pos_ambrosia	-0.258	0.107	1.49E-02	0.2164
IgG2_G0	mm_dog	-0.095	0.039	1.51E-02	0.2164
IgG2_G0F	mm_grass_mix	-0.057	0.023	1.51E-02	0.2164
IgG4_F	mm_ambrosia	0.043	0.018	1.57E-02	0.2205
IgG1_G1F	pos_hdm	-0.228	0.095	1.58E-02	0.2205
IgG2_S	pos_ambrosia	0.256	0.107	1.60E-02	0.2206
IgG2_G2FS	mm_cat	0.062	0.026	1.67E-02	0.2238
IgG1_S	mm_cat	0.061	0.026	1.67E-02	0.2238
IgG4_G1S	mm_ambrosia	-0.043	0.018	1.68E-02	0.2238
IgG4_G1S	mm_grass_mix	-0.056	0.023	1.73E-02	0.2287
IgG2_G2	mm_hazel	0.080	0.034	1.78E-02	0.2316
IgG2_G1FS	pos_hazel	0.435	0.186	1.80E-02	0.2316
IgG4_F	high_IgE	0.216	0.092	1.81E-02	0.2316
IgG2_G2F	mm_dog	0.093	0.040	1.87E-02	0.2342
IgG4_N	pos_birch	-0.366	0.157	1.89E-02	0.2342
IgG1_G1FN	pos_dog	-0.393	0.168	1.91E-02	0.2342
IgG4_N	pos_hazel	-0.417	0.180	1.93E-02	0.2342
IgG4_G1NS	mm_birch	-0.065	0.028	1.93E-02	0.2342
IgG4_G1FN	pos_cladosporium	-0.702	0.302	1.97E-02	0.2342

IgG4_F	mm_birch	0.065	0.028	1.97E-02	0.2342
IgG4_G1NS	Wheal sum	-0.013	0.005	2.00E-02	0.2354
IgG4_G2FNS	pos_ambrosia	-0.247	0.107	2.06E-02	0.2389
IgG1_S	Wheal sum	0.012	0.005	2.07E-02	0.2389
IgG4_G2FS	pos_birch	0.371	0.162	2.10E-02	0.2403
IgG4_G1S	high_IgE	-0.210	0.092	2.17E-02	0.2414
IgG1_S	Sensitization I	0.188	0.082	2.17E-02	0.2414
IgG1_G2FS	mm_cat	0.058	0.026	2.17E-02	0.2414
IgG2_S	Sensitization I	0.190	0.084	2.30E-02	0.2525
IgG4_G1	mm_dog	-0.084	0.037	2.32E-02	0.2525
IgG1_N	pos_dog	-0.386	0.171	2.34E-02	0.2525
IgG4_G1NS	high_IgE	-0.206	0.092	2.38E-02	0.2543
IgG2_G0F	mm_ambrosia	-0.040	0.018	2.40E-02	0.2543
IgG1_G2FS	Wheal sum	0.012	0.005	2.42E-02	0.2543
IgG4_G1FNS	pos_birch	-0.356	0.160	2.46E-02	0.2543
IgG2_G2F	mm_birch	0.063	0.028	2.47E-02	0.2543
IgG4_G2	high_IgE	-0.204	0.091	2.48E-02	0.2543
IgG4_G1NS	Sensitization I	-0.189	0.085	2.51E-02	0.2543
IgG4_G0F	pos_tree_mix	-0.902	0.409	2.53E-02	0.2543
IgG4_G2FN	pos_hazel	-0.408	0.185	2.60E-02	0.2592
IgG4_G1FNS	mm_ambrosia	-0.039	0.018	2.65E-02	0.2624
IgG2_S	mm_cat	0.058	0.026	2.71E-02	0.2656
IgG2_G0FN	Wheal sum	-0.012	0.005	2.78E-02	0.2710
IgG1_G2FS	Sensitization I	0.178	0.082	2.87E-02	0.2775
IgG4_G1FNS	mm_hazel	-0.073	0.034	2.90E-02	0.2775
IgG4_G1FNS	Sensitization I	-0.182	0.084	2.98E-02	0.2828
IgG2_G0F	mm_tree_mix	-0.196	0.092	3.03E-02	0.2857
IgG4_G1FN	mm_tree_mix	0.193	0.091	3.06E-02	0.2861
IgG4_G2FNS	pos_cladosporium	-0.648	0.302	3.11E-02	0.2889
IgG4_G2F	high_IgE	-0.194	0.091	3.19E-02	0.2930
IgG2_G2	mm_birch	0.060	0.028	3.21E-02	0.2930
IgG4_G2FN	mm_cladosporium	-0.157	0.074	3.34E-02	0.2993
IgG2_G2F	mm_ambrosia	0.038	0.018	3.35E-02	0.2993
IgG4_N	mm_hazel	-0.070	0.033	3.36E-02	0.2993
IgG4_G2FS	mm_birch	0.060	0.028	3.38E-02	0.2993
IgG4_G2	Sensitization II	-0.199	0.095	3.47E-02	0.3030
IgG2_G1FS	mm_birch	0.059	0.028	3.47E-02	0.3030
IgG1_G1	mm_hdm	-0.036	0.017	3.55E-02	0.3030
IgG4_G2	pos_cladosporium	-0.629	0.301	3.58E-02	0.3030
IgG1_G0F	pos_tree_mix	-0.863	0.418	3.60E-02	0.3030
IgG2_G1FS	mm_hazel	0.071	0.034	3.61E-02	0.3030
IgG4_G0F	mm_cladosporium	0.153	0.073	3.61E-02	0.3030
IgG2_G2FS	Sensitization II	0.196	0.094	3.65E-02	0.3030
IgG2_G0	mm_tree_mix	-0.189	0.092	3.65E-02	0.3030
IgG4_G1FNS	Sensitization II	-0.195	0.094	3.74E-02	0.3087
IgG2_G2F	pos_birch	0.333	0.162	3.83E-02	0.3134
IgG4_G1FN	pos_tree_mix	0.828	0.407	3.88E-02	0.3157
IgG4_G0F	mm_tree_mix	-0.185	0.091	3.95E-02	0.3178
IgG2_G0F	pos_tree_mix	-0.837	0.414	3.96E-02	0.3178
IgG2_S	Sensitization II	0.192	0.094	3.99E-02	0.3185

IgG4_G0	pos_cladosporium	0.609	0.299	4.06E-02	0.3219
IgG4_G2FNS	mm_ambrosia	-0.036	0.018	4.09E-02	0.3220
IgG4_F	mm_grass_mix	0.048	0.023	4.12E-02	0.3223
IgG2_G0	pos_tree_mix	-0.827	0.412	4.16E-02	0.3231
IgG4_G1	pos_dog	-0.331	0.164	4.27E-02	0.3267
IgG2_G0FN	mm_ambrosia	-0.036	0.018	4.28E-02	0.3267
IgG2_G2FN	mm_cat	0.053	0.026	4.29E-02	0.3267
IgG4_G1	mm_cat	-0.050	0.025	4.33E-02	0.3276
IgG4_G2FNS	pos_hazel	-0.368	0.184	4.36E-02	0.3276
IgG2_G2F	mm_grass_mix	0.047	0.024	4.47E-02	0.3333
IgG4_G1	Sensitization II	-0.180	0.090	4.49E-02	0.3333
IgG2_G1FN	pos_cladosporium	-0.585	0.295	4.62E-02	0.3386
IgG2_G2F	pos_hazel	0.366	0.185	4.62E-02	0.3386
IgG1_G0	pos_tree_mix	-0.815	0.417	4.70E-02	0.3404
IgG4_G2FNS	Sensitization I	-0.168	0.085	4.70E-02	0.3404
IgG2_G2	pos_birch	0.317	0.162	4.85E-02	0.3489
IgG4_N	mm_birch	-0.054	0.027	4.89E-02	0.3489
IgG2_G2	mm_ambrosia	0.035	0.018	4.90E-02	0.3489
IgG4_G2FN	mm_tree_mix	0.177	0.092	5.01E-02	0.3542
IgG4_G2FS	pos_tree_mix	0.785	0.410	5.16E-02	0.3619
IgG2_G2	pos_tree_mix	0.795	0.416	5.19E-02	0.3619
IgG4_G2F	Sensitization II	-0.182	0.095	5.21E-02	0.3619
IgG4_G2FN	pos_ambrosia	-0.208	0.108	5.33E-02	0.3624
IgG2_G2	mm_grass_mix	0.045	0.024	5.34E-02	0.3624
IgG4_G2FNS	Sensitization II	-0.182	0.095	5.36E-02	0.3624
IgG4_G1FNS	mm_birch	-0.053	0.028	5.36E-02	0.3624
IgG2_G2FS	pos_cat	0.272	0.142	5.39E-02	0.3624
IgG4_G2FNS	mm_tree_mix	0.173	0.092	5.40E-02	0.3624
IgG4_G1	pos_cat	-0.259	0.136	5.43E-02	0.3624
IgG4_G1F	mm_dog	-0.072	0.038	5.51E-02	0.3635
IgG1_F	mm_cladosporium	0.137	0.072	5.52E-02	0.3635
IgG1_N	mm_dog	-0.074	0.039	5.59E-02	0.3635
IgG4_G1F	mm_cat	-0.048	0.025	5.61E-02	0.3635
IgG2_G2FNS	pos_cladosporium	-0.577	0.304	5.64E-02	0.3635
IgG1_S	pos_cat	0.264	0.139	5.66E-02	0.3635
IgG4_G2	Sensitization I	-0.160	0.085	5.66E-02	0.3635
IgG4_G0F	pos_ambrosia	0.203	0.108	5.75E-02	0.3641
IgG2_G0	pos_ambrosia	-0.204	0.108	5.77E-02	0.3641
IgG2_G1FS	pos_birch	0.306	0.163	5.79E-02	0.3641
IgG1_G2FNS	mm_tree_mix	0.164	0.088	5.81E-02	0.3641
IgG1_G0F	mm_cat	-0.050	0.027	5.92E-02	0.3641
IgG4_G2FN	pos_tree_mix	0.766	0.413	5.93E-02	0.3641
IgG2_G1FS	mm_ambrosia	0.034	0.018	5.94E-02	0.3641
IgG1_F	pos_cladosporium	0.555	0.296	5.96E-02	0.3641
IgG1_G1FN	mm_tree_mix	0.161	0.087	5.97E-02	0.3641
IgG2_G2FN	mm_tree_mix	0.167	0.090	5.98E-02	0.3641
IgG4_G1FS	mm_dog	-0.074	0.040	6.02E-02	0.3643
IgG2_G2FS	mm_cladosporium	0.136	0.073	6.06E-02	0.3643
IgG2_G2F	pos_grass_mix	0.224	0.120	6.12E-02	0.3643
IgG1_G2FNS	pos_tree_mix	0.727	0.395	6.12E-02	0.3643

IgG2_G2FN	pos_tree_mix	0.746	0.405	6.13E-02	0.3643
IgG2_F	pos_dog	-0.320	0.173	6.22E-02	0.3665
IgG4_G1FN	pos_hazel	-0.341	0.185	6.23E-02	0.3665
IgG4_G1NS	mm_grass_mix	-0.043	0.023	6.29E-02	0.3678
IgG4_G1FNS	high_IgE	-0.168	0.091	6.38E-02	0.3678
IgG1_G2FS	pos_birch	0.283	0.154	6.38E-02	0.3678
IgG1_G2FN	pos_cladosporium	-0.557	0.302	6.40E-02	0.3678
IgG1_G1F	mm_hdm	-0.033	0.018	6.42E-02	0.3678
IgG1_S	pos_birch	0.283	0.155	6.50E-02	0.3678
IgG1_S	Sensitization II	0.168	0.092	6.53E-02	0.3678
IgG1_G1FN	mm_dog	-0.070	0.038	6.55E-02	0.3678
IgG2_G2F	pos_tree_mix	0.752	0.416	6.58E-02	0.3678
IgG4_G1FNS	Wheal sum	-0.010	0.005	6.59E-02	0.3678
IgG2_G2	pos_hazel	0.338	0.185	6.60E-02	0.3678
IgG4_G1	high_IgE	-0.158	0.087	6.69E-02	0.3678
IgG1_G0F	mm_tree_mix	-0.168	0.093	6.69E-02	0.3678
IgG1_G1NS	pos_cladosporium	-0.535	0.294	6.70E-02	0.3678
IgG2_G2	pos_grass_mix	0.219	0.120	6.74E-02	0.3678
IgG2_S	mm_cladosporium	0.133	0.073	6.75E-02	0.3678
IgG4_G0	pos_hdm	0.180	0.099	6.86E-02	0.3721
IgG1_G1FS	pos_cladosporium	0.548	0.304	6.95E-02	0.3753
IgG4_G1FS	pos_dog	-0.314	0.174	7.02E-02	0.3768
IgG2_G1	mm_cladosporium	-0.123	0.069	7.04E-02	0.3768
IgG4_G0	pos_tree_mix	-0.725	0.409	7.10E-02	0.3768
IgG2_S	pos_cat	0.255	0.142	7.11E-02	0.3768
IgG2_G1FN	mm_cat	0.046	0.026	7.22E-02	0.3788
IgG2_G2FN	mm_dog	0.070	0.039	7.24E-02	0.3788
IgG1_G1	Sensitization I	-0.138	0.077	7.27E-02	0.3788
IgG4_G2	mm_cladosporium	-0.131	0.073	7.27E-02	0.3788
IgG1_G2FN	pos_tree_mix	0.708	0.402	7.31E-02	0.3792
IgG1_G2FS	pos_cat	0.245	0.138	7.49E-02	0.3870
IgG2_S	mm_dog	0.069	0.039	7.53E-02	0.3871
IgG1_G0FN	mm_ambrosia	-0.032	0.018	7.61E-02	0.3891
IgG1_G0	mm_cat	-0.047	0.027	7.72E-02	0.3891
IgG4_S	pos_tree_mix	0.710	0.409	7.73E-02	0.3891
IgG4_G0	mm_hdm	0.033	0.019	7.75E-02	0.3891
IgG2_G0F	pos_cat	-0.252	0.144	7.77E-02	0.3891
IgG4_G2F	pos_cladosporium	-0.527	0.301	7.79E-02	0.3891
IgG2_G0F	pos_ambrosia	-0.190	0.108	7.79E-02	0.3891
IgG2_G2	mm_tree_mix	0.160	0.093	7.86E-02	0.3904
IgG4_G0	mm_cladosporium	0.127	0.073	7.89E-02	0.3904
IgG4_G1S	pos_dog	-0.303	0.174	7.95E-02	0.3921
IgG1_G1FN	pos_tree_mix	0.670	0.390	8.02E-02	0.3936
IgG2_G0FN	mm_grass_mix	-0.041	0.023	8.05E-02	0.3936
IgG2_G0FN	mm_hazel	-0.059	0.034	8.11E-02	0.3947
IgG4_S	high_IgE	-0.160	0.092	8.14E-02	0.3948
IgG4_F	pos_dog	0.300	0.174	8.26E-02	0.3989
IgG4_G1FS	mm_ambrosia	-0.031	0.018	8.56E-02	0.4079
IgG4_G2FNS	high_IgE	-0.156	0.092	8.58E-02	0.4079
IgG4_G2FNS	mm_cladosporium	-0.125	0.073	8.65E-02	0.4079

IgG1_G2FS	mm_birch	0.046	0.027	8.68E-02	0.4079
IgG2_G1FS	pos_ambrosia	0.184	0.108	8.71E-02	0.4079
IgG4_G0F	mm_ambrosia	0.030	0.018	8.72E-02	0.4079
IgG1_G2FN	pos_dog	-0.294	0.173	8.74E-02	0.4079
IgG4_G2FN	mm_ambrosia	-0.031	0.018	8.74E-02	0.4079
IgG1_G1NS	mm_cladosporium	-0.121	0.071	8.76E-02	0.4079
IgG4_G2FN	Sensitization I	-0.145	0.086	8.85E-02	0.4107
IgG2_G1S	pos_dog	0.292	0.173	8.90E-02	0.4115
IgG1_G2FS	Sensitization II	0.154	0.091	8.95E-02	0.4121
IgG1_G1FS	Wheal sum	0.009	0.005	8.98E-02	0.4121
IgG1_G1FN	pos_cladosporium	-0.496	0.295	9.08E-02	0.4142
IgG2_S	high_IgE	0.153	0.091	9.11E-02	0.4142
IgG4_F	mm_dog	0.066	0.039	9.16E-02	0.4142
IgG4_G1	Sensitization I	-0.135	0.081	9.21E-02	0.4142
IgG1_G1	pos_cladosporium	-0.461	0.276	9.24E-02	0.4142
IgG4_G1S	mm_cat	-0.044	0.027	9.24E-02	0.4142
IgG4_S	mm_tree_mix	0.150	0.091	9.28E-02	0.4143
IgG2_G2FS	high_IgE	0.151	0.091	9.40E-02	0.4182
IgG4_G0	mm_tree_mix	-0.149	0.091	9.52E-02	0.4220
IgG4_G0	pos_ambrosia	0.176	0.107	9.62E-02	0.4248
IgG4_G1FN	pos_birch	-0.266	0.162	9.71E-02	0.4255
IgG1_S	mm_birch	0.044	0.027	9.73E-02	0.4255
IgG4_G1S	mm_dog	-0.065	0.040	9.80E-02	0.4255
IgG2_G0	pos_cat	-0.236	0.144	9.81E-02	0.4255
IgG2_G2F	mm_tree_mix	0.150	0.093	9.94E-02	0.4255
IgG2_G2FS	mm_dog	0.064	0.039	9.96E-02	0.4255
IgG1_G1FNS	mm_tree_mix	0.146	0.090	1.00E-01	0.4255
IgG1_S	pos_tree_mix	0.662	0.410	1.00E-01	0.4255
IgG1_G2FNS	mm_cat	0.043	0.026	1.01E-01	0.4255
IgG1_G1S	mm_cladosporium	-0.119	0.073	1.01E-01	0.4255
IgG4_G2F	Sensitization I	-0.138	0.084	1.01E-01	0.4255
IgG1_G0FN	pos_ambrosia	-0.176	0.108	1.01E-01	0.4255
IgG1_G2FN	mm_tree_mix	0.144	0.089	1.01E-01	0.4255
IgG2_G1FS	pos_cladosporium	0.491	0.304	1.04E-01	0.4339
IgG4_F	mm_cat	0.043	0.027	1.04E-01	0.4339
IgG4_G1NS	mm_cat	-0.043	0.026	1.05E-01	0.4339
IgG2_G2FNS	mm_cladosporium	-0.119	0.074	1.05E-01	0.4339
IgG2_G2FN	pos_cladosporium	-0.488	0.303	1.05E-01	0.4339
IgG2_G0	pos_dog	-0.279	0.174	1.06E-01	0.4357
IgG1_G1FN	mm_hazel	-0.053	0.033	1.06E-01	0.4357
IgG2_G2	pos_cat	0.232	0.144	1.06E-01	0.4357
IgG4_G2FN	mm_hazel	-0.054	0.034	1.08E-01	0.4396
IgG1_G1	mm_cladosporium	-0.107	0.067	1.09E-01	0.4403
IgG1_N	mm_hazel	-0.054	0.034	1.09E-01	0.4403
IgG4_G2FS	mm_cat	0.042	0.027	1.09E-01	0.4403
IgG1_G1FS	mm_hdm	0.030	0.019	1.10E-01	0.4427
IgG1_S	mm_tree_mix	0.142	0.091	1.12E-01	0.4483
IgG4_G1F	pos_cat	-0.216	0.137	1.13E-01	0.4483
IgG1_G1FNS	pos_tree_mix	0.631	0.406	1.13E-01	0.4483
IgG1_G0	mm_tree_mix	-0.145	0.093	1.14E-01	0.4483

IgG4_G2FS	mm_tree_mix	0.142	0.092	1.14E-01	0.4483
IgG2_G1	pos_cladosporium	-0.443	0.283	1.14E-01	0.4483
IgG2_S	pos_dog	0.270	0.172	1.15E-01	0.4483
IgG4_G2FNS	pos_tree_mix	0.638	0.412	1.15E-01	0.4483
IgG2_G2	pos_dog	0.274	0.175	1.15E-01	0.4483
IgG4_G2FNS	pos_dog	-0.270	0.173	1.16E-01	0.4483
IgG2_N	mm_ambrosia	-0.028	0.018	1.16E-01	0.4483
IgG1_G1FS	Sensitization II	0.148	0.095	1.17E-01	0.4483
IgG4_G1F	pos_hdm	-0.150	0.096	1.17E-01	0.4483
IgG2_G0F	pos_dog	-0.271	0.174	1.17E-01	0.4483
IgG4_G1S	mm_cladosporium	-0.115	0.074	1.18E-01	0.4483
IgG4_G0FN	pos_birch	-0.241	0.156	1.18E-01	0.4483
IgG1_G1	Sensitization II	-0.135	0.087	1.18E-01	0.4483
IgG4_G2FN	pos_birch	-0.251	0.162	1.18E-01	0.4483
IgG4_G2	pos_ambrosia	-0.167	0.107	1.18E-01	0.4483
IgG4_G2FNS	Wheal sum	-0.008	0.005	1.19E-01	0.4497
IgG2_G1	mm_tree_mix	0.132	0.086	1.20E-01	0.4497
IgG1_G1FS	high_IgE	0.142	0.092	1.20E-01	0.4497
IgG4_G1FN	pos_dog	-0.266	0.173	1.23E-01	0.4558
IgG2_G0FN	pos_grass_mix	-0.183	0.119	1.23E-01	0.4558
IgG4_G0F	pos_hdm	0.154	0.101	1.23E-01	0.4558
IgG4_G2F	mm_cladosporium	-0.112	0.073	1.23E-01	0.4558
IgG2_N	pos_cladosporium	-0.462	0.303	1.24E-01	0.4577
IgG1_N	mm_ambrosia	-0.027	0.018	1.25E-01	0.4580
IgG2_G2F	pos_cat	0.219	0.144	1.27E-01	0.4657
IgG1_G2FS	pos_tree_mix	0.611	0.408	1.28E-01	0.4667
IgG4_G0F	pos_dog	0.262	0.173	1.29E-01	0.4679
IgG4_G0F	mm_hdm	0.028	0.019	1.29E-01	0.4679
IgG1_S	mm_hdm	0.027	0.018	1.29E-01	0.4690
IgG4_G1FN	mm_hazel	-0.051	0.034	1.30E-01	0.4698
IgG4_G2F	pos_hdm	-0.150	0.100	1.31E-01	0.4698
IgG4_G1FS	mm_cat	-0.040	0.027	1.32E-01	0.4734
IgG1_G1S	pos_cladosporium	-0.448	0.300	1.32E-01	0.4734
IgG4_S	Sensitization II	-0.143	0.096	1.33E-01	0.4746
IgG4_G1FNS	pos_grass_mix	-0.177	0.118	1.34E-01	0.4751
IgG2_S	pos_cladosporium	0.448	0.301	1.34E-01	0.4751
IgG2_G0FN	mm_dog	-0.059	0.039	1.35E-01	0.4760
IgG4_S	pos_cladosporium	-0.454	0.305	1.35E-01	0.4760
IgG4_G1	pos_hdm	-0.141	0.095	1.36E-01	0.4767
IgG4_S	pos_ambrosia	-0.161	0.109	1.36E-01	0.4767
IgG4_G0	mm_ambrosia	0.026	0.018	1.37E-01	0.4783
IgG4_G1	mm_hdm	-0.026	0.018	1.38E-01	0.4800
IgG1_G2FNS	pos_ambrosia	0.157	0.107	1.38E-01	0.4801
IgG1_G1FN	pos_hazel	-0.264	0.182	1.42E-01	0.4906
IgG2_G1FS	high_IgE	0.134	0.092	1.42E-01	0.4906
IgG2_G2F	pos_ambrosia	0.158	0.109	1.42E-01	0.4906
IgG1_G2FN	mm_cat	0.038	0.026	1.44E-01	0.4921
IgG4_G2FS	mm_hazel	0.050	0.034	1.44E-01	0.4921
IgG4_G2FN	mm_birch	-0.041	0.028	1.45E-01	0.4945
IgG2_G2F	pos_dog	0.252	0.175	1.46E-01	0.4950

IgG4_G2F	mm_hdm	-0.027	0.019	1.46E-01	0.4950
IgG1_G0FN	mm_dog	-0.057	0.039	1.47E-01	0.4960
IgG1_G2	pos_cladosporium	-0.439	0.305	1.47E-01	0.4974
IgG2_G1FS	Sensitization II	0.137	0.095	1.48E-01	0.4978
IgG2_G1FS	mm_cladosporium	0.106	0.074	1.49E-01	0.4978
IgG4_G1F	mm_hdm	-0.026	0.018	1.49E-01	0.4978
IgG1_G2	mm_cat	0.038	0.027	1.49E-01	0.4978
IgG4_G2FNS	mm_hazel	-0.048	0.034	1.52E-01	0.5035
IgG1_S	pos_hdm	0.139	0.098	1.52E-01	0.5035
IgG2_G2FS	pos_cladosporium	0.427	0.301	1.53E-01	0.5055
IgG1_G2FNS	pos_cladosporium	-0.426	0.301	1.54E-01	0.5055
IgG1_F	pos_dog	0.240	0.170	1.54E-01	0.5055
IgG1_G2FS	mm_tree_mix	0.127	0.091	1.54E-01	0.5055
IgG2_G0	Sensitization I	-0.121	0.085	1.54E-01	0.5055
IgG2_G0FN	pos_dog	-0.245	0.173	1.55E-01	0.5072
IgG4_G2	pos_hdm	-0.141	0.100	1.58E-01	0.5112
IgG4_G1F	Sensitization II	-0.128	0.091	1.58E-01	0.5112
IgG2_G0FN	pos_birch	-0.226	0.162	1.58E-01	0.5112
IgG2_N	mm_hazel	-0.048	0.034	1.59E-01	0.5112
IgG4_N	pos_ambrosia	-0.148	0.106	1.59E-01	0.5112
IgG4_G0	pos_dog	0.240	0.172	1.60E-01	0.5112
IgG1_S	mm_grass_mix	0.032	0.023	1.60E-01	0.5112
IgG1_G2	pos_tree_mix	0.578	0.419	1.60E-01	0.5116
IgG1_N	mm_birch	-0.039	0.028	1.61E-01	0.5118
IgG2_N	mm_grass_mix	-0.033	0.023	1.61E-01	0.5118
IgG1_G1FS	mm_grass_mix	0.033	0.023	1.62E-01	0.5130
IgG4_N	pos_cladosporium	-0.414	0.299	1.63E-01	0.5130
IgG2_G0FN	mm_birch	-0.039	0.028	1.63E-01	0.5130
IgG1_G2FS	mm_hdm	0.025	0.018	1.63E-01	0.5130
IgG4_G1FN	mm_birch	-0.039	0.028	1.64E-01	0.5130
IgG2_G0FN	pos_hazel	-0.256	0.186	1.64E-01	0.5132
IgG2_G1NS	pos_hazel	0.254	0.185	1.65E-01	0.5136
IgG4_G1FS	Wheal sum	-0.008	0.005	1.65E-01	0.5143
IgG4_G1FN	pos_ambrosia	-0.148	0.108	1.66E-01	0.5146
IgG4_S	mm_ambrosia	-0.025	0.018	1.67E-01	0.5152
IgG2_G1S	mm_dog	0.054	0.039	1.67E-01	0.5152
IgG1_N	mm_tree_mix	0.120	0.089	1.68E-01	0.5152
IgG1_G2FS	pos_hdm	0.133	0.097	1.69E-01	0.5152
IgG1_N	pos_birch	-0.218	0.160	1.69E-01	0.5152
IgG1_G1FS	mm_cladosporium	0.101	0.074	1.69E-01	0.5152
IgG1_G1FNS	pos_dog	-0.238	0.174	1.69E-01	0.5152
IgG2_G0FN	pos_ambrosia	-0.147	0.108	1.70E-01	0.5164
IgG4_G2FN	Sensitization II	-0.131	0.096	1.70E-01	0.5165
IgG1_G1FNS	Sensitization I	0.116	0.086	1.71E-01	0.5173
IgG1_G2F	mm_cat	0.036	0.027	1.72E-01	0.5173
IgG4_G1F	high_IgE	-0.119	0.088	1.73E-01	0.5185
IgG4_G0FN	mm_hazel	-0.044	0.033	1.73E-01	0.5185
IgG4_G1FN	mm_cladosporium	-0.100	0.074	1.73E-01	0.5185
IgG4_G1	Wheal sum	-0.007	0.005	1.74E-01	0.5185
IgG1_G0FN	pos_dog	-0.234	0.174	1.74E-01	0.5185

IgG4_G2	mm_hdm	-0.025	0.019	1.75E-01	0.5185
IgG1_G2FS	mm_dog	0.051	0.038	1.76E-01	0.5186
IgG2_G1NS	pos_dog	0.234	0.174	1.76E-01	0.5186
IgG2_G0F	Sensitization I	-0.115	0.086	1.76E-01	0.5186
IgG2_N	Wheal sum	-0.007	0.005	1.78E-01	0.5208
IgG4_G1F	pos_dog	-0.222	0.166	1.78E-01	0.5208
IgG1_G1FS	pos_cat	0.192	0.144	1.78E-01	0.5208
IgG1_N	Wheal sum	-0.007	0.005	1.78E-01	0.5208
IgG2_G1F	mm_cladosporium	-0.092	0.070	1.81E-01	0.5273
IgG1_G2F	pos_cladosporium	-0.404	0.304	1.82E-01	0.5273
IgG4_N	mm_ambrosia	-0.023	0.018	1.84E-01	0.5313
IgG4_G2F	pos_ambrosia	-0.141	0.107	1.85E-01	0.5313
IgG2_G2	pos_ambrosia	0.143	0.109	1.86E-01	0.5313
IgG4_G1S	pos_cat	-0.189	0.144	1.86E-01	0.5313
IgG4_F	pos_cat	0.188	0.144	1.87E-01	0.5313
IgG4_G0FN	pos_hdm	0.129	0.098	1.87E-01	0.5313
IgG2_G2FS	mm_hdm	0.024	0.019	1.87E-01	0.5313
IgG2_G2FS	pos_dog	0.225	0.172	1.87E-01	0.5313
IgG2_N	pos_hazel	-0.243	0.187	1.88E-01	0.5313
IgG4_G1FN	mm_ambrosia	-0.023	0.018	1.88E-01	0.5313
IgG1_G0F	pos_cladosporium	0.400	0.306	1.88E-01	0.5313
IgG4_G1FS	Sensitization II	-0.125	0.096	1.88E-01	0.5313
IgG2_G2	pos_cladosporium	-0.399	0.306	1.89E-01	0.5313
IgG4_G1FNS	pos_dog	-0.225	0.172	1.89E-01	0.5313
IgG4_G1FS	pos_grass_mix	-0.157	0.120	1.90E-01	0.5313
IgG4_G2	pos_tree_mix	0.527	0.411	1.91E-01	0.5344
IgG4_G2FNS	pos_birch	-0.208	0.161	1.92E-01	0.5348
IgG1_N	pos_hazel	-0.236	0.184	1.93E-01	0.5362
IgG4_S	mm_cladosporium	-0.096	0.074	1.93E-01	0.5362
IgG4_G1FS	high_IgE	-0.119	0.092	1.94E-01	0.5363
IgG1_S	pos_grass_mix	0.150	0.116	1.94E-01	0.5363
IgG1_N	pos_cladosporium	-0.386	0.300	1.95E-01	0.5363
IgG2_F	mm_dog	-0.051	0.039	1.96E-01	0.5363
IgG1_G0FN	Wheal sum	-0.007	0.005	1.96E-01	0.5363
IgG1_G1FS	pos_ambrosia	0.138	0.108	1.97E-01	0.5363
IgG4_G1NS	pos_cat	-0.183	0.143	1.98E-01	0.5363
IgG1_G1F	Sensitization I	-0.103	0.081	1.98E-01	0.5363
IgG1_S	pos_ambrosia	0.134	0.105	1.99E-01	0.5363
IgG1_G1FNS	pos_hdm	0.129	0.101	1.99E-01	0.5363
IgG1_G2FS	mm_grass_mix	0.029	0.023	1.99E-01	0.5363
IgG4_G0FN	mm_hdm	0.023	0.018	1.99E-01	0.5363
IgG1_G2F	pos_tree_mix	0.527	0.419	1.99E-01	0.5363
IgG4_G1S	pos_cladosporium	-0.388	0.304	2.00E-01	0.5363
IgG4_G1FS	pos_ambrosia	-0.138	0.108	2.00E-01	0.5366
IgG1_S	mm_dog	0.048	0.038	2.03E-01	0.5438
IgG2_G1NS	mm_hazel	0.043	0.034	2.04E-01	0.5446
IgG2_G1FS	mm_dog	0.050	0.040	2.05E-01	0.5457
IgG1_G1FN	mm_birch	-0.035	0.028	2.05E-01	0.5457
IgG2_G1NS	pos_birch	0.202	0.162	2.07E-01	0.5473
IgG1_G1FN	pos_birch	-0.199	0.159	2.07E-01	0.5473

IgG1_G1FNS	Sensitization II	0.120	0.096	2.07E-01	0.5473
IgG1_G2FS	mm_hazel	0.040	0.032	2.08E-01	0.5483
IgG4_G0F	Wheal sum	0.007	0.005	2.09E-01	0.5483
IgG1_N	pos_ambrosia	-0.133	0.107	2.09E-01	0.5483
IgG4_S	Sensitization I	-0.107	0.086	2.11E-01	0.5522
IgG4_G1NS	pos_dog	-0.215	0.174	2.12E-01	0.5541
IgG2_G2FNS	pos_cat	0.177	0.144	2.15E-01	0.5599
IgG1_G1FN	Wheal sum	-0.007	0.005	2.16E-01	0.5599
IgG4_G2	mm_ambrosia	-0.022	0.018	2.16E-01	0.5599
IgG2_S	mm_hdm	0.023	0.019	2.16E-01	0.5602
IgG2_G1NS	Sensitization I	0.105	0.085	2.18E-01	0.5621
IgG1_G1NS	mm_ambrosia	-0.021	0.017	2.19E-01	0.5629
IgG4_G2	mm_tree_mix	0.110	0.092	2.20E-01	0.5629
IgG1_G1FN	mm_ambrosia	-0.021	0.017	2.20E-01	0.5629
IgG2_G1NS	mm_birch	0.034	0.028	2.20E-01	0.5629
IgG1_G1NS	pos_dog	-0.205	0.168	2.20E-01	0.5629
IgG2_N	Sensitization I	-0.103	0.085	2.21E-01	0.5629
IgG4_N	Wheal sum	-0.007	0.005	2.22E-01	0.5658
IgG2_G2FN	pos_dog	0.210	0.173	2.23E-01	0.5660
IgG2_G1FS	Sensitization I	0.103	0.085	2.25E-01	0.5670
IgG4_G1FS	pos_birch	-0.196	0.163	2.25E-01	0.5670
IgG1_G2	mm_cladosporium	-0.089	0.074	2.25E-01	0.5670
IgG4_G2FNS	pos_grass_mix	-0.144	0.119	2.26E-01	0.5670
IgG2_G2F	Sensitization I	0.103	0.086	2.26E-01	0.5670
IgG4_G1NS	mm_dog	-0.047	0.039	2.27E-01	0.5670
IgG4_G0FN	high_IgE	0.107	0.089	2.27E-01	0.5670
IgG1_G1S	pos_birch	0.191	0.160	2.28E-01	0.5670
IgG1_S	high_IgE	0.107	0.089	2.28E-01	0.5670
IgG2_G2F	pos_cladosporium	-0.366	0.306	2.28E-01	0.5673
IgG4_G1FS	Sensitization I	-0.102	0.086	2.30E-01	0.5705
IgG4_G2FNS	mm_birch	-0.033	0.028	2.32E-01	0.5736
IgG1_S	mm_ambrosia	0.021	0.017	2.32E-01	0.5736
IgG2_G1	pos_tree_mix	0.455	0.389	2.33E-01	0.5736
IgG1_G1S	pos_dog	-0.203	0.172	2.34E-01	0.5744
IgG1_G2FNS	pos_dog	-0.203	0.172	2.35E-01	0.5764
IgG2_G1FN	mm_cladosporium	-0.085	0.072	2.36E-01	0.5767
IgG4_G2FN	Wheal sum	-0.006	0.005	2.36E-01	0.5767
IgG4_G0FN	Sensitization II	0.109	0.093	2.37E-01	0.5776
IgG4_G2FS	pos_cat	0.169	0.144	2.37E-01	0.5779
IgG1_G2F	mm_cladosporium	-0.086	0.074	2.39E-01	0.5818
IgG1_G1FS	mm_cat	0.031	0.027	2.41E-01	0.5835
IgG2_N	pos_ambrosia	-0.125	0.108	2.42E-01	0.5850
IgG2_G0	Sensitization II	-0.111	0.096	2.42E-01	0.5854
IgG2_G1FS	mm_tree_mix	0.106	0.093	2.45E-01	0.5891
IgG2_G2FNS	mm_cat	0.031	0.027	2.45E-01	0.5891
IgG1_G2FS	pos_grass_mix	0.133	0.115	2.46E-01	0.5898
IgG2_G1FN	Sensitization I	-0.095	0.083	2.47E-01	0.5898
IgG2_N	Sensitization II	-0.109	0.095	2.47E-01	0.5898
IgG1_N	pos_tree_mix	0.453	0.399	2.47E-01	0.5898
IgG2_G1F	mm_tree_mix	0.099	0.087	2.49E-01	0.5920

IgG2_G1FNS	pos_dog	0.199	0.174	2.50E-01	0.5939
IgG2_G0FN	Sensitization I	-0.097	0.085	2.50E-01	0.5939
IgG1_G1FS	Sensitization I	0.097	0.085	2.54E-01	0.6005
IgG1_G1FS	pos_grass_mix	0.136	0.120	2.54E-01	0.6005
IgG1_G0	Wheal sum	-0.006	0.005	2.55E-01	0.6015
IgG1_G2FNS	Sensitization I	0.095	0.084	2.56E-01	0.6015
IgG1_G0FN	pos_birch	-0.182	0.162	2.56E-01	0.6015
IgG1_G1	high_IgE	-0.094	0.084	2.58E-01	0.6039
IgG4_G1FN	Sensitization I	-0.095	0.085	2.63E-01	0.6139
IgG2_G2FS	pos_tree_mix	0.450	0.411	2.64E-01	0.6143
IgG1_S	mm_hazel	0.036	0.033	2.65E-01	0.6143
IgG4_G2F	pos_tree_mix	0.447	0.409	2.65E-01	0.6143
IgG2_G1FS	pos_dog	0.193	0.174	2.66E-01	0.6143
IgG4_G0FN	mm_birch	-0.030	0.027	2.66E-01	0.6143
IgG2_G2	Sensitization I	0.095	0.086	2.66E-01	0.6143
IgG4_G1FS	mm_birch	-0.031	0.028	2.67E-01	0.6143
IgG1_G0FN	mm_birch	-0.031	0.028	2.67E-01	0.6143
IgG4_G0	Wheal sum	0.006	0.005	2.70E-01	0.6159
IgG1_G1F	mm_cladosporium	-0.076	0.070	2.70E-01	0.6159
IgG2_G1FNS	mm_grass_mix	-0.026	0.024	2.71E-01	0.6159
IgG4_G1F	Sensitization I	-0.089	0.081	2.71E-01	0.6159
IgG4_G1FN	Sensitization II	-0.104	0.096	2.71E-01	0.6159
IgG1_G1NS	pos_tree_mix	0.421	0.390	2.71E-01	0.6159
IgG1_G2FS	mm_ambrosia	0.019	0.017	2.71E-01	0.6159
IgG1_G1FNS	mm_hdm	0.021	0.019	2.72E-01	0.6159
IgG2_G2FN	pos_cat	0.156	0.143	2.72E-01	0.6159
IgG1_G0	pos_cladosporium	0.332	0.306	2.74E-01	0.6176
IgG1_G1	pos_hazel	-0.183	0.169	2.74E-01	0.6176
IgG1_G1F	mm_grass_mix	0.024	0.022	2.75E-01	0.6176
IgG2_G2FNS	pos_tree_mix	0.448	0.418	2.75E-01	0.6176
IgG1_G2FS	pos_hazel	0.191	0.177	2.76E-01	0.6176
IgG1_G2FS	pos_ambrosia	0.113	0.104	2.77E-01	0.6181
IgG4_G0FN	pos_hazel	-0.191	0.178	2.77E-01	0.6181
IgG1_G2FN	mm_cladosporium	-0.079	0.074	2.77E-01	0.6181
IgG1_G2FN	mm_hazel	-0.037	0.034	2.78E-01	0.6189
IgG1_G1FNS	mm_dog	-0.043	0.040	2.79E-01	0.6189
IgG2_G0F	Sensitization II	-0.103	0.096	2.80E-01	0.6189
IgG4_G2FS	Wheal sum	0.006	0.005	2.80E-01	0.6189
IgG1_G2FS	high_IgE	0.094	0.089	2.85E-01	0.6298
IgG4_G2FN	high_IgE	-0.098	0.093	2.88E-01	0.6318
IgG1_G2F	mm_dog	0.042	0.040	2.88E-01	0.6318
IgG4_G0FN	Sensitization I	0.087	0.083	2.89E-01	0.6318
IgG4_G2FN	pos_dog	-0.184	0.175	2.89E-01	0.6318
IgG1_G1FNS	high_IgE	0.097	0.092	2.89E-01	0.6318
IgG2_N	high_IgE	-0.096	0.092	2.89E-01	0.6318
IgG4_G1FN	Wheal sum	-0.006	0.005	2.91E-01	0.6343
IgG1_G0	mm_grass_mix	-0.025	0.024	2.94E-01	0.6386
IgG2_G1FN	pos_cat	0.146	0.140	2.94E-01	0.6386
IgG2_F	mm_tree_mix	0.094	0.092	2.95E-01	0.6402
IgG1_G1FS	mm_dog	0.041	0.040	2.96E-01	0.6402

IgG1_G1FNS	pos_ambrosia	0.112	0.108	2.96E-01	0.6402
IgG1_G0	mm_dog	-0.041	0.040	2.97E-01	0.6410
IgG1_G1FS	pos_dog	0.179	0.174	3.00E-01	0.6410
IgG1_G0F	mm_grass_mix	-0.024	0.024	3.00E-01	0.6410
IgG2_G0FN	Sensitization II	-0.098	0.095	3.00E-01	0.6410
IgG4_G2F	mm_ambrosia	-0.018	0.018	3.00E-01	0.6410
IgG2_F	pos_tree_mix	0.419	0.412	3.00E-01	0.6410
IgG1_S	pos_hazel	0.182	0.178	3.01E-01	0.6411
IgG1_G1FS	mm_ambrosia	0.018	0.018	3.03E-01	0.6446
IgG2_G1	mm_dog	0.038	0.037	3.03E-01	0.6446
IgG2_N	pos_grass_mix	-0.122	0.119	3.05E-01	0.6455
IgG2_S	pos_tree_mix	0.416	0.414	3.05E-01	0.6455
IgG2_G1FS	mm_cat	0.027	0.027	3.06E-01	0.6455
IgG1_G1F	mm_dog	0.038	0.037	3.06E-01	0.6455
IgG1_G1F	pos_dog	0.166	0.164	3.09E-01	0.6495
IgG4_F	mm_cladosporium	0.075	0.074	3.09E-01	0.6495
IgG4_G2FS	pos_hazel	0.187	0.186	3.11E-01	0.6527
IgG4_G0F	pos_hazel	0.185	0.185	3.12E-01	0.6536
IgG4_G2FS	mm_grass_mix	0.024	0.024	3.12E-01	0.6536
IgG2_G2FS	pos_hdm	0.100	0.100	3.14E-01	0.6552
IgG1_G1FNS	pos_grass_mix	0.120	0.120	3.15E-01	0.6576
IgG2_G2FNS	pos_dog	0.173	0.174	3.17E-01	0.6597
IgG4_G2F	mm_tree_mix	0.089	0.091	3.18E-01	0.6597
IgG4_G1FNS	mm_tree_mix	0.089	0.091	3.19E-01	0.6619
IgG4_G1FS	mm_hazel	-0.034	0.034	3.21E-01	0.6649
IgG4_G2FS	pos_grass_mix	0.118	0.120	3.22E-01	0.6657
IgG1_G1NS	mm_tree_mix	0.084	0.087	3.24E-01	0.6676
IgG1_F	mm_dog	0.038	0.039	3.25E-01	0.6678
IgG1_G1FN	pos_ambrosia	-0.103	0.105	3.25E-01	0.6678
IgG2_G1NS	pos_ambrosia	0.106	0.108	3.26E-01	0.6683
IgG2_G1S	mm_cladosporium	0.072	0.073	3.27E-01	0.6691
IgG1_G1F	mm_ambrosia	0.016	0.017	3.28E-01	0.6712
IgG2_G1FN	Sensitization II	-0.090	0.093	3.29E-01	0.6725
IgG4_G2FS	pos_cladosporium	-0.292	0.305	3.34E-01	0.6810
IgG2_N	mm_cladosporium	-0.070	0.073	3.36E-01	0.6842
IgG4_F	pos_cladosporium	0.289	0.304	3.38E-01	0.6863
IgG4_G1FNS	mm_grass_mix	-0.022	0.023	3.39E-01	0.6863
IgG1_G2FN	pos_hazel	-0.176	0.186	3.40E-01	0.6863
IgG1_G1FS	mm_tree_mix	0.087	0.093	3.40E-01	0.6863
IgG1_G0F	mm_cladosporium	0.070	0.074	3.42E-01	0.6879
IgG2_N	mm_tree_mix	0.084	0.091	3.43E-01	0.6879
IgG1_G2	mm_tree_mix	0.087	0.094	3.43E-01	0.6879
IgG2_G1FS	pos_tree_mix	0.386	0.416	3.44E-01	0.6879
IgG1_G1FNS	mm_cat	0.025	0.027	3.44E-01	0.6879
IgG1_G1FS	pos_hdm	0.095	0.101	3.44E-01	0.6879
IgG1_G0F	Wheal sum	-0.005	0.005	3.45E-01	0.6881
IgG1_G2FN	mm_dog	-0.037	0.039	3.47E-01	0.6893
IgG2_G2FN	Wheal sum	0.005	0.005	3.47E-01	0.6893
IgG1_G1FN	high_IgE	-0.084	0.089	3.47E-01	0.6893
IgG1_G1NS	Wheal sum	-0.005	0.005	3.49E-01	0.6925

IgG1_G1FN	Sensitization II	-0.086	0.093	3.50E-01	0.6925
IgG2_G1	Sensitization I	-0.074	0.079	3.51E-01	0.6943
IgG1_G1FS	pos_birch	0.150	0.163	3.52E-01	0.6944
IgG1_G2F	pos_birch	0.149	0.162	3.53E-01	0.6944
IgG1_G1NS	pos_ambrosia	-0.096	0.105	3.53E-01	0.6944
IgG2_G2FS	mm_tree_mix	0.083	0.092	3.54E-01	0.6944
IgG1_G0FN	mm_hazel	-0.031	0.034	3.57E-01	0.6955
IgG1_G1NS	high_IgE	-0.081	0.089	3.57E-01	0.6955
IgG2_G2FNS	mm_dog	0.036	0.040	3.58E-01	0.6955
IgG1_G0	mm_birch	-0.026	0.028	3.58E-01	0.6955
IgG1_G1	pos_tree_mix	0.343	0.381	3.58E-01	0.6955
IgG1_G0	mm_cladosporium	0.068	0.074	3.58E-01	0.6955
IgG2_G2FNS	mm_tree_mix	0.084	0.093	3.58E-01	0.6955
IgG2_G0	high_IgE	-0.084	0.092	3.59E-01	0.6955
IgG1_G2FNS	mm_ambrosia	0.016	0.018	3.61E-01	0.6970
IgG2_F	mm_cladosporium	-0.067	0.073	3.61E-01	0.6970
IgG1_G1S	mm_dog	-0.035	0.039	3.62E-01	0.6970
IgG1_G2FNS	pos_cat	0.129	0.142	3.62E-01	0.6970
IgG4_G1FNS	mm_dog	-0.035	0.039	3.63E-01	0.6977
IgG2_G1F	pos_cladosporium	-0.258	0.287	3.66E-01	0.6989
IgG2_F	pos_birch	-0.145	0.162	3.66E-01	0.6989
IgG4_N	Sensitization I	-0.075	0.084	3.67E-01	0.6989
IgG1_G1FNS	mm_grass_mix	0.021	0.024	3.68E-01	0.6989
IgG1_G0F	pos_grass_mix	-0.108	0.121	3.69E-01	0.6989
IgG2_N	pos_birch	-0.145	0.163	3.69E-01	0.6989
IgG1_G2	mm_dog	0.035	0.040	3.69E-01	0.6989
IgG1_G2FNS	Sensitization II	0.084	0.095	3.70E-01	0.6989
IgG2_G1FS	mm_hdm	0.017	0.019	3.70E-01	0.6989
IgG1_G0	pos_birch	-0.145	0.163	3.70E-01	0.6989
IgG2_G0FN	mm_hdm	-0.017	0.019	3.71E-01	0.6989
IgG2_S	pos_hdm	0.089	0.100	3.71E-01	0.6989
IgG1_F	pos_birch	-0.141	0.159	3.71E-01	0.6989
IgG1_G2F	Wheal sum	0.005	0.005	3.72E-01	0.6997
IgG2_G1FN	mm_dog	0.034	0.038	3.74E-01	0.7014
IgG2_G2FN	mm_cladosporium	-0.065	0.074	3.74E-01	0.7014
IgG4_G1FS	pos_cat	-0.127	0.144	3.77E-01	0.7046
IgG1_G1F	Sensitization II	-0.079	0.090	3.78E-01	0.7047
IgG1_G2F	mm_birch	0.025	0.028	3.78E-01	0.7047
IgG1_G2FNS	mm_hazel	-0.030	0.034	3.79E-01	0.7047
IgG1_G2FNS	pos_grass_mix	0.104	0.118	3.79E-01	0.7047
IgG4_G0	mm_dog	0.034	0.039	3.80E-01	0.7057
IgG2_S	mm_tree_mix	0.079	0.092	3.85E-01	0.7121
IgG2_G0F	high_IgE	-0.080	0.092	3.85E-01	0.7121
IgG2_N	mm_birch	-0.024	0.028	3.85E-01	0.7121
IgG4_N	mm_cladosporium	-0.062	0.073	3.86E-01	0.7126
IgG1_G2FNS	pos_hdm	0.086	0.100	3.87E-01	0.7128
IgG4_G1F	Wheal sum	-0.004	0.005	3.90E-01	0.7179
IgG1_G0F	pos_cat	-0.123	0.145	3.92E-01	0.7197
IgG1_G1FS	mm_birch	0.024	0.028	3.94E-01	0.7199
IgG2_G0FN	high_IgE	-0.078	0.092	3.94E-01	0.7199

IgG1_G1NS	mm_grass_mix	-0.019	0.023	3.94E-01	0.7199
IgG1_G0	pos_cat	-0.122	0.145	3.95E-01	0.7210
IgG2_G1FS	pos_cat	0.121	0.144	3.96E-01	0.7214
IgG4_G1FN	high_IgE	-0.077	0.092	3.97E-01	0.7217
IgG4_N	mm_tree_mix	0.075	0.091	4.01E-01	0.7277
IgG2_G1S	pos_cladosporium	0.252	0.303	4.01E-01	0.7279
IgG1_G2	Wheal sum	0.005	0.005	4.04E-01	0.7310
IgG2_F	pos_cladosporium	-0.250	0.302	4.05E-01	0.7310
IgG2_N	pos_tree_mix	0.332	0.407	4.05E-01	0.7310
IgG4_G2FS	high_IgE	-0.076	0.092	4.05E-01	0.7310
IgG4_G2FNS	mm_dog	-0.032	0.039	4.08E-01	0.7332
IgG4_G1	pos_cladosporium	-0.236	0.288	4.09E-01	0.7332
IgG4_G0F	mm_dog	0.032	0.039	4.09E-01	0.7332
IgG1_G2	pos_birch	0.132	0.162	4.11E-01	0.7352
IgG2_G1FNS	mm_ambrosia	-0.015	0.018	4.11E-01	0.7352
IgG1_G2F	mm_tree_mix	0.075	0.093	4.12E-01	0.7367
IgG1_G2FNS	mm_hdm	0.015	0.019	4.14E-01	0.7384
IgG2_G1	Sensitization II	-0.072	0.089	4.15E-01	0.7392
IgG1_G1	pos_cat	-0.105	0.130	4.16E-01	0.7392
IgG4_G0FN	mm_cat	0.021	0.026	4.16E-01	0.7392
IgG2_G1	pos_hdm	-0.076	0.094	4.18E-01	0.7404
IgG2_G1F	mm_ambrosia	0.014	0.017	4.18E-01	0.7404
IgG1_G0	mm_ambrosia	-0.014	0.018	4.21E-01	0.7431
IgG2_F	mm_birch	-0.022	0.028	4.21E-01	0.7431
IgG1_G0F	mm_dog	-0.032	0.040	4.22E-01	0.7441
IgG1_G1FS	pos_hazel	0.147	0.186	4.25E-01	0.7469
IgG2_G1F	mm_dog	0.030	0.037	4.25E-01	0.7469
IgG2_G1	mm_ambrosia	0.013	0.017	4.32E-01	0.7580
IgG1_G2FNS	mm_cladosporium	-0.057	0.073	4.33E-01	0.7593
IgG1_G1	mm_tree_mix	0.065	0.085	4.35E-01	0.7603
IgG4_N	pos_dog	-0.133	0.171	4.35E-01	0.7603
IgG2_G1FNS	Wheal sum	-0.004	0.005	4.36E-01	0.7611
IgG1_G2FN	mm_birch	-0.022	0.028	4.37E-01	0.7611
IgG1_G0F	mm_birch	-0.022	0.028	4.39E-01	0.7643
IgG1_G1	mm_hazel	-0.024	0.031	4.40E-01	0.7643
IgG1_G0	pos_grass_mix	-0.092	0.120	4.41E-01	0.7643
IgG1_G1FN	mm_cladosporium	-0.055	0.072	4.42E-01	0.7650
IgG2_G1S	pos_tree_mix	-0.312	0.416	4.44E-01	0.7650
IgG1_G1	mm_grass_mix	0.016	0.021	4.44E-01	0.7650
IgG4_N	Sensitization II	-0.071	0.094	4.45E-01	0.7650
IgG1_G1NS	mm_hazel	-0.025	0.033	4.45E-01	0.7650
IgG1_G2F	mm_hazel	0.026	0.034	4.46E-01	0.7650
IgG1_G2	mm_birch	0.021	0.028	4.46E-01	0.7650
IgG4_G2F	pos_birch	0.121	0.161	4.46E-01	0.7650
IgG2_G1FN	high_IgE	-0.068	0.090	4.48E-01	0.7662
IgG2_G2	mm_cladosporium	-0.056	0.074	4.49E-01	0.7662
IgG2_G1FNS	pos_hazel	-0.139	0.185	4.49E-01	0.7662
IgG1_G1NS	pos_grass_mix	-0.087	0.116	4.50E-01	0.7665
IgG4_G1FS	mm_grass_mix	-0.018	0.024	4.51E-01	0.7676
IgG4_G1NS	pos_cladosporium	-0.226	0.304	4.54E-01	0.7713

IgG1_G1S	mm_birch	0.021	0.028	4.56E-01	0.7733
IgG4_S	pos_birch	0.121	0.164	4.57E-01	0.7733
IgG2_F	pos_grass_mix	-0.088	0.119	4.57E-01	0.7733
IgG1_G0F	pos_birch	-0.119	0.164	4.60E-01	0.7780
IgG1_N	Sensitization II	-0.068	0.094	4.64E-01	0.7809
IgG1_G1F	pos_ambrosia	0.074	0.102	4.64E-01	0.7809
IgG1_N	high_IgE	-0.066	0.091	4.65E-01	0.7809
IgG1_G1FNS	mm_hazel	-0.025	0.034	4.65E-01	0.7809
IgG2_G1NS	Sensitization II	0.069	0.096	4.66E-01	0.7819
IgG1_G2FS	pos_dog	0.121	0.168	4.67E-01	0.7821
IgG2_G2F	Sensitization II	0.069	0.096	4.69E-01	0.7847
IgG2_G1F	pos_hdm	-0.068	0.095	4.70E-01	0.7847
IgG1_G1F	pos_cladosporium	-0.206	0.287	4.71E-01	0.7861
IgG2_N	mm_hdm	-0.013	0.019	4.72E-01	0.7869
IgG4_N	pos_hdm	0.070	0.099	4.76E-01	0.7901
IgG2_G1NS	Wheal sum	0.004	0.005	4.76E-01	0.7901
IgG2_G1F	pos_tree_mix	0.275	0.394	4.76E-01	0.7901
IgG4_G1NS	mm_cladosporium	-0.052	0.074	4.78E-01	0.7908
IgG1_G1FS	pos_tree_mix	0.290	0.417	4.78E-01	0.7908
IgG2_G1S	mm_tree_mix	-0.064	0.093	4.81E-01	0.7922
IgG1_G2FNS	mm_dog	-0.027	0.039	4.81E-01	0.7922
IgG2_G1S	high_IgE	0.064	0.092	4.82E-01	0.7922
IgG4_G2FS	mm_cladosporium	-0.052	0.074	4.83E-01	0.7922
IgG2_G2FNS	mm_grass_mix	-0.016	0.024	4.83E-01	0.7922
IgG2_G2F	mm_cladosporium	-0.052	0.074	4.83E-01	0.7922
IgG2_G1FNS	mm_birch	-0.020	0.028	4.84E-01	0.7922
IgG1_G1FN	Sensitization I	-0.058	0.083	4.84E-01	0.7922
IgG1_G0	pos_ambrosia	-0.075	0.109	4.85E-01	0.7922
IgG1_G1NS	mm_dog	-0.027	0.038	4.85E-01	0.7922
IgG2_G0FN	mm_cat	-0.018	0.026	4.88E-01	0.7955
IgG4_G1FS	pos_hazel	-0.128	0.187	4.89E-01	0.7958
IgG2_G1S	mm_grass_mix	0.016	0.023	4.90E-01	0.7958
IgG4_G1FNS	pos_cat	-0.097	0.142	4.92E-01	0.7982
IgG1_G2FNS	high_IgE	0.062	0.091	4.97E-01	0.8049
IgG2_G1S	pos_grass_mix	0.080	0.119	4.98E-01	0.8060
IgG1_S	pos_dog	0.113	0.169	5.00E-01	0.8064
IgG1_G2FNS	mm_grass_mix	0.016	0.023	5.00E-01	0.8064
IgG1_G1S	pos_grass_mix	0.079	0.118	5.00E-01	0.8064
IgG1_G2FN	pos_birch	-0.108	0.163	5.03E-01	0.8098
IgG4_G2F	mm_hazel	0.022	0.034	5.04E-01	0.8099
IgG1_G1S	pos_ambrosia	0.071	0.107	5.06E-01	0.8121
IgG2_G2FN	high_IgE	-0.061	0.092	5.07E-01	0.8123
IgG4_G1FNS	pos_cladosporium	-0.198	0.301	5.07E-01	0.8123
IgG1_G2	mm_hazel	0.022	0.034	5.09E-01	0.8131
IgG2_G0FN	pos_cladosporium	-0.199	0.303	5.09E-01	0.8131
IgG4_G2FNS	mm_grass_mix	-0.015	0.023	5.12E-01	0.8157
IgG2_G2	Sensitization II	0.062	0.096	5.13E-01	0.8157
IgG2_G1S	mm_cat	0.017	0.026	5.13E-01	0.8157
IgG4_G1FNS	mm_cat	-0.017	0.026	5.18E-01	0.8212
IgG4_G1F	pos_hazel	0.112	0.175	5.18E-01	0.8212

IgG1_G2F	Sensitization I	0.055	0.085	5.19E-01	0.8222
IgG1_G1F	mm_cat	-0.016	0.025	5.20E-01	0.8225
IgG1_G2	Sensitization I	0.055	0.086	5.22E-01	0.8230
IgG1_G1F	pos_grass_mix	0.072	0.113	5.22E-01	0.8230
IgG1_G0	Sensitization I	-0.055	0.086	5.22E-01	0.8230
IgG1_G1	pos_grass_mix	0.069	0.109	5.25E-01	0.8233
IgG4_S	mm_birch	0.018	0.029	5.25E-01	0.8233
IgG1_S	mm_cladosporium	0.045	0.072	5.25E-01	0.8233
IgG4_G0	pos_cat	0.089	0.142	5.26E-01	0.8233
IgG1_G0FN	mm_grass_mix	-0.015	0.023	5.27E-01	0.8233
IgG1_G1NS	Sensitization II	-0.058	0.093	5.27E-01	0.8233
IgG2_G1NS	mm_ambrosia	0.011	0.018	5.29E-01	0.8253
IgG2_G0	mm_hdm	-0.012	0.019	5.30E-01	0.8260
IgG1_N	mm_grass_mix	-0.014	0.023	5.31E-01	0.8260
IgG2_F	pos_hdm	0.062	0.100	5.33E-01	0.8289
IgG4_G1FN	mm_dog	-0.024	0.039	5.35E-01	0.8313
IgG1_G0FN	pos_hdm	0.062	0.101	5.36E-01	0.8319
IgG4_G2F	mm_birch	0.017	0.028	5.38E-01	0.8336
IgG2_G1NS	mm_dog	0.024	0.040	5.39E-01	0.8341
IgG4_G2	Wheal sum	-0.003	0.005	5.40E-01	0.8349
IgG4_G2FS	mm_dog	0.024	0.040	5.42E-01	0.8359
IgG1_G2FS	mm_cladosporium	0.043	0.071	5.42E-01	0.8359
IgG1_F	mm_birch	-0.017	0.028	5.46E-01	0.8395
IgG1_F	mm_hazel	0.020	0.033	5.46E-01	0.8397
IgG1_G2F	pos_cat	0.086	0.144	5.48E-01	0.8416
IgG2_G1NS	mm_tree_mix	-0.054	0.092	5.51E-01	0.8434
IgG4_N	mm_hdm	0.011	0.018	5.52E-01	0.8434
IgG4_S	pos_dog	-0.103	0.175	5.52E-01	0.8434
IgG2_N	mm_cat	0.016	0.026	5.53E-01	0.8434
IgG4_G1FNS	pos_tree_mix	0.238	0.410	5.53E-01	0.8434
IgG2_G2FNS	pos_hdm	0.059	0.101	5.57E-01	0.8481
IgG4_G2FS	pos_hdm	-0.058	0.101	5.62E-01	0.8549
IgG1_G2	pos_cat	0.083	0.144	5.64E-01	0.8552
IgG1_G0FN	pos_hazel	-0.106	0.186	5.64E-01	0.8552
IgG2_G2FN	Sensitization II	-0.055	0.096	5.64E-01	0.8552
IgG2_G1S	pos_hdm	-0.057	0.100	5.68E-01	0.8593
IgG4_N	pos_tree_mix	0.229	0.410	5.69E-01	0.8593
IgG2_G1S	pos_cat	0.081	0.143	5.70E-01	0.8601
IgG4_G1	mm_cladosporium	-0.039	0.070	5.72E-01	0.8601
IgG1_G2F	mm_grass_mix	0.013	0.024	5.73E-01	0.8601
IgG1_G1FN	mm_grass_mix	-0.013	0.023	5.73E-01	0.8601
IgG4_G0F	pos_cat	0.080	0.143	5.73E-01	0.8601
IgG4_N	high_IgE	-0.051	0.091	5.74E-01	0.8601
IgG4_G1NS	mm_tree_mix	0.051	0.092	5.75E-01	0.8601
IgG4_F	mm_tree_mix	-0.050	0.092	5.78E-01	0.8601
IgG1_G1S	mm_ambrosia	0.010	0.018	5.78E-01	0.8601
IgG2_G2FN	pos_grass_mix	0.066	0.120	5.81E-01	0.8601
IgG1_G1NS	mm_cat	0.014	0.026	5.82E-01	0.8601
IgG2_G1F	Sensitization I	-0.044	0.081	5.83E-01	0.8601
IgG2_G1FNS	pos_birch	-0.088	0.162	5.83E-01	0.8601

IgG1_G2	mm_grass_mix	0.013	0.024	5.84E-01	0.8601
IgG1_G0FN	pos_cat	-0.078	0.144	5.84E-01	0.8601
IgG2_G2FNS	pos_hazel	-0.100	0.185	5.84E-01	0.8601
IgG4_G1FS	pos_hdm	0.055	0.101	5.85E-01	0.8601
IgG4_G1FS	mm_hdm	0.010	0.019	5.85E-01	0.8601
IgG2_G1	high_IgE	-0.046	0.086	5.85E-01	0.8601
IgG4_G1S	mm_tree_mix	0.049	0.092	5.85E-01	0.8601
IgG1_G0FN	mm_cat	-0.014	0.027	5.86E-01	0.8601
IgG1_G1S	mm_cat	0.014	0.026	5.87E-01	0.8601
IgG4_G2	pos_hazel	-0.099	0.184	5.87E-01	0.8601
IgG1_G0FN	Sensitization II	-0.051	0.095	5.87E-01	0.8601
IgG1_F	mm_cat	-0.014	0.026	5.90E-01	0.8601
IgG1_G1S	pos_cat	0.076	0.142	5.90E-01	0.8601
IgG2_G1NS	pos_grass_mix	0.064	0.120	5.90E-01	0.8601
IgG1_G1	pos_birch	-0.079	0.148	5.91E-01	0.8601
IgG4_G1F	mm_hazel	0.017	0.032	5.93E-01	0.8601
IgG1_G1F	pos_cat	-0.072	0.136	5.93E-01	0.8601
IgG1_G1FN	mm_cat	0.014	0.026	5.94E-01	0.8601
IgG2_G1FN	pos_hazel	-0.097	0.184	5.94E-01	0.8601
IgG2_G1	pos_birch	-0.080	0.151	5.94E-01	0.8601
IgG4_G1	mm_ambrosia	-0.009	0.017	5.95E-01	0.8601
IgG1_G1F	high_IgE	-0.046	0.087	5.96E-01	0.8601
IgG2_G0FN	pos_cat	-0.076	0.143	5.96E-01	0.8601
IgG1_G2FNS	Wheal sum	0.003	0.005	5.96E-01	0.8601
IgG1_S	pos_cladosporium	0.155	0.295	5.97E-01	0.8601
IgG2_G1FNS	high_IgE	-0.048	0.092	5.97E-01	0.8601
IgG4_G0	pos_birch	-0.083	0.160	5.98E-01	0.8602
IgG2_G1F	pos_cat	-0.071	0.136	5.99E-01	0.8606
IgG2_G1FNS	pos_ambrosia	-0.056	0.108	6.02E-01	0.8642
IgG2_G1S	pos_birch	0.083	0.162	6.03E-01	0.8643
IgG2_F	mm_hdm	0.010	0.019	6.04E-01	0.8643
IgG1_N	Sensitization I	-0.043	0.084	6.11E-01	0.8716
IgG1_G1	Wheal sum	-0.003	0.005	6.11E-01	0.8716
IgG2_G0FN	pos_tree_mix	-0.204	0.411	6.12E-01	0.8716
IgG1_N	pos_hdm	0.050	0.100	6.13E-01	0.8716
IgG4_G1	pos_birch	-0.076	0.151	6.13E-01	0.8716
IgG1_G1NS	mm_hdm	-0.009	0.018	6.14E-01	0.8716
IgG1_G0FN	Sensitization I	-0.043	0.085	6.15E-01	0.8716
IgG4_G1NS	pos_hdm	0.050	0.101	6.15E-01	0.8716
IgG4_G0	pos_hazel	0.090	0.182	6.16E-01	0.8716
IgG1_G0F	Sensitization I	-0.043	0.086	6.17E-01	0.8716
IgG2_G1FNS	mm_hazel	-0.017	0.034	6.17E-01	0.8716
IgG4_S	pos_hdm	-0.050	0.102	6.18E-01	0.8716
IgG4_G1FN	pos_cat	-0.071	0.144	6.20E-01	0.8734
IgG4_S	mm_cat	0.013	0.027	6.21E-01	0.8747
IgG1_G1FNS	Wheal sum	0.003	0.005	6.25E-01	0.8782
IgG4_G2	pos_birch	0.078	0.162	6.27E-01	0.8801
IgG2_G0F	mm_hdm	-0.009	0.019	6.28E-01	0.8804
IgG2_G0FN	mm_tree_mix	-0.043	0.092	6.29E-01	0.8809
IgG4_S	mm_hdm	-0.009	0.019	6.31E-01	0.8821

IgG1_G1FNS	pos_cladosporium	-0.146	0.305	6.31E-01	0.8821
IgG4_G2FNS	pos_cat	-0.068	0.143	6.33E-01	0.8834
IgG2_G1FNS	mm_dog	0.019	0.040	6.35E-01	0.8849
IgG4_G1	mm_birch	-0.012	0.026	6.35E-01	0.8849
IgG2_G0F	pos_cladosporium	0.142	0.305	6.38E-01	0.8881
IgG4_G1	mm_grass_mix	-0.010	0.022	6.40E-01	0.8891
IgG4_G1FS	mm_tree_mix	0.042	0.093	6.42E-01	0.8909
IgG1_G2FS	pos_cladosporium	0.135	0.293	6.43E-01	0.8912
IgG1_G1NS	pos_hazel	-0.083	0.181	6.44E-01	0.8912
IgG4_G1FN	pos_hdm	0.046	0.101	6.48E-01	0.8953
IgG4_G1FNS	mm_cladosporium	-0.033	0.073	6.48E-01	0.8953
IgG4_G0	mm_cat	0.012	0.026	6.50E-01	0.8970
IgG1_G1S	Sensitization I	0.037	0.084	6.55E-01	0.9019
IgG4_G2FS	mm_hdm	-0.008	0.019	6.56E-01	0.9019
IgG1_F	mm_tree_mix	-0.039	0.090	6.56E-01	0.9019
IgG1_G0F	mm_ambrosia	-0.008	0.018	6.57E-01	0.9019
IgG4_G1FN	pos_grass_mix	0.052	0.120	6.59E-01	0.9019
IgG2_G1NS	pos_tree_mix	-0.179	0.413	6.59E-01	0.9019
IgG2_G2FN	Sensitization I	-0.037	0.085	6.59E-01	0.9019
IgG1_G1S	mm_grass_mix	0.010	0.023	6.61E-01	0.9029
IgG2_F	high_IgE	-0.040	0.092	6.61E-01	0.9029
IgG2_N	pos_dog	-0.075	0.173	6.63E-01	0.9033
IgG1_F	pos_cat	-0.061	0.141	6.64E-01	0.9033
IgG1_G1F	mm_birch	0.012	0.027	6.64E-01	0.9033
IgG2_G1F	Sensitization II	-0.039	0.090	6.66E-01	0.9040
IgG2_G1FN	Wheal sum	0.002	0.005	6.66E-01	0.9040
IgG4_G1NS	mm_hdm	0.008	0.019	6.67E-01	0.9040
IgG4_G1F	mm_tree_mix	-0.038	0.090	6.68E-01	0.9043
IgG2_G1FS	pos_hdm	0.043	0.101	6.69E-01	0.9050
IgG1_G1FNS	pos_cat	0.061	0.144	6.70E-01	0.9053
IgG1_G2FN	mm_ambrosia	-0.008	0.018	6.71E-01	0.9053
IgG1_G0	pos_hdm	0.043	0.102	6.72E-01	0.9057
IgG4_G2F	Wheal sum	-0.002	0.005	6.73E-01	0.9060
IgG2_G1	pos_dog	0.068	0.162	6.74E-01	0.9068
IgG4_G0FN	pos_grass_mix	0.048	0.116	6.75E-01	0.9072
IgG4_G0FN	pos_cat	0.058	0.140	6.78E-01	0.9093
IgG2_G1FNS	pos_grass_mix	-0.050	0.120	6.78E-01	0.9093
IgG2_G1F	pos_dog	0.068	0.164	6.79E-01	0.9093
IgG2_G2FNS	pos_grass_mix	-0.049	0.120	6.83E-01	0.9097
IgG1_G1	mm_cat	-0.010	0.024	6.83E-01	0.9097
IgG2_G2F	high_IgE	0.037	0.093	6.84E-01	0.9097
IgG1_G0FN	high_IgE	-0.037	0.092	6.84E-01	0.9097
IgG4_G1F	pos_tree_mix	-0.161	0.403	6.84E-01	0.9097
IgG2_G2FN	mm_birch	0.011	0.028	6.85E-01	0.9097
IgG4_G1	pos_ambrosia	-0.041	0.102	6.86E-01	0.9097
IgG2_G1S	mm_birch	0.011	0.028	6.86E-01	0.9097
IgG1_F	high_IgE	0.036	0.090	6.87E-01	0.9097
IgG1_G0F	pos_hdm	0.041	0.102	6.87E-01	0.9097
IgG2_G0FN	mm_cladosporium	-0.029	0.074	6.90E-01	0.9097
IgG4_G2FS	Sensitization II	-0.038	0.096	6.90E-01	0.9097

IgG2_G1	mm_hdm	-0.007	0.017	6.91E-01	0.9097
IgG4_G1F	mm_grass_mix	-0.009	0.022	6.92E-01	0.9097
IgG1_G1FNS	mm_ambrosia	0.007	0.018	6.92E-01	0.9097
IgG2_G2FN	mm_grass_mix	0.009	0.023	6.93E-01	0.9097
IgG4_S	pos_hazel	-0.073	0.188	6.94E-01	0.9097
IgG1_G1S	mm_hazel	-0.013	0.034	6.94E-01	0.9097
IgG1_G0	mm_hazel	-0.013	0.034	6.95E-01	0.9097
IgG1_N	pos_cat	-0.055	0.142	6.95E-01	0.9097
IgG1_G1	mm_ambrosia	0.006	0.016	6.97E-01	0.9097
IgG1_G0F	pos_ambrosia	-0.042	0.109	6.97E-01	0.9097
IgG2_G1F	pos_birch	-0.059	0.154	6.98E-01	0.9097
IgG2_G1FNS	mm_cat	-0.010	0.027	6.98E-01	0.9097
IgG1_G1FN	pos_cat	-0.054	0.140	6.99E-01	0.9097
IgG4_G1	mm_tree_mix	0.034	0.089	7.00E-01	0.9097
IgG2_F	mm_hazel	-0.013	0.034	7.00E-01	0.9097
IgG2_F	Sensitization II	-0.036	0.095	7.02E-01	0.9097
IgG4_G0FN	pos_ambrosia	0.040	0.105	7.02E-01	0.9097
IgG2_G1FN	mm_hazel	-0.013	0.034	7.02E-01	0.9097
IgG2_G1NS	pos_cladosporium	0.115	0.305	7.03E-01	0.9097
IgG2_G1FNS	pos_tree_mix	-0.156	0.420	7.05E-01	0.9102
IgG1_G2F	pos_grass_mix	0.045	0.120	7.06E-01	0.9102
IgG4_G2	mm_hazel	0.013	0.034	7.06E-01	0.9102
IgG1_G2F	pos_dog	0.065	0.174	7.07E-01	0.9102
IgG1_F	pos_tree_mix	-0.148	0.403	7.08E-01	0.9102
IgG1_G2	pos_grass_mix	0.045	0.120	7.09E-01	0.9102
IgG1_G2FN	high_IgE	-0.034	0.092	7.09E-01	0.9102
IgG4_N	pos_cat	-0.052	0.141	7.09E-01	0.9102
IgG1_N	mm_cladosporium	-0.027	0.073	7.11E-01	0.9118
IgG2_G1S	Wheal sum	0.002	0.005	7.13E-01	0.9126
IgG2_G1FN	pos_birch	-0.058	0.161	7.15E-01	0.9129
IgG2_G1NS	mm_hdm	-0.007	0.019	7.15E-01	0.9129
IgG1_G2FNS	pos_hazel	-0.067	0.186	7.18E-01	0.9153
IgG1_G2F	pos_hazel	0.066	0.185	7.19E-01	0.9153
IgG4_G0FN	pos_tree_mix	-0.142	0.405	7.20E-01	0.9153
IgG4_G2	mm_birch	0.010	0.028	7.21E-01	0.9153
IgG4_G0FN	mm_cladosporium	0.026	0.072	7.21E-01	0.9153
IgG2_F	Wheal sum	-0.002	0.005	7.23E-01	0.9153
IgG4_S	pos_cat	0.051	0.145	7.24E-01	0.9153
IgG4_N	pos_grass_mix	-0.041	0.118	7.24E-01	0.9153
IgG2_F	mm_grass_mix	-0.008	0.023	7.25E-01	0.9153
IgG1_G0FN	pos_cladosporium	-0.106	0.304	7.25E-01	0.9153
IgG2_G1S	Sensitization II	0.033	0.095	7.25E-01	0.9153
IgG2_G1F	mm_hazel	0.011	0.032	7.28E-01	0.9186
IgG2_G0	pos_cladosporium	0.104	0.305	7.31E-01	0.9195
IgG4_G1	pos_tree_mix	0.134	0.399	7.32E-01	0.9195
IgG4_G0FN	mm_dog	0.013	0.038	7.32E-01	0.9195
IgG1_G2FNS	mm_birch	-0.010	0.028	7.33E-01	0.9195
IgG2_G1NS	mm_cladosporium	0.025	0.074	7.33E-01	0.9195
IgG1_G1FS	mm_hazel	0.012	0.034	7.35E-01	0.9208
IgG2_G1S	Sensitization I	-0.028	0.085	7.37E-01	0.9228

IgG2_G1F	mm_hdm	-0.006	0.018	7.42E-01	0.9274
IgG4_G2	pos_dog	-0.056	0.173	7.44E-01	0.9291
IgG2_G1NS	high_IgE	0.029	0.092	7.50E-01	0.9356
IgG2_N	pos_cat	0.045	0.143	7.51E-01	0.9356
IgG1_G2FN	Wheal sum	-0.002	0.005	7.51E-01	0.9356
IgG2_F	pos_cat	-0.045	0.143	7.52E-01	0.9356
IgG4_G2FN	pos_cat	-0.045	0.145	7.53E-01	0.9356
IgG2_G2FN	mm_hazel	0.011	0.034	7.56E-01	0.9356
IgG4_G0F	mm_hazel	0.010	0.034	7.56E-01	0.9356
IgG1_G1S	Wheal sum	0.002	0.005	7.57E-01	0.9356
IgG1_G2F	high_IgE	-0.028	0.092	7.57E-01	0.9356
IgG4_N	mm_grass_mix	-0.007	0.023	7.58E-01	0.9356
IgG4_G0	mm_birch	-0.009	0.028	7.59E-01	0.9356
IgG1_G0FN	mm_hdm	0.006	0.019	7.60E-01	0.9356
IgG2_G2	high_IgE	0.028	0.093	7.61E-01	0.9356
IgG1_F	pos_hazel	0.055	0.182	7.61E-01	0.9356
IgG4_S	Wheal sum	-0.002	0.005	7.61E-01	0.9356
IgG4_G0FN	mm_ambrosia	0.005	0.017	7.62E-01	0.9356
IgG1_G0F	mm_hdm	0.006	0.019	7.62E-01	0.9356
IgG1_G2	high_IgE	-0.028	0.092	7.63E-01	0.9356
IgG2_F	mm_cat	-0.008	0.026	7.64E-01	0.9356
IgG1_G0FN	mm_cladosporium	0.022	0.074	7.66E-01	0.9378
IgG2_G1S	pos_hazel	-0.054	0.185	7.67E-01	0.9380
IgG1_G1FNS	pos_hazel	-0.054	0.187	7.69E-01	0.9389
IgG1_G1NS	pos_cat	0.040	0.139	7.72E-01	0.9412
IgG2_G2FNS	high_IgE	-0.026	0.092	7.73E-01	0.9420
IgG1_G0	Sensitization II	-0.027	0.096	7.74E-01	0.9421
IgG1_F	Sensitization I	-0.024	0.083	7.75E-01	0.9421
IgG1_G1NS	pos_hdm	-0.028	0.098	7.76E-01	0.9421
IgG2_G1S	mm_hdm	-0.005	0.019	7.78E-01	0.9422
IgG1_F	pos_grass_mix	-0.033	0.117	7.78E-01	0.9422
IgG2_G2F	mm_hdm	0.005	0.019	7.79E-01	0.9422
IgG2_G0FN	pos_hdm	-0.028	0.101	7.80E-01	0.9422
IgG4_G0FN	mm_grass_mix	0.006	0.023	7.80E-01	0.9422
IgG4_G2FN	mm_dog	-0.011	0.040	7.80E-01	0.9422
IgG1_G2	pos_hazel	0.051	0.185	7.82E-01	0.9427
IgG4_G2FNS	mm_cat	-0.007	0.026	7.83E-01	0.9427
IgG2_G2FN	pos_birch	0.044	0.163	7.84E-01	0.9427
IgG1_G0	mm_hdm	0.005	0.019	7.85E-01	0.9427
IgG2_G1	mm_cat	0.007	0.025	7.86E-01	0.9427
IgG2_F	pos_ambrosia	-0.029	0.108	7.86E-01	0.9427
IgG2_G2FNS	pos_ambrosia	-0.029	0.108	7.89E-01	0.9454
IgG2_G1F	mm_grass_mix	-0.006	0.022	7.90E-01	0.9457
IgG2_G2	mm_hdm	0.005	0.019	7.90E-01	0.9457
IgG4_G0	pos_grass_mix	0.031	0.118	7.92E-01	0.9463
IgG1_G1F	pos_hazel	-0.046	0.177	7.93E-01	0.9463
IgG2_G2FNS	Sensitization II	-0.025	0.096	7.95E-01	0.9470
IgG2_G1F	mm_cat	-0.006	0.025	7.96E-01	0.9470
IgG1_G2FN	pos_cat	0.037	0.143	7.97E-01	0.9470
IgG2_G1	mm_grass_mix	-0.006	0.022	7.97E-01	0.9470

IgG1_G0FN	mm_tree_mix	0.023	0.092	7.98E-01	0.9470
IgG1_N	mm_cat	0.007	0.026	7.99E-01	0.9470
IgG4_S	mm_grass_mix	0.006	0.024	7.99E-01	0.9470
IgG1_G1FNS	pos_birch	0.041	0.164	7.99E-01	0.9470
IgG1_G0FN	pos_tree_mix	-0.101	0.411	8.02E-01	0.9490
IgG2_N	pos_hdm	-0.025	0.101	8.04E-01	0.9505
IgG2_G1F	pos_grass_mix	-0.027	0.113	8.08E-01	0.9545
IgG2_G1FNS	mm_hdm	-0.005	0.019	8.09E-01	0.9545
IgG1_G1	pos_ambrosia	0.023	0.098	8.10E-01	0.9552
IgG2_G1	pos_cat	-0.031	0.134	8.13E-01	0.9566
IgG2_G2FN	pos_hdm	0.024	0.101	8.14E-01	0.9566
IgG4_G1FNS	mm_hdm	0.004	0.019	8.14E-01	0.9566
IgG4_G1FS	pos_tree_mix	0.095	0.418	8.17E-01	0.9589
IgG4_F	pos_hdm	-0.023	0.101	8.20E-01	0.9602
IgG1_G2FN	pos_ambrosia	-0.024	0.108	8.20E-01	0.9602
IgG2_G1	Wheal sum	0.001	0.005	8.20E-01	0.9602
IgG4_G2FNS	pos_hdm	0.023	0.101	8.21E-01	0.9603
IgG2_G1FNS	Sensitization II	-0.021	0.096	8.23E-01	0.9604
IgG2_G2FNS	mm_hdm	0.004	0.019	8.23E-01	0.9604
IgG4_G2F	mm_cat	0.006	0.026	8.25E-01	0.9604
IgG4_G0FN	mm_tree_mix	-0.020	0.090	8.25E-01	0.9604
IgG2_G1FN	pos_grass_mix	0.025	0.117	8.26E-01	0.9604
IgG4_G2	mm_cat	0.006	0.026	8.26E-01	0.9604
IgG1_G2FN	pos_hdm	0.022	0.101	8.28E-01	0.9611
IgG1_G1FN	mm_hdm	-0.004	0.018	8.28E-01	0.9611
IgG2_N	mm_dog	-0.008	0.039	8.29E-01	0.9611
IgG2_G2FN	mm_hdm	0.004	0.019	8.30E-01	0.9615
IgG1_G0F	mm_hazel	-0.007	0.034	8.31E-01	0.9615
IgG1_G2F	mm_ambrosia	0.004	0.018	8.33E-01	0.9621
IgG4_G1FN	mm_cat	0.006	0.027	8.35E-01	0.9632
IgG4_G0F	mm_cat	0.006	0.027	8.35E-01	0.9632
IgG4_G1FS	pos_cladosporium	0.060	0.306	8.42E-01	0.9663
IgG2_G1F	high_IgE	-0.017	0.087	8.42E-01	0.9663
IgG1_G1F	Wheal sum	0.001	0.005	8.43E-01	0.9663
IgG2_G1FNS	pos_hdm	0.020	0.101	8.45E-01	0.9663
IgG1_G0	pos_dog	-0.034	0.175	8.45E-01	0.9663
IgG2_G1	pos_hazel	-0.033	0.172	8.45E-01	0.9663
IgG4_N	mm_dog	-0.008	0.039	8.46E-01	0.9663
IgG1_G0F	high_IgE	0.018	0.093	8.48E-01	0.9663
IgG1_G2	mm_ambrosia	0.003	0.018	8.50E-01	0.9663
IgG4_G1FN	mm_hdm	0.004	0.019	8.50E-01	0.9663
IgG1_G1	mm_birch	-0.005	0.026	8.51E-01	0.9663
IgG4_G2FS	pos_ambrosia	-0.020	0.108	8.52E-01	0.9663
IgG1_G0F	pos_hazel	0.035	0.187	8.52E-01	0.9663
IgG2_G1F	Wheal sum	0.001	0.005	8.52E-01	0.9663
IgG2_G1	pos_grass_mix	-0.021	0.111	8.52E-01	0.9663
IgG1_G1	pos_dog	-0.029	0.158	8.53E-01	0.9663
IgG2_F	pos_hazel	-0.034	0.185	8.53E-01	0.9663
IgG4_F	mm_hdm	-0.004	0.019	8.53E-01	0.9663
IgG4_G0F	pos_grass_mix	0.022	0.119	8.53E-01	0.9663

IgG2_G2FNS	Sensitization I	-0.015	0.086	8.56E-01	0.9679
IgG1_F	pos_hdm	0.018	0.099	8.56E-01	0.9679
IgG1_G2	pos_dog	0.031	0.175	8.58E-01	0.9679
IgG1_G2FN	pos_grass_mix	0.021	0.119	8.58E-01	0.9679
IgG4_G2F	pos_dog	-0.030	0.172	8.59E-01	0.9679
IgG1_G2FN	Sensitization II	-0.017	0.095	8.60E-01	0.9688
IgG2_G2FNS	mm_hazel	-0.006	0.034	8.63E-01	0.9705
IgG1_G1NS	Sensitization I	-0.014	0.083	8.65E-01	0.9705
IgG2_G0	pos_hdm	-0.017	0.101	8.66E-01	0.9705
IgG1_F	pos_ambrosia	-0.018	0.106	8.68E-01	0.9705
IgG4_G0	mm_hazel	-0.006	0.034	8.68E-01	0.9705
IgG1_G2FNS	pos_birch	0.027	0.163	8.68E-01	0.9705
IgG4_G2F	pos_hazel	-0.030	0.184	8.68E-01	0.9705
IgG2_G1FN	mm_ambrosia	0.003	0.017	8.68E-01	0.9705
IgG2_G2FN	pos_ambrosia	-0.018	0.108	8.70E-01	0.9712
IgG2_G2FNS	mm_birch	-0.004	0.028	8.76E-01	0.9768
IgG2_G1FNS	mm_cladosporium	-0.011	0.074	8.77E-01	0.9772
IgG1_G2	Sensitization II	0.015	0.096	8.79E-01	0.9772
IgG4_G2FN	mm_cat	0.004	0.027	8.80E-01	0.9772
IgG4_G1F	pos_ambrosia	0.016	0.103	8.80E-01	0.9772
IgG1_G1S	mm_tree_mix	0.014	0.091	8.80E-01	0.9772
IgG1_N	mm_hdm	0.003	0.019	8.83E-01	0.9795
IgG4_S	pos_grass_mix	-0.017	0.121	8.85E-01	0.9795
IgG1_G2F	Sensitization II	0.014	0.096	8.85E-01	0.9795
IgG4_G2F	mm_dog	0.006	0.039	8.87E-01	0.9795
IgG1_F	Sensitization II	0.013	0.093	8.88E-01	0.9795
IgG4_G2FN	pos_grass_mix	-0.017	0.121	8.88E-01	0.9795
IgG2_G2FNS	pos_birch	-0.022	0.162	8.89E-01	0.9795
IgG2_G1NS	pos_cat	0.020	0.144	8.89E-01	0.9795
IgG2_G1FNS	mm_tree_mix	-0.013	0.093	8.90E-01	0.9801
IgG1_F	Wheal sum	0.001	0.005	8.94E-01	0.9803
IgG2_G0F	mm_cladosporium	-0.010	0.074	8.94E-01	0.9803
IgG2_G1F	pos_ambrosia	0.013	0.102	8.95E-01	0.9803
IgG4_G2FN	pos_hdm	0.013	0.102	8.95E-01	0.9803
IgG2_G1NS	pos_hdm	0.013	0.101	8.96E-01	0.9803
IgG2_G1FNS	Sensitization I	0.011	0.086	8.98E-01	0.9803
IgG4_G1F	pos_grass_mix	-0.014	0.114	8.99E-01	0.9803
IgG1_G1S	pos_hdm	-0.013	0.100	8.99E-01	0.9803
IgG4_G1S	mm_hdm	-0.002	0.019	9.00E-01	0.9803
IgG4_G1FN	mm_grass_mix	0.003	0.023	9.00E-01	0.9803
IgG2_G1FN	mm_birch	-0.003	0.028	9.01E-01	0.9803
IgG4_G0FN	pos_dog	0.021	0.169	9.01E-01	0.9803
IgG1_G1S	mm_hdm	0.002	0.019	9.04E-01	0.9803
IgG1_G1S	high_IgE	-0.011	0.091	9.05E-01	0.9803
IgG2_G1	pos_ambrosia	0.012	0.101	9.05E-01	0.9803
IgG2_G1	mm_hazel	0.004	0.032	9.07E-01	0.9803
IgG4_G2	mm_dog	0.005	0.039	9.08E-01	0.9803
IgG1_G0F	Sensitization II	-0.011	0.096	9.09E-01	0.9803
IgG1_G1S	Sensitization II	0.011	0.094	9.09E-01	0.9803
IgG1_G1FN	pos_hdm	0.011	0.098	9.10E-01	0.9803

IgG1_G1F	pos_birch	0.017	0.154	9.10E-01	0.9803
IgG4_G2FNS	mm_hdm	-0.002	0.019	9.10E-01	0.9803
IgG4_G1S	pos_hdm	-0.011	0.101	9.10E-01	0.9803
IgG2_G1S	pos_ambrosia	0.012	0.108	9.11E-01	0.9803
IgG2_G0	mm_cladosporium	-0.008	0.074	9.12E-01	0.9803
IgG1_G0F	pos_dog	0.019	0.175	9.12E-01	0.9803
IgG2_G2FNS	Wheal sum	0.001	0.005	9.13E-01	0.9803
IgG2_G1NS	mm_grass_mix	-0.003	0.024	9.14E-01	0.9812
IgG4_G0FN	Wheal sum	0.001	0.005	9.15E-01	0.9814
IgG1_G2FN	mm_hdm	0.002	0.019	9.16E-01	0.9815
IgG2_G2FN	pos_hazel	-0.019	0.186	9.17E-01	0.9816
IgG1_G1NS	pos_birch	0.016	0.159	9.20E-01	0.9835
IgG4_G0	mm_grass_mix	0.002	0.023	9.21E-01	0.9835
IgG2_G0F	pos_hdm	-0.010	0.101	9.22E-01	0.9844
IgG4_G2F	mm_grass_mix	0.002	0.023	9.24E-01	0.9849
IgG1_G1	mm_dog	0.003	0.036	9.27E-01	0.9854
IgG1_G2F	mm_hdm	-0.002	0.019	9.27E-01	0.9854
IgG2_G1FN	mm_hdm	0.002	0.018	9.28E-01	0.9854
IgG4_F	pos_tree_mix	0.036	0.414	9.29E-01	0.9854
IgG2_G2	pos_hdm	-0.009	0.102	9.29E-01	0.9854
IgG2_G1FNS	pos_cladosporium	-0.027	0.305	9.29E-01	0.9854
IgG1_F	mm_hdm	0.002	0.018	9.30E-01	0.9854
IgG4_G1F	mm_cladosporium	-0.006	0.071	9.31E-01	0.9854
IgG1_G1S	pos_hazel	-0.015	0.183	9.32E-01	0.9863
IgG4_S	mm_hazel	0.003	0.034	9.34E-01	0.9865
IgG1_G1F	pos_tree_mix	0.032	0.394	9.34E-01	0.9865
IgG2_G2F	pos_hdm	-0.008	0.102	9.36E-01	0.9866
IgG4_G2FN	mm_hdm	0.002	0.019	9.36E-01	0.9866
IgG1_F	mm_grass_mix	-0.002	0.023	9.39E-01	0.9866
IgG1_G1FNS	mm_cladosporium	0.006	0.074	9.40E-01	0.9866
IgG1_G2	mm_hdm	-0.001	0.019	9.40E-01	0.9866
IgG2_G2FN	mm_ambrosia	0.001	0.018	9.40E-01	0.9866
IgG4_G2F	pos_grass_mix	0.009	0.119	9.41E-01	0.9866
IgG4_S	mm_dog	0.003	0.040	9.41E-01	0.9866
IgG4_G2	pos_cat	-0.010	0.143	9.43E-01	0.9880
IgG4_G1F	pos_birch	0.011	0.153	9.44E-01	0.9880
IgG4_G2FS	mm_ambrosia	-0.001	0.018	9.45E-01	0.9880
IgG2_G1F	pos_hazel	0.011	0.175	9.48E-01	0.9906
IgG4_G1NS	pos_tree_mix	-0.026	0.415	9.50E-01	0.9906
IgG1_G1NS	mm_birch	0.002	0.028	9.51E-01	0.9906
IgG4_G1FNS	pos_hdm	0.006	0.100	9.51E-01	0.9906
IgG2_G1F	mm_birch	0.002	0.027	9.52E-01	0.9910
IgG1_G1F	mm_hazel	0.002	0.032	9.53E-01	0.9910
IgG1_G0	high_IgE	0.005	0.093	9.54E-01	0.9912
IgG2_G1FNS	pos_cat	-0.008	0.144	9.54E-01	0.9912
IgG4_G0F	mm_birch	0.002	0.028	9.57E-01	0.9929
IgG1_G2FN	mm_grass_mix	-0.001	0.023	9.60E-01	0.9944
IgG2_G1S	mm_ambrosia	-0.001	0.018	9.60E-01	0.9944
IgG1_G1F	mm_tree_mix	-0.004	0.088	9.63E-01	0.9946
IgG1_F	mm_ambrosia	0.001	0.017	9.65E-01	0.9946

IgG4_G2	pos_grass_mix	-0.005	0.119	9.66E-01	0.9946
IgG4_G0F	pos_birch	-0.007	0.162	9.66E-01	0.9946
IgG4_N	mm_cat	0.001	0.026	9.67E-01	0.9946
IgG2_G1NS	mm_cat	0.001	0.027	9.68E-01	0.9946
IgG4_G2FN	mm_grass_mix	-0.001	0.024	9.71E-01	0.9946
IgG4_G1	pos_hazel	-0.006	0.173	9.71E-01	0.9946
IgG4_G1	pos_grass_mix	-0.004	0.113	9.72E-01	0.9946
IgG1_G0	pos_hazel	0.006	0.187	9.73E-01	0.9946
IgG1_N	pos_grass_mix	-0.004	0.118	9.73E-01	0.9946
IgG1_G2F	pos_ambrosia	-0.003	0.108	9.78E-01	0.9946
IgG2_G1S	mm_hazel	-0.001	0.034	9.78E-01	0.9946
IgG4_G2FS	Sensitization I	0.002	0.086	9.78E-01	0.9946
IgG1_G0FN	pos_grass_mix	-0.003	0.120	9.78E-01	0.9946
IgG2_F	mm_ambrosia	0.001	0.018	9.78E-01	0.9946
IgG4_G0F	mm_grass_mix	0.001	0.023	9.79E-01	0.9946
IgG4_G1F	pos_cladosporium	0.007	0.291	9.80E-01	0.9946
IgG2_G1FN	pos_dog	0.004	0.169	9.80E-01	0.9946
IgG4_G1S	pos_tree_mix	-0.010	0.413	9.81E-01	0.9946
IgG2_G2FNS	mm_ambrosia	0.000	0.018	9.82E-01	0.9946
IgG2_F	Sensitization I	-0.002	0.085	9.82E-01	0.9946
IgG1_G2F	pos_hdm	-0.002	0.101	9.82E-01	0.9946
IgG4_G1FS	mm_cladosporium	-0.002	0.074	9.83E-01	0.9946
IgG4_G1F	mm_birch	-0.001	0.027	9.83E-01	0.9946
IgG2_G1FN	pos_hdm	0.002	0.098	9.84E-01	0.9946
IgG4_G0FN	pos_cladosporium	-0.006	0.296	9.84E-01	0.9946
IgG1_G1FN	pos_grass_mix	-0.002	0.116	9.85E-01	0.9946
IgG1_G1S	pos_tree_mix	0.007	0.411	9.86E-01	0.9946
IgG2_G1	mm_birch	0.000	0.026	9.86E-01	0.9946
IgG4_G2	mm_grass_mix	0.000	0.023	9.86E-01	0.9946
IgG4_G1F	mm_ambrosia	0.000	0.017	9.88E-01	0.9946
IgG1_G2FN	Sensitization I	-0.001	0.085	9.89E-01	0.9946
IgG2_G1FN	mm_grass_mix	0.000	0.023	9.89E-01	0.9946
IgG1_G2	pos_ambrosia	-0.001	0.109	9.92E-01	0.9961
IgG4_G1	mm_hazel	0.000	0.032	9.92E-01	0.9961
IgG4_G2F	pos_cat	0.001	0.142	9.95E-01	0.9981
IgG4_G2FS	pos_dog	0.001	0.174	9.96E-01	0.9981
IgG2_G1FN	pos_ambrosia	0.000	0.105	9.97E-01	0.9983
IgG1_G1FNS	mm_birch	0.000	0.029	9.98E-01	0.9983
IgG1_G2	pos_hdm	0.000	0.101	9.98E-01	0.9983

**pos_tree_mix**	positive SPT (mean wheal diameter >= 3 mm) to trees mix allergens
**pos_birch**	positive SPT (mean wheal diameter >= 3 mm) to birch allergen
**pos_hazel**	positive SPT (mean wheal diameter >= 3 mm) to hazel allergen
**pos_grass_mix**	positive SPT (mean wheal diameter >= 3 mm) to grass mix allergens
**pos_ambrosia**	positive SPT (mean wheal diameter >= 3 mm) to ambrosia allergen
**pos_dog**	positive SPT (mean wheal diameter >= 3 mm) to dog allergen
**pos_cat**	positive SPT (mean wheal diameter >= 3 mm) to cat allergen
**pos_hdm**	positive SPT (mean wheal diameter >= 3 mm) to house dust mite allergen
**pos_cladosporium**	positive SPT (mean wheal diameter >= 3 mm) to cladosporium allergens
**mm_tree_mix**	mean wheal diameter to trees mix allergens
**mm_birch**	mean wheal diameter to birch allergen
**mm_hazel**	mean wheal diameter to hazel allergen
**mm_grass_mix**	mean wheal diameter to grass mix allergens
**mm_ambrosia**	mean wheal diameter to ambrosia allergen
**mm_dog**	mean wheal diameter to dog allergen
**mm_cat**	mean wheal diameter to cat allergen
**mm_hdm**	mean wheal diameter to house dust mite allergen
**mm_cladosporium**	mean wheal diameter to cladosporium allergens
**Sensitization I**	positive SPT to any allergen
**Wheal sum**	positive wheal diameter sum
**high IgE**	serum IgE level above the referent value
**Sensitization II**	positive SPT to any allergen AND IgE level above the referent value
**G0**	agalactosylated glycopeptides
**G1**	monogalactosylated glycopeptides
**G2**	digalactosylated glycopeptides
**F**	core fucosylated glycopeptides
**S**	sialylated glycopeptides
**N**	glycopeptides bearing a bisecting *N*- acetylglucosamine
**G0F**	*for glycan structures see Figure 2.*
**G0FN**	*for glycan structures see Figure 2.*
**G1F**	*for glycan structures see Figure 2.*
**G1FN**	*for glycan structures see Figure 2.*
**G1FS**	*for glycan structures see Figure 2.*
**G1FNS**	*for glycan structures see Figure 2.*
**G1S**	*for glycan structures see Figure 2.*
**G1NS**	*for glycan structures see Figure 2.*
**G2F**	*for glycan structures see Figure 2.*
**G2FN**	*for glycan structures see Figure 2.*
**G2FS**	*for glycan structures see Figure 2.*
**G2FNS**	*for glycan structures see Figure 2.*
**SE**	standard error
